# Expression of Root Genes in *Arabidopsis* Seedlings Grown by Standard and Improved Growing Methods

**DOI:** 10.3390/ijms18050951

**Published:** 2017-05-03

**Authors:** Yanli Qu, Shuai Liu, Wenlong Bao, Xian Xue, Zhengwen Ma, Ken Yokawa, František Baluška, Yinglang Wan

**Affiliations:** 1 College of Biological Sciences and Biotechnology, Beijing Forestry University, 35 Qinghua East Road, Haidian District, Beijing 100083, China; quyanli@bjfu.edu.cn (Y.Q.); liushuai@bjfu.edu.cn (S.L.); baowenlong@bjfu.edu.cn (W.B.); xuexian@bjfu.edu.cn (X.X.); zhengwenma@yahoo.com (Z.M.); 2 College of Agriculture, Henan University of Science and Technology, Luoyang 471003, China; 3 Department of Biological Sciences, Tokyo Metropolitan University, Tokyo 192-0397, Japan; yokawaken@gmail.com; 4 Institute of Cellular and Molecular Botany, University of Bonn, D-53115 Bonn, Germany; baluska@uni-bonn.de

**Keywords:** *Arabidopsis* roots, improved plant-growing method, transcriptome, photoreceptors, spatial expression

## Abstract

Roots of *Arabidopsis thaliana* seedlings grown in the laboratory using the traditional plant-growing culture system (TPG) were covered to maintain them in darkness. This new method is based on a dark chamber and is named the improved plant-growing method (IPG). We measured the light conditions in dark chambers, and found that the highest light intensity was dramatically reduced deeper in the dark chamber. In the bottom and side parts of dark chambers, roots were almost completely shaded. Using the high-throughput RNA sequencing method on the whole RNA extraction from roots, we compared the global gene expression levels in roots of seedlings from these two conditions and identified 141 differently expressed genes (DEGs) between them. According to the KEGG (Kyoto Encyclopedia of Genes and Genomes) enrichment, the flavone and flavonol biosynthesis and flavonoid biosynthesis pathways were most affected among all annotated pathways. Surprisingly, no genes of known plant photoreceptors were identified as DEGs by this method. Considering that the light intensity was decreased in the IPG system, we collected four sections (1.5 cm for each) of *Arabidopsis* roots grown in TPG and IPG conditions, and the spatial-related differential gene expression levels of plant photoreceptors and polar auxin transporters, including *CRY1*, *CRY2*, *PHYA*, *PHYB*, *PHOT1*, *PHOT2*, and *UVR8* were analyzed by qRT-PCR. Using these results, we generated a map of the spatial-related expression patterns of these genes under IPG and TPG conditions. The expression levels of light-related genes in roots is highly sensitive to illumination and it provides a background reference for selecting an improved culture method for laboratory-maintained *Arabidopsis* seedlings.

## 1. Introduction

Our current knowledge of plant biology is largely based on experiments using the model plant, *Arabidopsis thaliana* [1]. Since *Arabidopsis* was selected and domesticated, methods and medium recipes have been developed to create favorable environments for laboratory culture. However, laboratory-grown *Arabidopsis* typically have roots that are exposed to light. Therefore, it is important to compare this culture technique with a method that would be closer to natural conditions to avoid possible unwanted side effects on the *Arabidopsis* seedlings. To provide a favorable and flexible environment, liquid and solid media-based culture methods were developed. Among these, media based on agar enriched with various salt and vitamins were designed for use in transparent Petri dishes [1]. This is the current standard culture method for *Arabidopsis* seedlings in laboratories [2]. The transparent Petri dish condition allows easy observation of the growth and development of young seedlings but aberrant growth and other stressful side effects occur due to the exposure of roots to light [3,4,5,6,7,8,9,10,11]. This possibility has sometimes been overlooked.

In natural growth conditions, light is an important environmental factor that affects the development and behavior of above- and below-ground plant parts [12,13]. Light perceived by photosensory systems in above-ground tissues can affect the roots via long-distance signal transduction pathways [14,15,16,17]. Since sunlight can reach root tissues growing several centimeters underground [18,19,20], *Arabidopsis* have developed complex photo-sensory systems in the roots to adapt to weak light [21]. Most known plant photoreceptors, including phytochromes, cryptochromes, phototropins, and ultraviolet receptors (UVR), are expressed in the root tissues [21,22]. The light sensory system regulates expression of various related genes under different light intensities and is involved in the development of root structures in response to biotic and abiotic stresses. Silva-Navas et al. found that illumination of roots affects shoot development and flowering time of *Arabidopsis*. This implies that the light sensing systems of roots may influence the entire plant via intercellular signaling [11]. Root illumination also alters the transcriptomic and metabolomic profiles of roots and shoots. Flavonols, which accumulate in the root apical transition zone, can regulate responses to root illumination [23].

The current knowledge of root responses to light clearly shows that the standard culture method requires modification. Several groups have advocated for an improved plant culture method using a shaded/darkened environment for roots that mimics normal underground light conditions. This new growing method has been named the improved plant-growing method (IPG), or the dark-root (D-Root) method compared to the traditional plant-growing method (TPG) [11,23,24]. The only difference in the culture conditions between the IPG and TPG is shading of the roots. Root shading affects the primary root length, density of lateral roots, and root hairs. Altered cellular events, such as stimulation of reactive oxygen species (ROS), pectins, auxin biosynthesis and polar auxin transport, cytokinin biosynthesis and signaling, as well as expression levels of related genes have been reported [4,5,6,7,8,9,10,11,24,25]. Researchers have investigated aspects of growth phenomena in *Arabidopsis*, and reported that tissue-specific expression of photoreceptors in roots affected the shape and development of root tissues [21]. Therefore, a comparison of the full expression levels of functional genes provides useful information related to optimal growth conditions.

In this report we introduce an IPG culture system based on a dark chamber, allowing *Arabidopsis* roots to grow down a light gradient to reach shaded conditions in the relatively darker part of the dark chamber. We measured the changes of light intensities in the IPG system, including the top illumination penetrating through the seed holes and the scattered and reflected light from side and bottom. Global transcriptome analysis was accomplished based on total RNA extraction from roots of 15 d *Arabidopsis* seedlings grown under TPG and IPG conditions. The differently-expressed genes (DEG, corrected *p*-value < 0.005, |log2 (FoldChange)| > 1) were annotated according to the KEGG (Kyoto Encyclopedia of Genes and Genomes) and GO (Gene Ontology) databases to analyze the cellular pathway alterations under TPG and IPG. We quantified the spatial-related gene expression level of photoreceptor genes, including *CRY1*, *CRY2*, *PHYA*, *PHYB*, *PHOT1*, *PHOT2*, and *UVR8.* Based on the qRT-PCR results, we drew a raw map of the expression pattern of these genes under IPG and TPG.

## 2. Results

### 2.1. Root Growing in TPG and IPG Culture Methods

**Figure 1:**
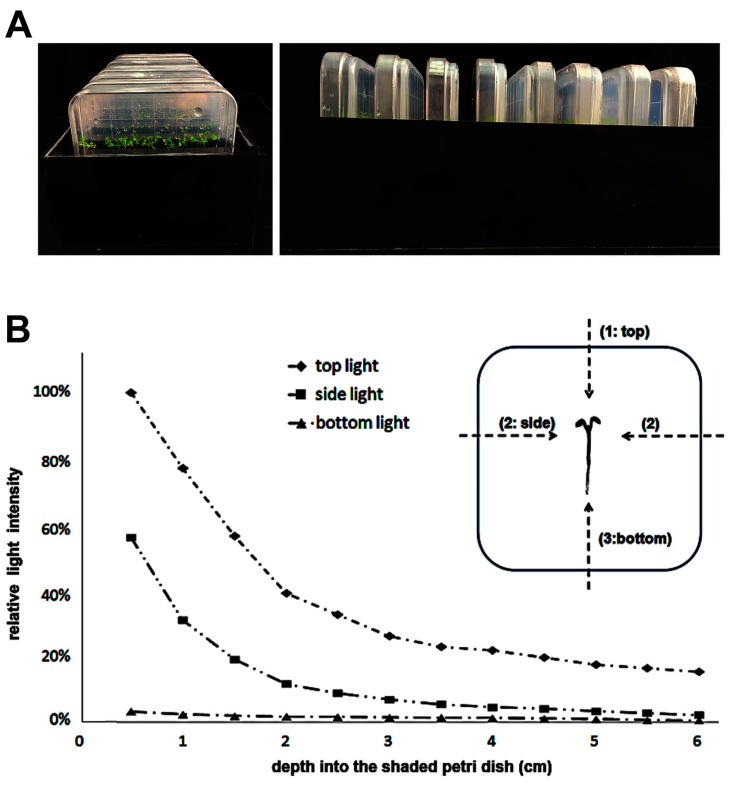
Relative light intensity in the shaded Petri dishes in the IPG growth condition. (**A**) Petri dish holder used for IPG culture in this study; and (**B**) the intensities of light from the top, side, and bottom directions were measured inside the Petri dishes of the IPG culture methods. The intensity of top illumination, 100 μmol·s^−1^·m^−2^, was set as 100% and the relative intensities inside the Petri dish holder were calculated.

We used slightly-modified TPG and IPG culture methods in this study [24]. Sucrose was not added to either the TPG or the IPG media. To simplify the experimental procedure and decrease the contamination rate, we constructed Petri dish holders from non-transparent, black plexiglass (Figure 1A). In the TPG, roots are exposed to light from all directions. There is direct illumination from above and reflected and scattered light from the bottom and sides. We measured the light intensity on the top, sides, and bottom of the TPG and IPG systems to quantify their differences (Figure 1B). The relative light intensity in IPG was determined by setting the light intensity (100 μmol·s^−1^·m^−2^) on the top as 100%. The IPG method completely prevented light illumination from the sides and bottom. Top illumination in IPG varied over a gradient and was greatly reduced compared to TPG (Figure 1B).

### 2.2. Differentially-Expressed Genes (DEGs) in the Roots Grown in IPG and IPG Conditions

**Figure 2:**
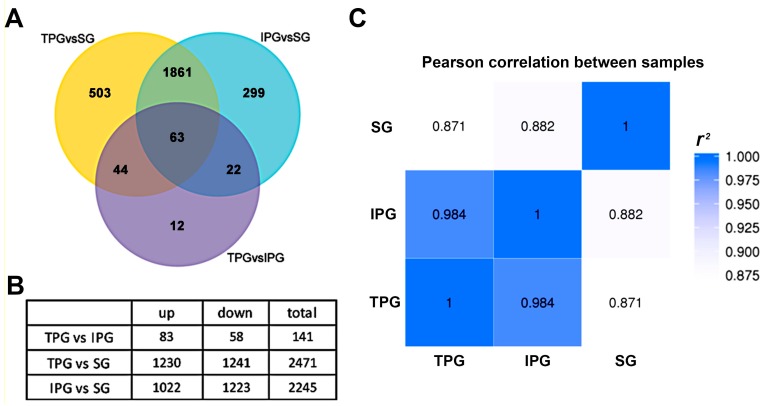
Analysis on RNA-seq results based on the RNA-extraction from *Arabidopsis* roots of three different conditions. (**A**) A Venn diagram shows the numbers of DEGs identified from the total RNA extracted from the roots of soil-growing (SG), traditional plant-growing culture system (TPG), and the improved plant-growing method (IPG). (**B**) The number of up- and downregulated genes from three comparison groups. (**C**) The Pearson correlation analysis between the gene expression pattern of the three different samples.

We compared the TPG and IPG culture methods to the natural soil-grown (SG) method by extracting total RNA from the roots of IPG, TPG, and SG *Arabidopsis* seedlings. More than 3.0 gigabase clean reads were obtained by an Illumina HiSeqTM2000. Gene sequences with RPKM values >1 (reads per kilobase of the exon model per million mapped reads) were counted as effectively-expressed genes. Based on these data, a DEGSeq R package (1.12.0) was used to filter the differently-expressed genes (DEG, corrected *p*-value < 0.005, |log2 (FoldChange)| > 1). We found 2471 DEGs between TPG and SG roots and 2245 DEGs between IPG and SG roots (Supplementary Tables S1 and S2). Only 141 DEGs were identified between roots grown in IPG and TPG conditions (Figure 2A,B, Supplementary Table S3). Pearson correlation analysis [26] showed that the gene expression patterns of IPG and TPG roots were very similar (*r*^2^ = 0.984), while the SG roots had a much different expression pattern. The Pearson correlation between TPG and SG (*r*^2^ = 0.871) was lower than the Pearson correlation between IPG and SG (*r*^2^ = 0.882), implying a slightly greater similarity in the IPG condition (Figure 2C). SG expression was very different from the Petri dish-based methods, but the gene expression pattern in the IPG roots was similar to the TPG roots. Therefore, we focused on the 141 DEGs between IPG and TPG roots and the metabolic pathways that appeared to be most affected (Supplementary Table S3).

To validate the profiling of the DEG analysis, we randomly selected 10 genes from the 63 common DEGs from the IPG vs. TPG, TPG vs. SG, and IPG vs. SG comparison groups. The quantitative results from the q-RTPCR analysis were closely correlated with the RNA-sequencing data. The primers designed for the q-RTPCR analysis were listed in Supplementary Table S4. The correlation coefficients among these three groups were 0.937 (TPG vs. IPG), 0.628 (TPG vs. SG), and 0.900 (IPG vs. SG), respectively (Supplementary Figure S1).

### 2.3. GO Enrichment Analysis of DEGs between IPG and TPG Roots

**Figure 3:**
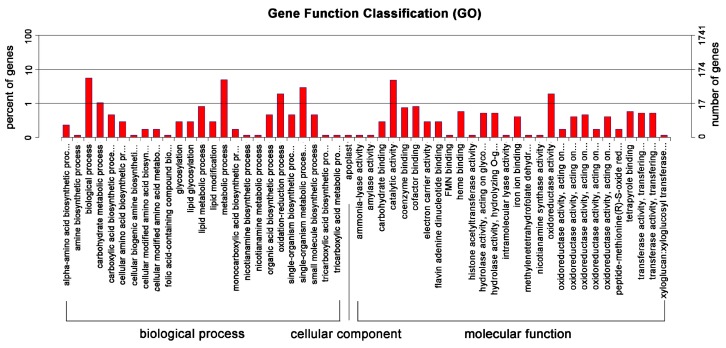
Classification of the DEGs from the IPG/TPG comparison group into GO terms. Forty-one annotated DEGs from the IPG/TPG groups were classified into the GO categories to show the most affected gene functions in these two growth conditions.

To illustrate the biological functions of the identified DEGs, we performed an enrichment analysis and categorized them into GO terms. In the TPG vs. IPG comparison group, 83 of DEGs were upregulated and 58 genes were downregulated in TPG (Figure 2B, Supplementary Table S1). Most of the 141 DEGs were enriched into two main GO categories: biological process (BP) and molecular function (MF), while the category “cellular components” (CC) has only one affected group, namely apoplast (Figure 3). The GO terms single-organism metabolic process, biological process, metabolic process, oxidation-reduction process, and carbohydrate metabolic process were the five largest gene groups among the BP category (Table 1). The GO terms oxidoreductase activity, catalytic activity, coenzyme binding, cofactor binding, and heme binding were the five largest gene groups among the MF category (Table 1). 

### 2.4. Identification and Classification of the DEGs in the KEGG Pathways

To study the effects of altered light conditions on DEGs of *Arabidopsis* roots, we tested the enrichment of DEGs in KEGG pathways. A total of 141 DEGs between IPG and TPG were grouped into 43 KEGG pathways (Supplementary Table S1). Among these, the “flavone and flavonol biosynthesis”, “flavonoid biosynthesis”, and “α-linolenic acid metabolism” were the three most affected pathways, with the highest rich factors of 0.6, 0.22, and 0.16, respectively (Table 2).

In the KEGG database, 256 genes were categorized into eight plant hormone signal pathways, including auxin, cytokinie, gibberellins, abscisic acid (ABA), jasmonic acid (JA), and ethylene, Brassinosteroid (BR), and salicylic acid (SA). Between the *Arabidopsis* seedlings grown under IPG and TPG culture conditions, roots have six DEGs distributed in the KEGG-defined plant hormone signaling pathways. Among them, the JA signaling pathway has five DEGs and the ABA signaling pathway has only one DEG (Table 3).

### 2.5. Different Expression Pattern of Plant Photoreceptor Genes

**Figure 4:**
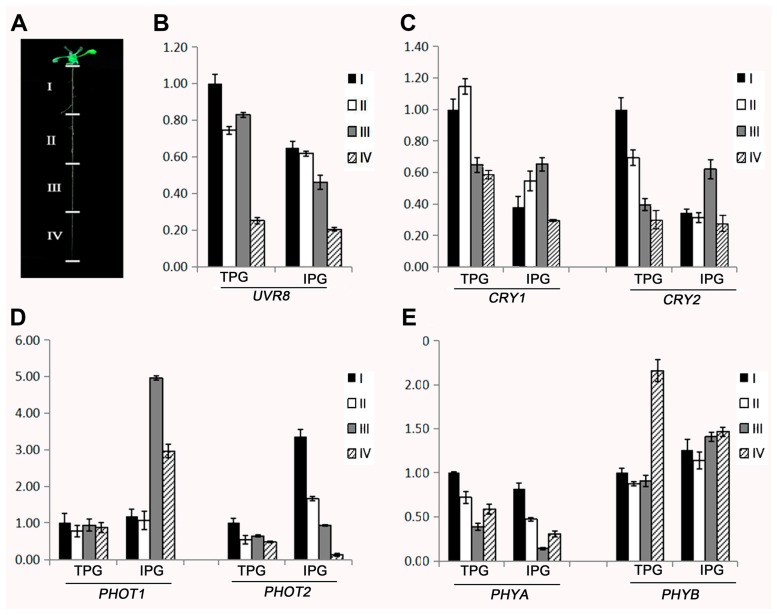
Expression patterns of seven plant photoreceptors in *Arabidopsis* roots grown in TPG and IPG conditions. (**A**) An image showing four sections from the roots of *Arabidopsis* with 1.5 cm length for each section. (**B**–**E**) Total RNAs were extracted from each section and qRT-PCR was used to quantify the relative expression level of target genes. The expression levels of each gene in Section I of TPG roots were set as 1. The relative expression of all of these genes was presented. The expression of *UVR 8* is (**B**), *CRY1* and *CRY2* is (**C**), *PHOT1* and *PHOT2* is (**D**), and *PHYA* and *PHYB* is (**E**).

In the TPG and IPG comparison, no photoreceptor genes were identified as DEGs. However, it has been reported that the expression of several photoreceptors in roots are light-dependent [21]. To verify the different expression of photoreceptors in the TPG and IPG treatments, and how they are differently expressed, we investigated spatially-related expression levels of seven main photoreceptors in the root sections. We collected 4 cm long roots of healthy *Arabidopsis* seedlings. Roots were cut into 1-cm-long sections and the total RNA was extracted. Sections from the upper to lower positions are listed as Sections I to IV (Figure 4A). Using the quantitative reverse transcriptase PCR (q-RTPCR) technique, we examined the gene expression level of the UV-B receptor *UV-B resistance 8 (UVR8*), blue light, and UV-A receptors, *CRYPTOCHOME 1(CRY1)*, *CRYPTOCHOME 2(CRY2)*, *PHOTOTROPIN1 (PHOT1)*, *PHOTOTROPIN2 (PHOT2)*, and the red light receptors, *PHYTOCHROME* *A* *(PHYA)*, *PHYTOCHROME B* *(PHYB)*. The primers designed for the q-RTPCR analysis are listed in Supplementary Table S5. We conducted at least three independent experiments and >30 different roots were collected for each experiment. Experimental results had only minor variation and the relative expression patterns were similar in all repetitions. In these comparisons, the expression levels of genes in Section I of TPG roots were set as standards.

For the *UVR8* in TPG, the expression levels gradually decreased from Section I to Section IV. The expression levels in Sections II and III were not significantly different. In the IPG, the expression of UVR8 in the root decreased from Section I to Section IV. The expression of UVR8 in IPG roots was lower than in the TPG roots (Figure 4B).

For the blue/UV-A light receptors *CRY1* and *CRY2*, relative expression levels of both genes were changed between TPG and IPG roots in similar ways. *CRY1* expression in the TPG roots gradually decreased from Section I to Section IV. In the TPG roots, *CRY1* expression levels in Sections I and II were similar, while expression levels in Sections III and IV were similar. The IPG roots had decreased *CRYI* expression levels in Sections I, II, and IV, resulting in a top expression level on *CRYI* in Section III. *CRY2* expression in TPG roots decreased sharply from Section I to Section IV. IPG roots had a different expression pattern of *CRY2* with significant expression in Section III and significantly decreased expression levels in Sections I and II (Figure 4C).

For the blue/UVA light receptors *PHOT1* and *PHOT2*, relative expression levels of both genes were significantly different in TPG and IPG roots (Figure 4D). In the TPG roots, the *PHOT1* gene was constantly expressed through the entire root, while the IPG roots had significantly increased *PHOT1* expression in Sections III and IV of the root tip region. *PHOT2* expression in TPG roots slightly decreased from Section I to Section IV. *PHOT2* expression in IPG roots sharply decreased from Section I to Section IV. *PHOT2* expression in Section I of IPG roots was much greater than in TPG roots. In contrast, the expression level of *PHOT2* in Section IV was much greater in TPG than in IPG.

In the red light receptors, the gene expression pattern of *PHYA* in the TPG roots was similar to that in the IPG roots, but the PHYB expression pattern was significantly different (Figure 4E). Section I had the highest *PHYA* expression level, and the *PHYA* expression level gradually decreased from Section I to Section III. The relative expression levels of *PHYA* in the root tip region increased. The expression patterns of *PHYB* in IPG roots and TPG roots differed. In the TPG roots, *PHYB* expression was uniformly distributed from Section I to Section III, and Section IV had significantly increased *PHYB* expression. In the IPG roots, *PHYB* was expressed uniformly in all root sections. In the TPG roots, the relative expression level of *PHYB* was constant from Section I to III. However, the root tip region has a significant increase in *PHYB* expression.

## 3. Discussion

Several groups have suggested that *Arabidopsis* seedlings should be cultured using agar plates with shaded/darkened root system providing more natural culture conditions [4,5,6,7,8,9,11,12,13,23,24]. We used the standard and improved laboratory culture systems and measured the light intensities under the dark border. We detected weak light signals several centimeters beneath the dark border. This occurred because the Phytagel in the agar plate is light transparent. Moreover, the refracted and reflected light in the TPG method gives roots light signals from all directions. The unnatural illumination on the roots grown in TPG plates is caused by the high light intensities and also by the unnatural direction of light striking the roots.

For 15-day-old seedlings in IPG, the primary roots were significantly longer and there were fewer lateral roots and less root hair than the roots of 15 d plants grown in TPG [24]. The measured lengths of primary roots in this study were similar to earlier reports [24]. Therefore, we made a global transcriptome analysis based on the total RNA extracted from 15-day-old TPG-, IPG-, and SG-grown *Arabidopsis* roots. A strict definition was set to filter the DEGs: a gene having at least a two-fold changed expression level and a corrected *p*-value < 0.005 was recognized as a DEG. Unsurprisingly, the expression patterns of genes between the two agar plate-based methods were highly correlated, while SG caused a heavy altered gene expression pattern that contrasted with the IPG and TPG results. SG is an obviously different environment for *Arabidopsis* and this has often been noted. Therefore, focusing on the 141 DEGs between the roots grown in IPG and TPG conditions will provide useful background information for research that uses these methods.

According to the GO functional groups, all 141 DEGs were categorized into the “biological process” and “molecular function” main groups. However, no DEG was categorized into the “cellular components” GO group. The most enriched GO terms were related to the metabolic pathways and catalytic pathways, revealing that the altered root light conditions directly changed the growth rate and metabolic rate. However, the cellular structures might have been less affected. When we enriched these DEGs into the KEGG pathways to reveal the most affected metabolic processes, we found that the pathways related to biosynthesis of flavone and flavonol components were most heavily influenced, with three of five background genes identified as DEGs. This result is consistent with a report demonstrating that gene expression of the flavonol biosynthesis pathway is activated by UV-B irradiation [27]. Sustained illumination causes high levels of flavonols. Lateral illumination further induced the accumulation of flavonols in the lighted side of roots, causing negative root phototropic bending [23]. Since UV-B has a relatively short wavelength with a relatively shallow penetration depth, the shadowed growth condition has the greatest effect on the expression of the UV-related gene.

The structure and development of roots is regulated by the synthesis and distribution of phytohormones [28]. According to the KEGG database, there are 265 genes registered into the plant hormone pathways. We found that six DEGs in the TPG vs. IPG comparison group were enriched in this pathway. Only one DEG (*SnRK2*) is involved in the JA signal transduction pathway. This was up-regulated in IPG. Moreover, five DEGs were found in the ABA pathway, including four genes encoding the JASMONATE ZIM-domain (JAZ) proteins (*JAZ1*, *JAZ2*, *JAZ6*, *JAZ10*) and one transcription factor encoding gene *MYC2.* Interestingly, we did not find a DEG gene in the auxin signaling pathway between IPG and TPG. Since the *PIN* genes that encode important auxin efflux carrier PIN-formed proteins [29] were not included in the KEGG pathways, we analyzed their expression levels as well. No *PIN* genes were DEGs. Another important auxin transporter family, *ABCBs*, also had no DEGs. This result conflicts with a report that the expression level of PIN2 was significantly different between IPG and TPG growth [24]. This conflicting result may be caused by glucose in the TPG media used in the study since glucose can act as a signal molecule to mediate gene expression.

To understand how the roots under the dark-border detect the weak light penetrating underneath the dark border, we also analyzed the expression pattern of 14 known *Arabidopsis* photoreceptors. No photoreceptor genes were defined as DEGs in roots grown in IPG and TPG. In contrast, early reports suggested that the expression level of phytochromes [30,31], cryptochrome [3], and phototropins [32] in roots is light dependent. For example, the expression level of *PHOT1* in roots depends on the depth of roots grown beneath the soil surface [32]. However, effects of the gradient-decreased light intensity on the root photoreceptor expression were not reported. We extracted RNAs from whole roots and defined the DEG from the RNA sequencing results using a rigorous standard which may have filtered out some useful information.

Therefore, we analyzed seven abundantly expressed photoreceptor genes in 1.5 cm sections of roots grown in both IPG and TPG conditions. In TPG, *UVR8*, *CRY1*, *CRY2*, *PHOT2*, and *PHYA* had decreased expression from Section I to Section IV. IPG did not change the expression level and expression pattern of *PHYA*. The expression level of *UVR 8* was decreased in IPG roots without changing the pattern, while the expression of *PHOT2* was increased without changing its expression pattern. In the blue light receptor genes *CRY1* and *CRY2*, both expression level and expression pattern were affected. *PHOT1* in TPG roots had a uniform expression through Sections I–IV. Shaded roots significantly increased the expression of *PHOT1* in Sections III and IV. Earlier reports about the spatially-separated expression level of *PHOT1* were related to the initiation of lateral roots [32], indicating that the gradient of photoreceptors expression under the soil surface may reflect a physical response in the roots. *PHYB* showed a dramatically changed expression pattern, the TPG roots had a high level of *PHYB* in root tips, and this peak expression was absent in IPG. We found that, even though the total expression level of the photoreceptor genes were not classified into the DEGs from the transcriptomic analysis between IPG and TPG roots, the light gradient created by the IPG system altered the spatial gradient of expression pattern of these genes. However, the exact physiological roles are still unknown.

In conclusion, the different expression levels of *Arabidopsis thaliana* genes in roots grown by TPG and IPG provides information on the regulation of gene expression under decreased light conditions. In laboratory studies, the light conditions used for plant growth must be carefully considered. We determined the DEGs present in the TPG and IPG conditions and found that most DEGs affected aspects of root metabolism pathways. Analysis of the spatially different expression patterns of photoreceptors will provide background information about their potential effects on morphological and physiological phenotypes.

## 4. Materials and Methods

### 4.1. Plant Sampling

Wild-type seedlings of *Arabidopsis thaliana* ecotype Columbia-0 (Col-0) were studied. The *Arabidopsis* seeds were planted on half-strength (2.15 g/L), Murashige and Skoog-containing medium (Duchefa Biochemie, Haarlem, The Netherlands) with 0.4% Phytagels (Sigma-Aldrich, Munich, Germany). The TPG and IPG growing conditions are shown in Figure 1. All seeds were germinated and grown at 22–24 °C under a 16:8 h (L:D) photoperiod. Four 21 watt white fluorescent lamps (FLS, Foshan, China) were set on the top of the culture chamber with 40 cm distance to the top of the Petri dishes. The light intensity was measured by a photo meter model LP-1 (Sanwa, Tokyo, Japan), with the light sensor facing the top, side, and bottom inside an empty Petri dish to measure the light intensities under the dark border created by the dark box. In addition to the TPG and IPG plants, *Arabidopsis* seeds were also planted in soil and cultivated in the same incubator, these condition were named “soil grown” (SG). When plants were 15 days old, we sampled the whole root for transcriptome sequencing. For spatially-controlled expression analysis, we sampled the 15-day-old roots with the same length (4 cm) by equally dividing the root into four sections, 1 cm for each section. All specimens were immediately frozen in liquid nitrogen and stored at −80 °C until analysis.

### 4.2. Library Preparation for Transcriptome Sequencing

We used poly-T oligo-attached magnetic beads to purify mRNA from the total RNA. RNA yield and fragment insert size quality were assessed on an Agilent Bioanalyzer 2100 system (Agilent, Santa Clara, CA, USA). Sequencing libraries were generated using NEBNext^®^ Ultra™ RNA Library Prep Kit for Illumina^®^ (NEB, Ipswich, MA, USA) following the manufacturer’s instructions. Sequencing was performed by Beijing Novogene Bioinformatics Technology Co. Ltd., Beijing, China.

### 4.3. Clustering and Sequencing

The clustering of the index-coded samples was performed on a cBot Cluster Generation System using a TruSeq PE Cluster Kit v3-cBot-HS (Illumina, San Diego, CA, USA) according to the manufacturer’s instructions. After cluster generation, the library preparations were sequenced on an Illumina Hiseq platform and 125 bp/150 bp paired-end reads were generated.

### 4.4. Quality Control

The Q20, Q30, and GC content of clean data were calculated. Clean data (clean reads) were obtained by removing reads containing adapters, reads containing ploy-*N*, and low quality reads from raw data. All of the downstream analyses were based on high quality, clean data.

### 4.5. Differential Expression Analysis

Differential expression analysis of TPG/IPG was performed using the DESeq R software package (1.18.0) [33]. DESeq provides statistical routines for determining differential expression in digital gene expression data using a model based on the negative binomial distribution. The resulting *p*-values were adjusted using the Benjamini-Hochberg approach for controlling the false discovery rate [33]. Genes with an adjusted *p*-value < 0.05 found by DESeq were assigned as differentially expressed.

### 4.6. Reads Mapping to the Reference Genome and Functional Annotation

*Arabidopsis* reference genome and gene model annotation files were downloaded directly from the genome website (TAIR 10). The index of the reference genome was constructed using Bowtie v2.2.3 and paired-end clean reads were aligned to the reference genome using TopHat v2.0.12 [34].

### 4.7. GO and KEGG Enrichment Analysis of Differentially Expressed Genes

Gene Ontology (GO) enrichment analysis of differentially-expressed genes was implemented by the GOseq R package, to correct the gene length bias. GO terms with corrected *p*-values < 0.05 were considered significantly enriched by differentially-expressed genes (GO, http://www.geneontology.org/).

KEGG is a database used for understanding high-level functions and utilities of the biological system, such as the cell, the organism, and the ecosystem. This is often used for molecular-level information, especially large-scale molecular datasets generated by genome sequencing and other high-throughput technologies (http://www.genome.jp/kegg/). We used KOBAS software (Kobas, London, UK) to conduct statistical testing of the enrichment of differentially-expressed genes in KEGG pathways.

### 4.8. Total RNAs Extraction

Total RNAs were extracted from *Arabidopsis* using an RNAprep Pure Plant Kit (TIANGEN, Beijing, China). According to the manufacturer’s protocol, we first obtained the total RNAs and then the RNA samples were examined by 2.5% agarose (Biowest, Nuaillé, France) gel electrophoresis for 10 min.

### 4.9. cDNA Synthesis

Complementary DNA (cDNA) was synthesized using a Fast Quant RT Kit (TIANGEN, Beijing, China), following the manufacturer’s protocol. The reverse transcription system was based on 5 μg of total RNA, which generated approximately 20 μL of cDNA by using random primers. The resulting cDNAs were diluted to a ratio of 1:5 with nuclease-free water. All of the cDNAs were stored at −20 °C until analysis.

### 4.10. Primer Design

We obtained the sequences of targeted genes from The *Arabidopsis* Information Resource (TAIR). The selected genes were used in designing primers using Primer Premier 5 (Premier, Palo Alto, CA, USA). The length of the amplified fragments ranged from 150 to 200 bp. Control cDNAs were used as a template to test pairs of primers by PCR to verify that they were usable.

### 4.11. Quantitative Real-Time PCR (qRT-PCR)

The qRT-PCR method was implemented using SuperReal PreMix Plus with SYBR Green (TIANGEN, Beijing, China) on a CFX96 real-time PCR detection system (Bio-Rad, Hercules, CA, USA). All cDNA templates used in the experiment were of the same concentration. The 20 μL reaction systems were prepared using the following: 1 μL of the cDNA template, 7.4 μL of water, 10 μL of 2× SuperRealPreMix Plus, 0.4 μL of 50× ROX reference dye, and 0.6 μL of the forward and reverse primers. The PCR program used a two-step process that was run for: 95 °C for 10 min, then denaturation at 95 °C for 10 s with 40 cycles, annealing at 60 °C for 32 s, and extension at 72 °C for 10 s. Each reaction had four replicates. Melting curve data were obtained from 65 to 95 °C in 0.5 °C increments.

## Figures and Tables

**Table 1:** List of the five most effected GO accessions in each GO categories.

Main GO Categories	GO Accession	Description
Biological Process(BP)	GO:0044710	Single-organism metabolic process
	GO:0008150	Biological process
	GO:0008152	Metabolic process
	GO:0055114	Oxidation-reduction process
	GO:0005975	Carbohydrate metabolic process
Molecular Function(MF)	GO:0016491	Oxidoreductase activity
	GO:0003824	Catalytic activity
	GO:0050662	Co-enzyme binding
	GO:0048037	Cofactor binding
	GO:0020037	Heme binding

**Table 2:** The DEGs involved in three KEGG pathways with the highest rich factor *.

KEGG Pathways	Pathways ID	Rich Factor	Genes ID	Gene Name	Log_2_ Fold Change (TPG vs. IPG)	*q*-Value
Flavone and flavonol biosynthesis	ath00944	0.6	AT5G17050	*UGT78D2*	1.3328	1.85 × 10^−16^
			AT1G30530	*UGT78D1*	1.5333	7.23 × 10^−5^
			AT5G07990	*CYP75B1*	2.1337	6.72 × 10^−57^
α-Linolenic acid metabolism	ath00592	0.22	AT5G48880	*KAT5*	1.5858	1.68 × 10^−29^
			AT1G20510	*OPCL1*	−1.0607	4.72 × 10^−7^
			AT2G06050	*ATOPR3*	−1.0431	0.0044624
			AT1G17420	*ATLOX3*	−2.0906	0.00038411
			AT5G42650	*AOS*	−1.2413	1.74 × 10^−6^
Flavonoid biosynthesis	ath00941	0.16	AT5G07990	*CYP75B1*	2.1337	6.72 × 10^−57^
			AT5G13930	*ATCHS*	1.0895	5.86 × 10^−28^
			AT5G08640	*FLS1*	1.2799	3.77 × 10^−85^
			AT3G55120	*ATCHI*	1.5173	1.7 × 10^−49^

* Rich factor = (gene numbers annotated as DEG)/(total gene numbers listed in the KEGG pathway).

**Table 3:** List of the DEGs involved in two KEGG pathways related to the phytohormon metabolisms.

KEGG Pathways	DEG Number	Gene ID	Gene Name	Log_2_ Fold Change (TPG vs. IPG)	*q*-Value
Jasmonic Acid	5	AT1G19180	*ATJAZ1*	−1.0859	2.99 × 10^−26^
		AT1G32640	*ATMYC2*	−1.005	3.77 × 10^−19^
		AT1G72450	*JAZ6*	−1.5942	2.76 × 10^−9^
		AT1G74950	*JAZ2*	−2.4038	5.14 × 10^−20^
		AT5G13220	*JAS1*	−2.861	0.002773
Abscisic Acid	1	AT1G78290	*SRK2C*	1.2903	2.06 × 10^−5^

## Supplementary Materials

The following are available online at http://www.mdpi.com/1422-0067/18/5/951/s1.
