# Supplementary material for: Expression of Root Genes in Arabidopsis Seedlings Grown by Standard and Improved Growing Methods
Source: Int J Mol Sci. 2017 May 3;18(5):951. doi: 10.3390/ijms18050951 (PMC5454864; doi:10.3390/ijms18050951)
Supplement: Supplementary file 1 [file ijms-18-00951-s001.zip › Table S1.pdf]

Supplementary Table 1. List of all 2245 DEGs from IPG/SG comparison groups.

Gene_id	readcount_IPG	readcount_IPG	log_2 Fold change (IPG vs SG)	q value
AT5G22890	28.17093568	0	9.2233	1.01E–05
AT1G51840	26.87572025	0	8.1554	3.71E–06
AT1G51830	114.6265659	0	7.663	7.41E–26
AT5G23990	18.78062379	0	7.6383	0.00010944
AT5G07990	110.7871773	0	7.2919	8.63E–26
AT2G24762	40.01290536	0	6.4076	4.39E–10
AT1G73120	15.86638906	0	6.395	0.00023408
AT3G46900	10.40798116	0	6.2018	0.0042396
AT5G59520	71.42187962	0	6.1731	8.35E–18
AT4G29740	18.22553146	0	5.7877	5.61E–05
AT5G17040	17.80921221	0	5.7543	7.03E–05
AT5G04950	41.30812079	0	5.688	1.45E–10
AT3G28345	132.7133242	2.59230539	5.6779	4.64E–33

AT1G08430	10.36172347	0	5.4584	0.0041332
AT2G25090	17.48540835	0	5.3653	8.51E–05
AT5G26270	27.10700872	0	5.2608	4.25E–07
AT4G25100	210.7500541	5.514540558	5.2561	3.67E–52
AT5G48010	339.8090205	9.332299405	5.1863	6.96E–84
AT5G35190	40.75302846	1.178320632	5.1121	2.37E–10
AT4G12510	23.63768166	0	4.9701	3.30E–06
AT5G48000	252.8908134	8.201111598	4.9466	6.28E–62
AT1G31320	12.62835048	0	4.8958	0.0013444
AT5G03995	14.98749287	0	4.8534	0.00038436
AT5G50760	46.76652869	1.649648885	4.8252	1.13E–11
AT3G08040	65.45463709	2.309508439	4.8248	3.62E–16
AT3G56290	34.32320899	1.272586283	4.7533	1.15E–08
AT3G09220	129.9378626	4.996079479	4.7009	1.67E–31
AT4G04750	16.65276986	0	4.6575	0.00017379
AT4G12520	106.9477887	4.430485576	4.5933	8.50E–26

AT3G14060	21.55608543	0	4.5892	1.34E–05
AT3G49330	13.36847358	0	4.5629	0.0010473
AT4G31940	11.6569389	0	4.5431	0.0025919
AT4G11210	19.70577767	0	4.5377	3.73E–05
AT5G47980	19.70577767	0	4.5377	3.73E–05
AT4G31330	11.93448507	0	4.5248	0.0022617
AT1G66725	36.45106292	1.602516059	4.5076	5.05E–09
AT4G37310	24.60909324	1.084054981	4.5047	2.86E–06
AT2G25160	32.33412815	1.461117584	4.4679	4.91E–08
AT5G47990	194.7911497	8.813838327	4.466	2.86E–46
AT3G26290	14.29362746	0	4.4371	0.00069633
AT1G64500	14.24736977	0	4.4324	0.00071582
AT4G37160	15.26503904	0	4.4324	0.00041935
AT1G54970	11.98074276	0	4.4048	0.0023542
AT5G04960	23.73019705	1.131187807	4.3908	5.18E–06
AT5G62210	36.31228983	1.791047361	4.3416	7.28E–09

AT3G62680	32.88922047	1.649648885	4.3174	4.66E–08
AT1G65970	118.234666	5.938735985	4.3154	8.21E–28
AT2G03090	116.8931929	6.033001636	4.2762	2.17E–27
AT5G47950	27.19952411	1.413984758	4.2657	9.86E–07
AT1G80340	18.04050068	0	4.2584	0.00011523
AT3G61220	57.45205602	3.016500818	4.2514	1.30E–13
AT3G45710	100.703	5.326009256	4.2409	1.47E–23
AT4G34881	618.9742042	32.75731357	4.24	1.60E–143
AT2G23910	134.3786012	7.541252044	4.1554	5.04E–31
AT1G78990	24.33154707	1.366851933	4.1539	5.17E–06
AT4G08400	24.74786632	1.413984758	4.1295	4.31E–06
AT4G29905	250.6241864	14.32837888	4.1286	1.84E–57
AT5G10230	23.82271244	1.366851933	4.1234	7.00E–06
AT5G66580	13.09092742	0	4.1176	0.0016248
AT3G01260	32.657932	1.885313011	4.1146	7.69E–08
AT1G77990	22.85130086	1.319719108	4.114	1.17E–05

AT3G49960	12.16577354	0	4.105	0.0026093
AT4G04840	30.94639732	1.838180186	4.0734	2.00E–07
AT5G38100	14.20111208	0	4.0651	0.0009773
AT3G44990	182.9029223	10.93481546	4.0641	1.18E–41
AT3G57020	257.6553559	15.69523082	4.037	2.12E–58
AT2G46420	19.93706614	1.225453457	4.0241	5.77E–05
AT4G08410	38.99523609	2.450906914	3.9919	4.03E–09
AT1G64170	28.95731648	1.838180186	3.9776	6.60E–07
AT5G13900	24.65535093	1.649648885	3.9017	6.59E–06
AT5G57785	28.17093568	1.885313011	3.9013	1.14E–06
AT4G08040	19.56700459	1.319719108	3.8901	8.35E–05
AT1G32450	306.31845	21.8224981	3.8111	3.32E–67
AT3G45700	23.68393936	1.69678171	3.803	1.28E–05
AT4G01680	13.13718511	0	3.8008	0.0021101
AT2G25000	15.03375057	1.084054981	3.7937	0.00085861
AT5G20700	322.5549007	23.56641264	3.7747	2.39E–70

AT5G66690	139.8370091	10.22782309	3.7732	1.29E–30
AT4G00880	49.54199033	3.629227546	3.7709	4.27E–11
AT2G01530	614.1171463	45.43604357	3.7566	1.99E–133
AT5G08640	613.9783732	45.48317639	3.7548	2.31E–133
AT1G51860	46.99781716	3.487829071	3.7522	1.61E–10
AT3G46130	22.01866237	1.649648885	3.7385	3.20E–05
AT2G40230	14.70994671	1.131187807	3.7009	0.0011159
AT2G39310	230.6408626	17.76907513	3.6982	1.06E–49
AT5G13930	2386.989529	185.5148003	3.6856	0
AT1G16400	52.73377122	4.147688624	3.6683	1.37E–11
AT3G58990	50.18959805	3.959157323	3.6641	4.85E–11
AT2G36090	16.42148139	1.319719108	3.6373	0.00054116
AT1G22750	49.68076342	4.006290149	3.6323	7.09E–11
AT1G35515	12.58209278	1.036922156	3.601	0.0033765
AT3G25930	108.4280349	8.955236803	3.5979	4.11E–23
AT3G09260	11533.93969	955.1938371	3.5939	0

AT1G78090	61.80027926	5.137477955	3.5885	2.59E–13
AT4G28250	115.6442351	9.662229182	3.5812	1.51E–24
AT1G64780	183.4117569	15.36530104	3.5773	1.01E–38
AT3G59340	22.85130086	1.932445836	3.5638	3.01E–05
AT4G37760	34.83204362	2.969367993	3.5522	1.13E–07
AT5G49080	28.72602801	2.450906914	3.551	1.99E–06
AT1G14160	18.22553146	1.555383234	3.5506	0.00026417
AT2G29340	14.89497749	1.272586283	3.549	0.001241
AT4G15393	74.93746437	6.410064238	3.5473	6.17E–16
AT1G70890	32.42664353	2.780836691	3.5436	3.56E–07
AT5G19600	23.59142397	2.026711487	3.541	2.23E–05
AT2G04090	14.20111208	1.225453457	3.5346	0.0017358
AT4G04745	16.23645061	1.413984758	3.5214	0.00069536
AT4G13390	44.31487091	3.864891673	3.5193	1.46E–09
AT5G26310	18.87313917	1.649648885	3.5161	0.00020794
AT3G09270	284.1147569	24.83899892	3.5158	3.63E–59

AT3G25190	258.6730252	22.85942026	3.5003	1.05E–53
AT4G15290	16.05141984	1.461117584	3.4576	0.00083244
AT4G22460	14.38614285	1.319719108	3.4464	0.0017941
AT4G28740	13.27595819	1.225453457	3.4374	0.002976
AT5G09520	28.58725493	2.639438216	3.4371	2.86E–06
AT4G20240	22.94381625	2.120977138	3.4353	3.78E–05
AT2G18050	41.40063618	3.864891673	3.4212	8.44E–09
AT5G36180	37.19118602	3.487829071	3.4146	6.00E–08
AT1G14960	40.15167844	3.770626022	3.4126	1.56E–08
AT5G47110	52.59499814	4.996079479	3.3961	5.57E–11
AT5G48880	127.162401	12.16026892	3.3864	6.32E–26
AT4G39770	22.11117776	2.120977138	3.382	6.21E–05
AT1G12740	60.96764077	5.89160316	3.3713	1.35E–12
AT1G55990	54.67659437	5.373142082	3.3471	2.79E–11
AT4G12330	12.4433197	1.225453457	3.344	0.0048733
AT3G23730	22.94381625	2.262375613	3.3422	4.67E–05

AT2G28470	71.42187962	7.069923792	3.3366	1.43E–14
AT4G31910	59.81119841	6.033001636	3.3095	3.37E–12
AT1G49310	56.29561367	5.703071859	3.3032	1.71E–11
AT3G20370	258.1179328	26.39438216	3.2897	3.29E–51
AT4G17280	47.22910563	4.854681004	3.2822	1.14E–09
AT5G48290	35.15584748	3.629227546	3.276	2.49E–07
AT4G35030	30.94639732	3.205032119	3.2714	1.62E–06
AT1G78370	32.657932	3.39356342	3.2666	7.69E–07
AT1G60270	39.78161689	4.147688624	3.2617	3.42E–08
AT2G48130	124.5719701	13.0557926	3.2542	1.17E–24
AT2G24980	76.60274136	8.059713123	3.2486	2.71E–15
AT1G22430	16.51399678	1.743914535	3.2433	0.00095852
AT5G41040	124.3406816	13.15005825	3.2412	1.54E–24
AT3G01420	255.5737597	27.05424171	3.2398	4.13E–50
AT3G02885	17.53166605	1.885313011	3.2171	0.00064725
AT5G62920	17.67043913	1.932445836	3.1928	0.00063889

AT5G01250	13.78479283	1.508250409	3.1921	0.0033221
AT1G34040	34.73952824	3.817758848	3.1858	4.17E–07
AT2G43100	46.720271	5.137477955	3.1849	2.30E–09
AT4G22666	38.9489784	4.336219926	3.1671	7.32E–08
AT2G38380	561.6146635	62.87518892	3.159	8.66E–108
AT2G34610	15.5425852	1.743914535	3.1558	0.0016771
AT5G06330	16.328966	1.838180186	3.1511	0.0012179
AT5G54230	31.36271657	3.534961896	3.1493	2.05E–06
AT1G52070	87.28826868	9.945026134	3.1337	6.97E–17
AT5G06640	101.8131846	11.64180784	3.1285	1.31E–19
AT1G13250	13.9698236	1.602516059	3.1239	0.0034042
AT3G48100	37.70002066	4.336219926	3.1201	1.53E–07
AT5G37690	55.13917132	6.362931413	3.1153	8.93E–11
AT2G22920	50.42088652	5.844470334	3.1089	7.07E–10
AT1G73620	18.6418507	2.168109963	3.104	0.00050538
AT4G35060	48.38554798	5.655939033	3.0967	1.79E–09

AT4G29140	22.43498162	2.639438216	3.0874	0.00010759
AT4G05200	17.57792374	2.073844312	3.0834	0.00082131
AT4G21230	19.65951997	2.356641264	3.0604	0.00036345
AT1G28130	48.66309415	5.844470334	3.0577	1.98E–09
AT4G26850	82.38495311	9.897893308	3.0572	1.22E–15
AT5G26010	18.41056223	2.215242788	3.055	0.00061681
AT1G13080	29.32737803	3.534961896	3.0525	6.72E–06
AT5G62720	29.5586665	3.582094721	3.0447	6.29E–06
AT1G65060	72.30077581	8.766705502	3.0439	1.01E–13
AT3G49120	197.612869	23.99060807	3.0421	7.09E–37
AT1G03820	30.66885116	3.723493197	3.042	3.99E–06
AT2G01520	949.1616243	115.8524845	3.0344	5.07E–176
AT5G53110	28.91105879	3.534961896	3.0319	8.62E–06
AT5G66460	15.03375057	1.838180186	3.0319	0.0025565
AT2G37460	19.56700459	2.403774089	3.025	0.00040738
AT2G43920	258.3029636	32.09745401	3.0085	1.14E–47

AT2G41370	20.39964308	2.545172565	3.0027	0.00030574
AT3G52740	32.10283968	4.006290149	3.0024	2.55E–06
AT4G33610	73.31844508	9.238033754	2.9885	1.07E–13
AT1G66800	32.88922047	4.147688624	2.9872	1.97E–06
AT1G70850	2617.075299	332.2864182	2.9775	0
AT2G32990	74.84494898	9.520830706	2.9747	6.37E–14
AT5G61420	31.77903582	4.053422974	2.9709	3.28E–06
AT4G04850	20.58467386	2.639438216	2.9633	0.00031162
AT4G37470	53.52015202	6.881392491	2.9593	4.81E–10
AT3G26210	25.99682406	3.346430595	2.9576	3.58E–05
AT4G36610	35.75719751	4.619016877	2.9526	6.96E–07
AT5G48800	15.68135829	2.026711487	2.9518	0.0022706
AT3G09925	16.74528525	2.168109963	2.9492	0.0015004
AT5G06630	45.61008634	5.938735985	2.9411	1.35E–08
AT5G49270	18.36430454	2.403774089	2.9335	0.00081535
AT1G67195	24.79412401	3.252164944	2.9305	6.32E–05

AT5G64940	34.73952824	4.571884052	2.9257	1.18E–06
AT5G50100	21.41731235	2.827969517	2.9209	0.0002484
AT3G21560	130.4929549	17.3448797	2.9114	1.47E–23
AT4G00700	38.62517454	5.184610781	2.8972	2.83E–07
AT1G54540	19.56700459	2.639438216	2.8901	0.00056051
AT4G33960	15.68135829	2.120977138	2.8862	0.0025693
AT1G13300	108.4280349	14.75257431	2.8777	1.94E–19
AT5G24090	19.01191226	2.59230539	2.8746	0.00072006
AT4G14440	48.15425951	6.598595539	2.8674	7.41E–09
AT3G29250	101.4431231	13.90418346	2.8671	3.80E–18
AT1G54010	43.66726319	5.98586881	2.8669	4.46E–08
AT1G10470	122.3516008	16.82641862	2.8622	8.82E–22
AT4G12030	27.75461643	3.817758848	2.8619	2.43E–05
AT1G23010	36.63609369	5.043212305	2.8609	7.33E–07
AT1G18870	18.13301607	2.49803974	2.8598	0.0010474
AT3G20100	56.34187136	7.776916171	2.8569	3.09E–10

AT1G07610	285.2249416	39.40304193	2.8557	2.96E–50
AT3G55120	235.4979204	32.56878227	2.8542	1.62E–41
AT2G19970	17.71669682	2.450906914	2.8537	0.0012502
AT5G13630	44.08358244	6.127267286	2.8469	4.22E–08
AT1G42550	36.86738216	5.137477955	2.8432	7.27E–07
AT3G62270	90.98888421	12.72586283	2.8379	3.59E–16
AT4G30450	60.92138307	8.672439851	2.8124	7.01E–11
AT3G50740	121.2876738	17.39201253	2.8019	3.49E–21
AT2G40320	16.328966	2.356641264	2.7926	0.0024066
AT1G13970	16.97657372	2.450906914	2.7922	0.0018884
AT5G07475	29.37363573	4.241954275	2.7917	1.68E–05
AT5G53370	96.30851903	13.95131628	2.7873	8.12E–17
AT3G16450	785.7331912	113.825773	2.7872	3.30E–135
AT4G20390	88.16716487	12.77299565	2.7871	1.98E–15
AT2G28250	69.57157186	10.08642461	2.7861	2.93E–12
AT3G44310	191.0905341	27.71410126	2.7856	5.44E–33

AT5G08050	18.50307762	2.686571041	2.7839	0.0010799
AT1G07590	567.6281638	82.48244424	2.7828	1.45E–97
AT2G47360	15.49632751	2.262375613	2.776	0.003404
AT3G51820	22.20369315	3.252164944	2.7713	0.00027407
AT5G45380	52.59499814	7.824048996	2.7489	2.82E–09
AT4G19690	134.0547974	19.98431792	2.7459	5.97E–23
AT2G48140	72.85586814	10.88768264	2.7423	1.23E–12
AT1G80240	57.54457141	8.672439851	2.7302	4.90E–10
AT1G78120	58.60849837	8.860971152	2.7256	3.39E–10
AT4G19030	39.87413228	6.033001636	2.7245	4.12E–07
AT4G37070	64.43696782	9.756494833	2.7235	3.71E–11
AT5G14200	157.9237675	23.94347524	2.7215	9.60E–27
AT1G18290	14.98749287	2.309508439	2.6981	0.0048274
AT1G79410	22.62001239	3.487829071	2.6972	0.00029151
AT4G01440	37.23744372	5.750204684	2.6951	1.29E–06
AT1G07090	21.0009931	3.252164944	2.691	0.00054055

AT5G42590	17.85546991	2.780836691	2.6828	0.0017402
AT4G25090	29.88247036	4.666149703	2.679	2.13E–05
AT1G75500	255.2036981	40.01576866	2.673	2.63E–42
AT4G21740	20.12209691	3.157899294	2.6717	0.0007835
AT1G31050	45.51757095	7.164189442	2.6675	6.92E–08
AT5G13580	46.53524022	7.399853569	2.6528	5.22E–08
AT4G11190	37.3299591	5.938735985	2.6521	1.54E–06
AT2G16750	91.63649193	14.70544149	2.6396	3.08E–15
AT5G05270	305.3007808	49.01813829	2.6388	5.99E–50
AT4G13770	81.13599537	13.0557926	2.6357	1.65E–13
AT3G59370	58.46972529	9.426565056	2.6329	7.38E–10
AT2G22510	125.3583509	20.21998204	2.6322	1.22E–20
AT5G01840	17.53166605	2.827969517	2.6321	0.0021941
AT1G73650	54.90788284	8.860971152	2.6315	2.75E–09
AT1G02810	117.4945429	18.99452859	2.6289	2.36E–19
AT2G01900	18.04050068	2.922235167	2.6261	0.0018599

AT1G69310	63.92813319	10.36922156		2.6241	1.06E–10
AT1G58360	28.44848184	4.619016877		2.6227	4.49E–05
AT4G20820	51.06849424	8.295377249		2.6221	1.20E–08
AT2G37970	196.5026844	31.95605554		2.6204	5.06E–32
AT3G54590	140.9471938	23.04795156		2.6124	5.30E–23
AT5G57150	60.7363523	9.992158959		2.6037	4.10E–10
AT5G25460	141.82609	23.37788134		2.6009	4.81E–23
AT2G40480	55.97180981	9.238033754		2.599	2.38E–09
AT1G12110	190.1653803	31.67325859		2.5859	1.32E–30
AT1G72430	46.07266328	7.68265052		2.5842	9.52E–08
AT1G79620	21.13976618	3.534961896		2.5802	0.00070045
AT2G21100	16.328966	2.733703866		2.5785	0.0037988
AT2G23610	77.8516991	13.0557926		2.576	1.05E–12
AT4G40090	33.12050894	5.561673383		2.5741	1.03E–05
AT3G26300	24.33154707	4.100555799		2.5689	0.00023444
AT3G26440	82.75501467	13.95131628		2.5684	1.95E–13

AT5G42500	32.01032429	5.420274907	2.5621	1.62E–05
AT2G34080	20.2146123	3.440696245	2.5546	0.0010404
AT5G24930	57.03573677	9.709362007	2.5544	2.30E–09
AT4G00080	23.45265089	4.006290149	2.5494	0.00034114
AT4G18640	44.3611286	7.58838487	2.5474	2.19E–07
AT4G25820	33.86063205	5.797337509	2.5461	9.03E–06
AT3G54600	35.20210518	6.033001636	2.5447	5.67E–06
AT3G44326	30.66885116	5.278876431	2.5385	2.85E–05
AT5G25810	56.34187136	9.709362007	2.5368	3.40E–09
AT2G15620	205.6154501	35.44388461	2.5363	1.96E–32
AT1G74460	104.5886463	18.05187208	2.5345	1.16E–16
AT1G72200	19.33571612	3.346430595	2.5306	0.0015061
AT3G32980	991.3023836	172.0348123	2.5266	4.79E–155
AT1G13930	1169.625794	203.4724067	2.5231	1.15E–182
AT5G53250	87.42704177	15.27103539	2.5173	6.75E–14
AT5G51110	19.89080844	3.487829071	2.5117	0.0013094

AT3G58810	27.93964721	4.901813829	2.5109	8.29E–05
AT2G17500	226.1538662	39.73297171	2.5089	2.91E–35
AT1G77760	72.90212583	12.82012848	2.5076	1.27E–11
AT5G23010	61.84653695	10.88768264	2.506	6.34E–10
AT5G57625	25.30295865	4.477618401	2.4985	0.0002132
AT5G23190	43.5747478	7.776916171	2.4862	4.20E–07
AT3G20110	34.55449746	6.174400111	2.4845	9.54E–06
AT2G21960	20.44590077	3.676360372	2.4755	0.0011998
AT5G67400	25.90430867	4.666149703	2.4729	0.00019041
AT3G60550	34.2306936	6.174400111	2.4709	1.14E–05
AT5G10130	33.16676664	5.98586881	2.4701	1.64E–05
AT3G22620	150.1062172	27.10137453	2.4695	3.79E–23
AT5G58770	21.37105465	3.864891673	2.4672	0.00090014
AT4G30470	82.80127236	14.98823844	2.4658	6.36E–13
AT3G24300	98.8526922	17.91047361	2.4645	2.41E–15
AT4G25640	406.9751923	73.81000439	2.4631	4.01E–62

AT4G18020	27.2457818	4.948946654	2.4608	0.00012677
AT3G21260	46.85904408	8.531041375	2.4575	1.65E–07
AT4G37750	19.89080844	3.629227546	2.4544	0.0015308
AT1G48500	31.22394349	5.703071859	2.4528	3.41E–05
AT4G13620	19.05816995	3.487829071	2.45	0.0020413
AT3G43670	49.35695956	9.049502453	2.4473	7.54E–08
AT5G66040	341.5668129	62.63952479	2.447	8.41E–52
AT1G49500	82.98630314	15.27103539	2.4421	7.95E–13
AT3G19030	493.5233379	91.06061844	2.4382	1.62E–74
AT4G15765	18.13301607	3.346430595	2.4379	0.0028661
AT2G22590	42.788367	7.918314647	2.434	7.54E–07
AT2G26355	79.42446069	14.70544149	2.4332	2.98E–12
AT2G47540	22.06492006	4.100555799	2.4279	0.0008062
AT1G10960	33.4443128	6.221532937	2.4264	1.83E–05
AT2G26980	86.82569174	16.16655907	2.4251	2.64E–13
AT4G11320	982.5134218	183.2524247	2.4226	3.22E–147

AT4G02075	17.9017276	3.346430595	2.4194	0.0032352
AT5G38030	71.37562193	13.38572238	2.4147	5.70E–11
AT1G49430	40.05916305	7.541252044	2.4093	2.20E–06
AT2G30840	24.28528938	4.571884052	2.4092	0.00040925
AT3G16420	2750.575004	517.942617	2.4089	0
AT3G04110	16.88405833	3.205032119	2.3973	0.0048104
AT5G65380	234.2952204	44.68191836	2.3906	9.23E–35
AT4G02270	32.24161276	6.174400111	2.3846	3.31E–05
AT4G24130	94.45821126	18.09900491	2.3838	3.45E–14
AT4G22160	18.36430454	3.534961896	2.3771	0.0031007
AT1G80050	44.77744785	8.625307026	2.3761	5.63E–07
AT4G27654	77.94421448	15.03537126	2.3741	9.79E–12
AT4G29020	26.36688561	5.09034513	2.3729	0.00023641
AT1G30650	34.64701285	6.692861189	2.372	1.62E–05
AT4G32650	87.79710332	16.9678171	2.3714	3.78E–13
AT2G23600	31.68652043	6.127267286	2.3706	4.26E–05

AT5G26280	913.4506845	176.7480948	2.3696	8.43E–134
AT3G63540	26.22811253	5.09034513	2.3653	0.0002548
AT5G38020	100.1016499	19.51298967	2.359	7.39E–15
AT1G72230	30.94639732	6.033001636	2.3588	5.72E–05
AT4G30670	47.4603941	9.28516658	2.3537	2.72E–07
AT3G63520	131.233078	25.68738978	2.353	2.60E–19
AT1G65985	43.48223241	8.531041375	2.3496	1.02E–06
AT1G71960	28.58725493	5.608806208	2.3496	0.000127
AT2G41660	110.0933119	21.6339668	2.3474	3.26E–16
AT5G41140	66.74985252	13.15005825	2.3437	5.47E–10
AT2G25810	39.18026687	7.729783346	2.3416	4.33E–06
AT5G38010	26.87572025	5.326009256	2.3352	0.00023255
AT5G40890	206.1705424	40.91129234	2.3333	7.34E–30
AT5G60660	89.55489569	17.81620796	2.3296	3.68E–13
AT1G16060	23.40639319	4.713282528	2.3121	0.00076229
AT1G52060	35.34087826	7.164189442	2.3025	1.83E–05

AT4G30190	1440.557108	292.1292511	2.3019	1.75E–204
AT2G44230	22.75878548	4.619016877	2.3008	0.00097113
AT3G45410	27.75461643	5.655939033	2.2949	0.000207
AT4G29720	35.38713595	7.211322268	2.2949	1.88E–05
AT5G42860	46.95155947	9.567963531	2.2949	4.82E–07
AT5G21020	489.128857	99.78019111	2.2934	2.97E–69
AT5G24380	35.75719751	7.305587918	2.2912	1.71E–05
AT1G31070	84.79035321	17.3448797	2.2894	2.91E–12
AT3G45650	63.41929855	13.00865978	2.2854	2.80E–09
AT4G12880	142.0573784	29.17521885	2.2837	3.19E–20
AT2G32430	24.19277399	4.996079479	2.2757	0.00067731
AT1G64390	123.9243624	25.59312413	2.2756	1.28E–17
AT2G25530	31.45523196	6.504329888	2.2738	7.18E–05
AT5G64120	316.9577197	65.60889279	2.2723	1.74E–44
AT1G53090	22.75878548	4.713282528	2.2716	0.0010668
AT3G25110	51.3922981	10.65201851	2.2704	1.43E–07

AT1G52750	26.92197794	5.608806208	2.263	0.00030484
AT2G46430	23.96148552	4.996079479	2.2618	0.00076364
AT5G17050	111.0647234	23.33074851	2.2511	1.19E–15
AT5G22020	194.4210881	40.91129234	2.2486	4.33E–27
AT5G03760	55.97180981	11.78320632	2.248	4.11E–08
AT1G08990	20.07583922	4.241954275	2.2427	0.0026511
AT1G01580	48.89438262	10.36922156	2.2374	3.97E–07
AT5G44550	125.4046086	26.63004628	2.2355	1.72E–17
AT5G03570	23.03633164	4.901813829	2.2325	0.0011192
AT3G56940	23.91522783	5.09034513	2.2321	0.00086144
AT4G26320	49.7732788	10.60488569	2.2306	3.19E–07
AT3G18170	30.02124344	6.410064238	2.2276	0.00013708
AT1G74090	47.09033255	10.13355743	2.2163	8.01E–07
AT3G62040	68.36887182	14.75257431	2.2124	1.25E–09
AT5G26290	65.63966787	14.18698041	2.21	2.93E–09
AT2G28160	32.657932	7.069923792	2.2077	6.81E–05

AT1G50250	68.09132565	14.75257431	2.2065	1.44E–09
AT1G12380	21.09350849	4.571884052	2.2059	0.0021815
AT1G72160	190.4891841	41.38262059	2.2026	5.87E–26
AT3G45780	36.5435783	7.965447472	2.1978	2.22E–05
AT1G30840	57.86837526	12.63159717	2.1957	3.61E–08
AT2G41310	56.0180675	12.25453457	2.1926	6.47E–08
AT3G02020	33.90688974	7.446986394	2.1868	5.20E–05
AT1G06650	19.52074689	4.2890871	2.1863	0.0036932
AT1G02730	29.14234726	6.410064238	2.1847	0.0002157
AT5G23020	219.4927583	48.40541156	2.1809	1.52E–29
AT1G74930	84.92912629	18.80599729	2.1751	1.23E–11
AT2G17820	42.8346247	9.520830706	2.1696	4.01E–06
AT1G56010	51.06849424	11.40614372	2.1626	3.63E–07
AT3G23510	49.58824803	11.07621394	2.1625	5.64E–07
AT5G61480	38.81020531	8.672439851	2.1619	1.39E–05
AT5G46900	42.97339778	9.615096357	2.1601	4.10E–06

AT3G20015	36.5435783	8.201111598	2.1557	2.80E–05
AT5G48412	152.3265865	34.21843115	2.1543	2.46E–20
AT2G31790	35.20210518	7.918314647	2.1524	4.25E–05
AT3G16430	195.2074689	43.92779316	2.1518	6.35E–26
AT1G72180	26.59817408	5.98586881	2.1517	0.00052701
AT1G11460	27.84713182	6.268665762	2.1513	0.00036615
AT5G19940	22.94381625	5.184610781	2.1458	0.0015448
AT5G21950	20.76970463	4.713282528	2.1397	0.0029526
AT5G10280	31.54774735	7.164189442	2.1387	0.00013201
AT2G24580	58.70101376	13.33858955	2.1378	4.72E–08
AT1G21910	66.98114099	15.22390256	2.1374	4.11E–09
AT3G14940	301.183846	68.67252643	2.1328	1.92E–39
AT1G11450	27.84713182	6.362931413	2.1298	0.00039954
AT5G09530	321.5834891	73.57434026	2.1279	5.57E–42
AT4G13860	34.97081671	8.012580297	2.1258	5.24E–05
AT3G30875	40.52173999	9.28516658	2.1257	1.05E–05

AT1G30270	186.5572801	42.79660535	2.1241	1.94E–24
AT2G33250	24.37780477	5.608806208	2.1198	0.0011268
AT5G22940	23.3138778	5.373142082	2.1174	0.0015353
AT1G74470	56.8044483	13.10292543	2.1161	9.98E–08
AT3G57010	71.00556038	16.4022232	2.114	1.62E–09
AT4G31890	19.33571612	4.477618401	2.1105	0.0048844
AT1G28400	298.0383228	69.09672186	2.1088	1.53E–38
AT3G52370	47.73794027	11.07621394	2.1077	1.46E–06
AT3G22600	311.6843426	72.67881658	2.1005	4.08E–40
AT5G40730	54.12150205	12.63159717	2.0992	2.50E–07
AT1G18880	90.20250341	21.11550572	2.0949	7.73E–12
AT5G42250	68.97022184	16.16655907	2.093	3.69E–09
AT4G29800	24.51657785	5.750204684	2.0921	0.0012026
AT2G05440	3925.520433	920.7397418	2.092	0
AT3G52900	19.89080844	4.666149703	2.0918	0.0044083
AT5G66490	29.51240881	6.928525316	2.0907	0.0002955

AT5G60890	61.01389846	14.32837888	2.0903	3.75E–08
AT2G31585	44.54615938	10.46348721	2.0899	4.16E–06
AT3G11720	27.2457818	6.410064238	2.0876	0.00056973
AT3G50640	20.81596233	4.901813829	2.0863	0.0034374
AT1G65310	52.64125584	12.44306587	2.0809	4.42E–07
AT4G31730	32.47290123	7.68265052	2.0796	0.00013525
AT3G28050	46.99781716	11.12334677	2.079	2.24E–06
AT1G78060	95.79968439	22.71802178	2.0762	2.00E–12
AT3G45160	90.43379188	21.4454355	2.0762	9.43E–12
AT5G22740	188.5926187	44.72905119	2.076	4.42E–24
AT1G70370	95.01330359	22.57662331	2.0733	2.62E–12
AT1G07560	23.59142397	5.608806208	2.0725	0.0016689
AT1G80870	30.48382038	7.258455093	2.0703	0.00024659
AT1G53940	39.31903995	9.426565056	2.0604	2.20E–05
AT4G04810	28.30970876	6.78712684	2.0604	0.00047345
AT1G04280	77.0190606	18.47606751	2.0596	5.44E–10

AT5G24120	22.34246623	5.373142082	2.056	0.0024966
AT5G42825	50.37462883	12.16026892	2.0505	1.07E–06
AT5G67070	22.62001239	5.467407732	2.0487	0.0023729
AT1G73885	36.40480522	8.813838327	2.0463	5.41E–05
AT2G45750	45.51757095	11.02908112	2.0451	4.33E–06
AT4G12480	46.25769406	11.21761242	2.0439	3.56E–06
AT3G51950	192.5707804	46.8028955	2.0407	4.14E–24
AT3G54720	27.66210105	6.739994015	2.0371	0.00062702
AT4G24780	107.2253348	26.30011651	2.0275	1.73E–13
AT2G30210	66.51856405	16.35509037	2.024	1.54E–08
AT4G28410	22.01866237	5.420274907	2.0223	0.0030558
AT1G64590	42.23327467	10.41635439	2.0195	1.29E–05
AT4G23400	240.5862668	59.38735985	2.0183	1.16E–29
AT2G01830	92.05281117	22.76515461	2.0156	1.42E–11
AT5G07680	22.43498162	5.561673383	2.0122	0.0028323
AT2G18570	32.657932	8.106845948	2.0102	0.00018225

AT3G23470	41.2156054	10.27495591	2.0041	1.86E–05
AT4G36180	45.9338902	11.45327654	2.0038	5.21E–06
AT2G13820	60.45880613	15.12963691	1.9986	1.05E–07
AT2G31570	123.6468162	30.96626621	1.9975	3.03E–15
AT4G38620	58.28469451	14.61117584	1.996	1.93E–07
AT2G05380	123.36927	31.01339903	1.992	3.64E–15
AT1G22550	55.83303673	14.04558193	1.991	3.92E–07
AT5G12940	33.76811666	8.531041375	1.9849	0.00015436
AT5G43630	102.0444731	25.82878825	1.9821	1.52E–12
AT3G06125	34.97081671	8.860971152	1.9806	0.00011484
AT4G15480	28.26345107	7.164189442	1.9801	0.00068327
AT4G00370	40.56799769	10.32208874	1.9746	2.68E–05
AT3G19370	205.9392539	52.55310018	1.9704	9.67E–25
AT4G32460	28.0321626	7.164189442	1.9682	0.00076404
AT2G02130	421.7313967	107.8870371	1.9668	2.96E–50
AT5G59090	308.0299847	79.04174799	1.9624	1.10E–36

AT5G52882	148.0708787	38.4132526	1.9466	1.21E–17
AT4G17250	31.03891271	8.059713123	1.9453	0.00038711
AT5G40830	33.72185897	8.766705502	1.9436	0.00019423
AT5G17160	27.15326641	7.069923792	1.9414	0.0010771
AT3G22800	64.94580246	16.92068427	1.9404	5.62E–08
AT1G45130	105.9763771	27.61983561	1.94	1.08E–12
AT1G30870	32.98173586	8.625307026	1.935	0.00024608
AT3G45070	31.17768579	8.153978773	1.9349	0.00039217
AT3G16770	146.5906325	38.50751825	1.9286	2.76E–17
AT3G54770	29.5586665	7.776916171	1.9263	0.00062185
AT4G15390	185.215807	48.87673981	1.922	1.17E–21
AT4G27860	113.1925774	29.88221123	1.9214	2.23E–13
AT2G23340	45.56382865	12.06600327	1.9169	1.08E–05
AT1G19210	24.00774322	6.362931413	1.9157	0.0026627
AT3G15354	27.01449333	7.164189442	1.9149	0.0012508
AT3G20380	23.63768166	6.268665762	1.9149	0.0029316

AT3G44550	51.53107118	13.66851933		1.9146	2.35E–06
AT5G57685	129.1977395	34.3126968		1.9128	3.84E–15
AT1G21440	62.40162928	16.5907545		1.9112	1.47E–07
AT5G63410	25.6730202	6.834259665		1.9094	0.0017968
AT3G03520	89.87869955	23.94347524		1.9083	1.23E–10
AT3G48115	87.01072252	23.23648286		1.9048	2.74E–10
AT1G22065	40.29045152	10.79341699		1.9003	4.65E–05
AT4G27657	39.55032842	10.65201851		1.8926	5.89E–05
AT3G19450	400.6378882	108.2640997		1.8877	1.64E–45
AT1G30530	24.37780477	6.598595539		1.8853	0.0027252
AT3G55990	22.38872392	6.080134461		1.8806	0.0045566
AT4G16190	410.8608386	112.0347257		1.8747	2.80E–46
AT1G09090	31.45523196	8.578174201		1.8746	0.00049391
AT2G05920	417.151885	113.9200387		1.8726	6.42E–47
AT2G45470	116.2455852	31.81465706		1.8694	2.65E–13
AT1G19715	24.79412401	6.78712684		1.8691	0.0026142

AT4G02970	63.51181394	17.39201253	1.8686	1.70E–07
AT4G39350	235.4979204	64.57197063	1.8667	1.64E–26
AT5G08330	35.10958979	9.662229182	1.8614	0.00021344
AT1G08320	58.00714835	15.97802777	1.8601	7.27E–07
AT3G26520	1087.102068	299.4819718	1.8599	4.08E–121
AT5G61820	213.8955773	58.96316442	1.859	5.28E–24
AT1G35260	42.88088239	11.83033914	1.8578	3.20E–05
AT4G22120	62.2628562	17.20348123	1.8557	2.64E–07
AT1G13420	34.41572438	9.520830706	1.8539	0.00026403
AT5G42030	26.22811253	7.258455093	1.8534	0.0019643
AT4G08930	37.97756682	10.51062004	1.8533	0.00011083
AT3G56980	26.87572025	7.446986394	1.8516	0.001689
AT4G36640	38.76394762	10.74628416	1.8509	9.29E–05
AT3G61270	41.2156054	11.45327654	1.8474	5.20E–05
AT2G27840	60.41254844	16.82641862	1.8441	4.68E–07
AT3G26330	41.72444004	11.64180784	1.8416	4.77E–05

AT2G19130	31.40897426	8.766705502	1.8411	0.00059156
AT1G55320	68.87770645	19.27732554	1.8371	6.27E–08
AT5G55730	26.08933945	7.305587918	1.8364	0.0021746
AT2G37180	135.3500128	37.98905717	1.833	4.46E–15
AT1G14480	60.27377536	16.9678171	1.8287	5.63E–07
AT5G44030	37.83879374	10.65201851	1.8287	0.00013282
AT1G12370	24.9328971	7.022790966	1.8279	0.0029699
AT3G06035	54.30653282	15.36530104	1.8214	2.59E–06
AT1G59970	28.30970876	8.012580297	1.821	0.0013699
AT5G10170	83.72642624	23.75494394	1.8175	2.08E–09
AT2G41380	39.82787458	11.31187807	1.8159	8.94E–05
AT2G42760	147.3307556	41.85394885	1.8156	3.45E–16
AT4G37540	80.62716074	22.90655309	1.8155	4.53E–09
AT1G75820	54.53782129	15.50669952	1.8144	2.60E–06
AT1G69252	79.28568761	22.62375613	1.8092	6.84E–09
AT5G11790	140.0682976	39.96863584	1.8092	2.39E–15

AT1G30510	151.956525	43.36219926	1.8091	1.30E–16
AT1G22500	49.95830958	14.28124606	1.8066	8.44E–06
AT5G24030	45.98014789	13.15005825	1.8059	2.18E–05
AT3G23530	110.2783426	31.62612576	1.802	4.11E–12
AT1G11545	40.42922461	11.59467502	1.8019	8.48E–05
AT2G02680	40.75302846	11.68894067	1.8018	7.87E–05
AT5G26260	437.7828165	125.986042	1.797	7.46E–47
AT4G01450	95.79968439	27.57270279	1.7968	1.51E–10
AT5G66052	55.97180981	16.11942625	1.7959	2.18E–06
AT1G60390	39.41155534	11.35901089	1.7948	0.00011258
AT3G57040	25.99682406	7.494119219	1.7945	0.002646
AT1G65510	41.77069773	12.06600327	1.7915	6.59E–05
AT2G38170	30.16001652	8.719572676	1.7903	0.001019
AT5G24290	48.52432107	14.04558193	1.7886	1.37E–05
AT1G49860	76.00139133	22.0110294	1.7878	2.00E–08
AT1G66200	1012.765954	293.3075717	1.7878	1.40E–107

AT4G04830	391.4326071	113.4958433	1.7861	1.30E–41
AT4G40060	137.1540629	39.96863584	1.7789	9.73E–15
AT5G16590	61.61524848	17.95760643	1.7787	6.66E–07
AT1G73330	1034.322039	301.5558161	1.7782	3.93E–109
AT1G54000	417.4294312	121.8854862	1.776	4.62E–44
AT3G50060	67.30494485	19.65438814	1.7759	1.80E–07
AT5G15910	45.28628248	13.2443239	1.7737	3.25E–05
AT1G68710	29.32737803	8.578174201	1.7735	0.0013409
AT5G24850	35.24836287	10.32208874	1.7718	0.00034248
AT5G46790	41.95572851	12.3016674	1.77	7.31E–05
AT3G27170	51.3460404	15.08250409	1.7674	8.37E–06
AT2G36380	396.5209535	116.5123441	1.7669	1.33E–41
AT5G56530	41.86321312	12.3016674	1.7668	7.64E–05
AT5G65390	80.9509646	23.94347524	1.7574	9.04E–09
AT3G54810	220.3253968	65.32609584	1.7539	4.75E–23
AT4G23510	26.36688561	7.824048996	1.7527	0.0028888

AT1G51850	45.42505556	13.52712085	1.7476	3.81E–05
AT5G60450	42.23327467	12.58446435	1.7467	8.05E–05
AT2G39890	38.76394762	11.59467502	1.7413	0.00018277
AT4G38080	279.9978221	84.03782747	1.7363	7.62E–29
AT1G77690	139.9757822	42.04248015	1.7353	1.36E–14
AT2G27000	45.65634403	13.71565216	1.735	3.98E–05
AT1G01750	26.50565869	7.965447472	1.7345	0.0030207
AT2G44940	42.51082084	12.82012848	1.7294	8.49E–05
AT4G14130	110.1395695	33.22864182	1.7288	1.59E–11
AT4G30460	139.4206899	42.1367458	1.7263	1.90E–14
AT4G09750	41.16934771	12.44306587	1.7262	0.0001169
AT3G11410	69.94163341	21.16263855	1.7246	1.73E–07
AT1G78570	507.8169653	154.31287	1.7185	1.98E–51
AT2G28550	96.4935498	29.36375015	1.7164	4.56E–10
AT1G47670	62.58666006	19.08879424	1.7131	1.04E–06
AT1G01610	94.45821126	28.84528907	1.7113	7.85E–10

AT4G22130	84.37403396	25.9230539	1.7026	8.65E–09
AT1G64640	37.00615525	11.40614372	1.698	0.00035382
AT5G66280	48.89438262	15.08250409	1.6968	2.58E–05
AT1G78100	58.14592143	17.95760643	1.6951	3.35E–06
AT5G44110	88.35219565	27.38417149	1.6899	4.25E–09
AT1G04220	56.11058289	17.39201253	1.6898	5.56E–06
AT3G57540	28.49473954	8.860971152	1.6852	0.0024193
AT5G06390	94.73575743	29.55228145	1.6806	1.19E–09
AT3G51860	47.22910563	14.75257431	1.6787	4.29E–05
AT4G14760	80.76593382	25.26319435	1.6767	2.79E–08
AT2G36830	698.0286033	218.7905749	1.6737	2.13E–68
AT2G42320	30.06750114	9.426565056	1.6734	0.0018259
AT3G12610	39.6891015	12.44306587	1.6734	0.00022869
AT5G24165	85.80802247	26.91284323	1.6728	9.53E–09
AT2G46535	54.81536746	17.20348123	1.6719	8.70E–06
AT5G39850	34.87830132	10.98194829	1.6672	0.00067291

AT4G34620	107.6879118	33.9356342	1.666	8.59E–11
AT2G23540	160.8380022	50.71492	1.6651	6.80E–16
AT5G10430	163.3359177	51.75184216	1.6582	4.72E–16
AT4G00430	92.05281117	29.17521885	1.6577	3.04E–09
AT4G27595	26.82946255	8.531041375	1.653	0.0040044
AT1G24280	46.76652869	14.89397279	1.6507	5.87E–05
AT1G63000	211.8602388	67.54133862	1.6493	1.43E–20
AT3G14680	79.47071839	25.35746	1.648	5.35E–08
AT3G23800	43.85229397	13.99844911	1.6474	0.00011171
AT2G14878	182.5791184	58.35043769	1.6457	9.86E–18
AT5G63800	41.53940926	13.29145673	1.644	0.00018676
AT5G63760	37.09867063	11.87747197	1.6431	0.0004811
AT1G14210	65.31586401	20.92697442	1.6421	1.19E–06
AT2G36570	42.92714008	13.76278498	1.6411	0.00014171
AT3G19680	137.0152898	43.97492598	1.6396	2.42E–13
AT3G63200	137.0615475	44.02205881	1.6385	2.45E–13

AT5G58860	98.39011526	31.86178989	1.6267	1.26E–09
AT1G77530	73.45721816	23.80207677	1.6258	2.55E–07
AT1G78580	39.87413228	12.96152695	1.6212	0.00030747
AT3G28200	47.09033255	15.36530104	1.6158	7.17E–05
AT2G22230	27.15326641	8.860971152	1.6156	0.0044006
AT1G15210	299.1485075	97.75347963	1.6136	3.91E–28
AT1G77640	31.1314281	10.18069026	1.6125	0.0019679
AT3G56240	213.340485	69.80371424	1.6118	3.89E–20
AT1G16720	38.99523609	12.77299565	1.6102	0.00039546
AT2G48030	43.99106705	14.42264454	1.6089	0.00014239
AT4G37410	34.92455901	11.45327654	1.6085	0.00092706
AT1G31770	64.48322551	21.16263855	1.6074	2.07E–06
AT1G65690	31.59400504	10.36922156	1.6073	0.001837
AT1G70690	31.50148965	10.36922156	1.6031	0.0019125
AT1G47530	229.0218433	75.41252044	1.6026	1.97E–21
AT4G21960	1726.105854	568.7518026	1.6016	7.66E–160

AT4G12390	55.92555211	18.42893468	1.6015	1.30E–05
AT5G36160	43.75977858	14.42264454	1.6013	0.0001578
AT1G07870	52.68751353	17.39201253	1.599	2.57E–05
AT4G34138	180.2662337	59.57589115	1.5973	7.03E–17
AT5G44130	46.85904408	15.50669952	1.5954	8.78E–05
AT3G15950	1562.261101	518.5082109	1.5912	1.61E–143
AT1G05710	33.02799356	10.98194829	1.5886	0.001517
AT5G02480	165.7413178	55.14540558	1.5876	1.95E–15
AT5G65970	68.5076449	22.81228743	1.5865	1.13E–06
AT5G23920	28.7722857	9.615096357	1.5813	0.0036862
AT5G12420	101.9982154	34.1241655	1.5797	1.29E–09
AT5G44350	37.46873219	12.53733152	1.5795	0.00065409
AT3G16410	42.92714008	14.37551171	1.5783	0.00021936
AT3G12520	30.06750114	10.08642461	1.5758	0.0029205
AT4G00040	52.64125584	17.67480948	1.5745	3.20E–05
AT3G54580	422.0089429	141.7755384	1.5737	2.46E–38

AT1G04250	111.5273004	37.51772892	1.5718	2.12E–10
AT2G14890	287.5840839	96.7636903	1.5714	3.61E–26
AT1G76790	120.4550353	40.53422974	1.5713	3.42E–11
AT1G75780	60.78260999	20.45564617	1.5712	6.40E–06
AT5G53460	1418.214642	477.6911842	1.5699	4.07E–128
AT1G36060	34.41572438	11.59467502	1.5696	0.001275
AT3G23640	110.7871773	37.37633045	1.5676	2.66E–10
AT1G53300	74.61366051	25.21606152	1.5651	4.19E–07
AT2G36890	28.91105879	9.803627658	1.5602	0.0039654
AT4G36190	28.7722857	9.756494833	1.5602	0.0040725
AT1G32640	514.0154964	174.532852	1.5583	4.64E–46
AT2G04780	96.72483827	32.85157922	1.5579	5.35E–09
AT1G12040	38.39388607	13.0557926	1.5562	0.00062836
AT3G55420	36.40480522	12.39593305	1.5543	0.00094136
AT2G40460	33.07425125	11.26474524	1.5539	0.0018077
AT4G20270	85.90053786	29.2694845	1.5533	5.11E–08

AT1G58235	29.00357417	9.897893308	1.551	0.0040651
AT1G05570	316.1250812	107.9341699	1.5503	2.97E–28
AT2G42840	41.40063618	14.23411323	1.5403	0.00038606
AT4G34260	46.62775561	16.07229342	1.5366	0.00014275
AT1G30400	367.9799562	127.2586283	1.5319	2.46E–32
AT4G13290	38.53265915	13.33858955	1.5305	0.00071714
AT2G40150	32.93547817	11.40614372	1.5298	0.0021038
AT1G80830	173.3275796	60.0472194	1.5293	2.16E–15
AT3G21190	58.14592143	20.17284922	1.5273	1.66E–05
AT3G16400	1096.122318	380.9746267	1.5246	2.14E–95
AT2G01890	69.89537572	24.32053784	1.523	1.76E–06
AT1G80280	31.36271657	10.93481546	1.5201	0.0029825
AT1G61667	28.91105879	10.08642461	1.5192	0.0048169
AT5G05960	70.91304499	24.74473327	1.5189	1.51E–06
AT3G63120	60.64383691	21.16263855	1.5188	1.11E–05
AT5G57350	51.57732887	18.00473926	1.5184	6.37E–05

AT3G27060	35.06333209	12.25453457	1.5166	0.0015123
AT3G54040	142.5199554	49.81939632	1.5164	1.35E–12
AT3G14350	105.6988309	36.99926784	1.5144	1.88E–09
AT2G41820	32.01032429	11.21761242	1.5128	0.0027454
AT5G08240	142.3811823	49.96079479	1.5109	1.58E–12
AT5G03555	35.71093981	12.53733152	1.5101	0.0013857
AT3G06390	45.74885942	16.07229342	1.5092	0.00020794
AT1G64370	135.9976205	47.83981766	1.5073	6.00E–12
AT4G25630	160.3291676	56.41799186	1.5068	5.34E–14
AT2G47460	80.67341843	28.42109364	1.5051	2.73E–07
AT2G16720	39.59658611	13.95131628	1.505	0.00068784
AT5G17820	147.5157863	52.17603758	1.4994	7.73E–13
AT3G17609	37.00615525	13.10292543	1.4979	0.0011699
AT2G36290	230.5020895	81.91685033	1.4925	9.95E–20
AT1G33800	65.08457554	23.14221721	1.4918	6.27E–06
AT3G46700	138.4030206	49.34806807	1.4878	5.89E–12

AT1G25520	35.52590904	12.67873	1.4865	0.001641
AT3G26770	36.31228983	13.00865978	1.481	0.0014686
AT5G27420	66.70359483	23.99060807	1.4753	5.54E–06
AT1G30410	36.03474367	12.96152695	1.4752	0.0015944
AT3G45310	77.57415293	27.90263256	1.4752	7.22E–07
AT1G75270	138.495536	49.96079479	1.471	8.53E–12
AT1G06000	60.41254844	21.86963093	1.4659	1.94E–05
AT1G54030	103.1084001	37.32919762	1.4658	7.13E–09
AT4G26220	48.2467749	17.48627818	1.4642	0.00018384
AT3G27390	72.48580659	26.30011651	1.4626	2.18E–06
AT4G26130	52.45622506	19.08879424	1.4584	8.98E–05
AT1G50750	50.46714422	18.38180186	1.4571	0.00013019
AT1G29025	37.60750527	13.71565216	1.4552	0.0013531
AT2G22330	181.6539646	66.26875234	1.4548	3.92E–15
AT2G10410	242.9454092	88.79824282	1.452	4.67E–20
AT2G30520	140.3458438	51.37477955	1.4499	9.83E–12

AT4G02290	56.98947908	20.92697442	1.4453	4.44E–05
AT1G28580	162.9195985	59.8586881	1.4445	1.75E–13
AT1G67360	81.6910877	30.0236097	1.4441	5.09E–07
AT5G10480	87.9358764	32.38025097	1.4413	1.71E–07
AT4G12470	63.28052547	23.37788134	1.4366	1.56E–05
AT1G20560	47.04407486	17.39201253	1.4356	0.00028487
AT2G37170	655.5177825	242.4983861	1.4347	9.18E–53
AT3G05490	131.4643665	48.68820851	1.433	7.32E–11
AT5G55550	33.95314744	12.58446435	1.4319	0.0029526
AT5G07030	142.3349246	52.78876431	1.431	1.06E–11
AT1G78340	56.98947908	21.16263855	1.4292	5.15E–05
AT4G17880	35.10958979	13.0557926	1.4272	0.0024744
AT2G38760	33.81437436	12.58446435	1.426	0.0031177
AT3G21510	156.6285521	58.35043769	1.4245	9.33E–13
AT1G68560	114.3952774	42.65520688	1.4232	1.94E–09
AT2G30200	64.06690627	23.94347524	1.4199	1.62E–05

AT3G51350	32.61167431	12.20740175	1.4176	0.0040473
AT2G36420	89.32360722	33.46430595	1.4164	1.91E–07
AT3G01690	194.1897996	72.82021505	1.4151	1.38E–15
AT2G30970	92.00655348	34.54836093	1.4131	1.25E–07
AT5G61030	62.86420622	23.61354546	1.4126	2.14E–05
AT1G64330	92.70041889	34.92542353	1.4083	1.20E–07
AT1G20950	129.3365126	48.78247416	1.4067	1.90E–10
AT5G62440	54.4915636	20.59704465	1.4036	0.0001004
AT5G10470	52.03990581	19.74865379	1.3979	0.00016074
AT1G75750	584.4197067	221.9013414	1.3971	1.82E–45
AT1G41830	148.5797133	56.41799186	1.397	7.89E–12
AT4G01480	163.1046292	61.97966524	1.3959	6.26E–13
AT1G35320	50.37462883	19.18305989	1.3929	0.0002228
AT4G21910	63.51181394	24.22627219	1.3905	2.40E–05
AT1G27930	36.82112447	14.04558193	1.3904	0.0022784
AT4G31920	39.41155534	15.03537126	1.3903	0.0014747

AT3G53510	38.85646301	14.84683996	1.388	0.00164
AT3G02230	335.3220242	128.3426832	1.3855	6.32E–26
AT5G20960	422.1014583	161.7598564	1.3837	1.71E–32
AT5G59450	50.6059173	19.41872401	1.3819	0.00023422
AT4G31990	169.3956756	65.04329888	1.3809	3.15E–13
AT2G47800	108.3355195	41.61828472	1.3802	1.24E–08
AT4G14960	522.8969736	200.8801013	1.3802	4.62E–40
AT1G23030	54.53782129	20.97410725	1.3786	0.00012407
AT3G23090	89.69366878	34.5012281	1.3784	3.13E–07
AT3G52430	98.62140373	37.94192435	1.3781	6.85E–08
AT2G26650	189.5177725	72.96161353	1.3771	1.06E–14
AT2G47860	37.60750527	14.51691019	1.3733	0.0022094
AT4G13010	98.20508448	37.94192435	1.372	8.12E–08
AT1G63940	207.0494386	80.1729358	1.3688	6.73E–16
AT4G35040	67.44371793	26.15871803	1.3664	1.60E–05
AT5G45480	39.59658611	15.36530104	1.3657	0.0016664

AT1G31540	40.89180155	15.88376212	1.3643	0.001361
AT4G35300	70.31169497	27.33703866	1.3629	1.03E–05
AT5G37790	47.36787871	18.47606751	1.3583	0.00048675
AT5G13650	164.5386178	64.24204085	1.3568	1.40E–12
AT1G66180	281.6630991	110.0080142	1.3564	3.06E–21
AT5G16000	37.79253604	14.79970714	1.3525	0.0024283
AT5G24735	100.0553922	39.21451063	1.3513	8.30E–08
AT2G37860	47.36787871	18.57033316	1.3509	0.00051567
AT2G16400	41.35437849	16.2136919	1.3508	0.0013772
AT1G53280	95.66091131	37.51772892	1.3504	1.75E–07
AT5G41700	489.4526608	192.0191302	1.3499	1.92E–36
AT5G57740	37.42247449	14.70544149	1.3476	0.0026621
AT1G49580	58.28469451	22.90655309	1.3474	8.87E–05
AT3G55360	75.95513364	29.92934405	1.3436	5.05E–06
AT1G62990	38.48640145	15.17676974	1.3425	0.0023085
AT3G26590	37.97756682	14.98823844	1.3413	0.0025244

AT5G41670	223.6096931	88.79824282	1.3324	1.49E–16
AT1G67730	133.0833858	52.97729561	1.3289	5.40E–10
AT1G51070	166.2964101	66.26875234	1.3274	2.35E–12
AT1G22440	145.3879324	57.97337509	1.3264	7.53E–11
AT5G17010	88.39845334	35.25535331	1.3262	8.22E–07
AT4G15160	63.55807163	25.35746	1.3257	4.65E–05
AT2G40080	206.2630578	82.34104576	1.3248	3.43E–15
AT4G30440	329.6323278	131.6891138	1.3237	5.03E–24
AT1G74450	121.8427661	48.68820851	1.3234	3.80E–09
AT1G01630	63.18801008	25.26319435	1.3226	5.10E–05
AT4G15920	62.63291775	25.07466305	1.3207	5.68E–05
AT2G30860	1164.306159	466.7092359	1.3189	7.81E–84
AT1G09560	397.5386227	159.3560823	1.3188	9.19E–29
AT5G65310	167.5916256	67.21140885	1.3182	2.44E–12
AT3G59670	40.98431693	16.44935602	1.317	0.0018238
AT4G34135	92.33035734	37.23493197	1.3101	5.57E–07

AT3G05165	173.0500335	69.80371424	1.3098	1.27E–12
AT1G50560	51.16100963	20.64417747	1.3093	0.00038813
AT2G43360	59.67242533	24.08487372	1.3089	0.00010182
AT2G04160	37.28370141	15.08250409	1.3057	0.0034795
AT1G52280	40.75302846	16.49648885	1.3047	0.0020428
AT5G40910	61.75402157	25.02753022	1.303	7.81E–05
AT3G56370	45.56382865	18.47606751	1.3022	0.0009863
AT1G33560	45.65634403	18.52320033	1.3015	0.00097851
AT1G74950	151.9102673	61.74400111	1.2988	5.16E–11
AT2G34585	122.6291469	49.86652914	1.2982	5.46E–09
AT1G20510	179.3873376	72.96161353	1.2979	6.49E–13
AT1G04680	68.97022184	28.09116387	1.2959	2.70E–05
AT1G05000	66.84236791	27.24277301	1.2949	3.80E–05
AT2G24570	51.99364812	21.30403703	1.2872	0.00040833
AT1G77885	57.82211757	23.70781111	1.2863	0.00016784
AT5G10560	46.53524022	19.08879424	1.2856	0.00096039

AT5G40250	41.58566696	17.06208275	1.2853	0.0020413
AT2G18980	77.38912216	31.81465706	1.2824	8.61E–06
AT2G01950	72.25451812	29.74081275	1.2806	1.93E–05
AT2G43970	752.7514554	309.945459	1.2802	4.03E–52
AT5G44480	50.92972116	20.97410725	1.2799	0.00051305
AT1G74770	86.59440327	35.67954874	1.2792	2.15E–06
AT4G05150	190.6742149	78.57041974	1.2791	1.94E–13
AT4G39955	50.28211344	20.73844312	1.2777	0.00057646
AT2G34710	67.7212641	28.04403104	1.2719	4.28E–05
AT5G59780	86.68691866	35.91521286	1.2712	2.37E–06
AT3G18130	95.38336514	39.59157323	1.2685	6.47E–07
AT2G45510	65.26960631	27.10137453	1.268	6.45E–05
AT3G16470	322.2310968	133.810091	1.2679	3.37E–22
AT1G58080	74.10482588	30.82486773	1.2655	1.74E–05
AT1G18200	37.70002066	15.69523082	1.2642	0.0041971
AT3G58710	49.07941339	20.45564617	1.2626	0.00077723

AT4G36900	42.64959392	17.81620796	1.2593	0.0020696
AT1G28370	45.42505556	18.99452859	1.2579	0.0013857
AT1G30120	129.7990895	54.29701472	1.2573	4.18E–09
AT1G55210	83.68016855	35.01968918	1.2567	4.57E–06
AT2G26900	51.43855579	21.53970115	1.2559	0.00057801
AT2G21660	2014.106257	845.1386901	1.2529	2.18E–135
AT3G15820	72.06948734	30.25927383	1.252	2.76E–05
AT3G63210	71.42187962	30.0236097	1.2503	3.11E–05
AT1G21310	6010.262189	2527.874818	1.2495	0
AT5G56540	102.2757616	43.0794023	1.2474	3.21E–07
AT5G57070	60.36629074	25.45172565	1.246	0.00016733
AT2G46650	55.37045979	23.37788134	1.244	0.00035704
AT4G11650	79.65574916	33.65283725	1.2431	1.00E–05
AT3G52960	80.62716074	34.1241655	1.2405	8.98E–06
AT5G15740	42.23327467	17.91047361	1.2376	0.0025458
AT2G36910	137.1078052	58.16190639	1.2372	2.15E–09

AT4G17770	39.08775148	16.5907545	1.2363	0.0040616
AT2G04030	158.8951791	67.44707297	1.2362	8.53E–11
AT3G28860	151.4014326	64.28917368	1.2357	2.67E–10
AT2G47400	62.81794853	26.81857758	1.2279	0.00013941
AT3G16240	106.0688925	45.29464509	1.2276	2.55E–07
AT2G43290	93.90311893	40.11003431	1.2272	1.52E–06
AT5G19740	66.42604866	28.37396082	1.2272	8.39E–05
AT3G55010	44.03732474	18.85313011	1.2239	0.002152
AT3G22240	72.53206428	31.06053186	1.2235	3.59E–05
AT2G22770	84.46654935	36.24514264	1.2206	6.62E–06
AT3G46290	49.95830958	21.49256833	1.2169	0.00097915
AT4G29700	59.67242533	25.68738978	1.216	0.00024623
AT3G12110	40.61425538	17.48627818	1.2158	0.0037006
AT5G18860	89.13857645	38.46038543	1.2127	3.76E–06
AT1G04040	112.0823927	48.40541156	1.2113	1.42E–07
AT2G16890	51.16100963	22.10529506	1.2107	0.00086786

AT1G11840	462.6231983	199.890312	1.2106	7.90E–30
AT4G13340	648.1165514	280.1575135	1.21	1.21E–41
AT2G41475	70.45046805	30.49493795	1.208	5.80E–05
AT5G58350	47.32162102	20.502779	1.2067	0.0015316
AT2G30020	317.1427504	137.580717	1.2049	2.00E–20
AT4G23670	654.1300516	283.9281395	1.2041	9.52E–42
AT5G65640	53.65892511	23.37788134	1.1987	0.00067511
AT2G29750	46.39646714	20.26711487	1.1949	0.0019013
AT2G39700	110.0470542	48.07548178	1.1947	2.55E–07
AT5G44530	40.52173999	17.7219423	1.1932	0.0043452
AT1G15500	417.6144619	182.7339636	1.1924	2.06E–26
AT3G23490	77.43537985	33.88850137	1.1922	2.60E–05
AT3G13690	51.66984426	22.62375613	1.1915	0.0009449
AT2G43150	2533.071327	1111.674817	1.1882	1.72E–158
AT3G27230	57.40579832	25.21606152	1.1869	0.00044349
AT2G23030	43.38971702	19.08879424	1.1846	0.0030801

AT4G24240	49.95830958	22.0110294	1.1825	0.0012819
AT2G39130	47.83045565	21.11550572	1.1796	0.0017497
AT5G37990	141.5022861	62.54525914	1.1779	4.32E–09
AT1G56280	1149.226151	508.6103176	1.176	5.88E–71
AT4G30020	44.59241707	19.74865379	1.175	0.0028
AT4G17520	314.5060619	139.3246315	1.1746	1.37E–19
AT3G08030	81.27476846	36.10374416	1.1707	2.00E–05
AT1G53730	57.91463296	25.7345226	1.1702	0.00048186
AT2G43710	101.3968654	45.05898097	1.1701	1.28E–06
AT5G27950	58.09966373	25.82878825	1.1695	0.00047275
AT1G76090	118.0496352	52.60023301	1.1663	1.41E–07
AT5G64030	98.34385756	43.83352751	1.1658	2.10E–06
AT1G47480	51.57732887	23.00081874	1.1651	0.0011836
AT5G46470	61.80027926	27.57270279	1.1644	0.00030066
AT5G03240	2374.083632	1059.640178	1.1638	1.88E–144
AT3G55230	49.86579419	22.29382636	1.1614	0.0015278

AT5G60920	292.7649457	131.0763871	1.1593	6.12E–18
AT1G80270	43.34345933	19.41872401	1.1584	0.0037103
AT5G52020	195.2537266	87.80845349	1.1529	5.15E–12
AT3G27150	43.01965547	19.37159119	1.1511	0.0040784
AT1G77510	58.74727145	26.53578063	1.1466	0.00053694
AT5G49810	121.7965085	55.05113992	1.1456	1.26E–07
AT3G02630	72.43954889	32.75731357	1.145	8.98E–05
AT1G64650	82.5237262	37.37633045	1.1427	2.43E–05
AT4G29270	60.55132152	27.43130431	1.1423	0.00043906
AT4G29100	61.52273309	27.90263256	1.1407	0.00039217
AT1G53240	212.3228157	96.38662769	1.1394	7.98E–13
AT4G12600	94.41195357	42.890871	1.1383	5.42E–06
AT5G42180	43.34345933	19.70152097	1.1375	0.0042806
AT3G54400	62.81794853	28.56249212	1.1371	0.00034357
AT1G79270	136.9227744	62.26246219	1.1369	2.02E–08
AT4G02540	68.18384104	31.01339903	1.1365	0.0001714

AT4G07825	56.20309828	25.59312413	1.1349	0.00083015
AT3G04940	58.19217912	26.53578063	1.1329	0.00065216
AT4G21600	69.2940257	31.62612576	1.1316	0.00015661
AT5G26780	84.55906473	38.6017839	1.1313	2.16E–05
AT1G35720	1107.13165	505.6409496	1.1306	8.28E–65
AT2G04410	136.0901359	62.16819654	1.1303	2.61E–08
AT1G43710	146.4981171	66.97574472	1.1292	6.73E–09
AT3G15530	82.24618003	37.61199457	1.1288	3.02E–05
AT4G18010	257.4240674	117.7849304	1.128	2.91E–15
AT3G04810	57.96089065	26.53578063	1.1271	0.00070786
AT1G80490	133.0371281	60.94274308	1.1263	4.22E–08
AT3G44590	56.850706	26.06445238	1.1251	0.00083201
AT5G19530	73.73476433	33.88850137	1.1215	9.90E–05
AT3G27300	89.32360722	41.09982364	1.1199	1.37E–05
AT1G79040	74.42862974	34.26556398	1.1191	9.34E–05
AT4G35160	70.54298344	32.52164944	1.1171	0.00015631

AT1G64950	68.73893337	31.72039141	1.1157	0.00019929
AT2G41480	54.16775974	25.02753022	1.1139	0.0012847
AT1G35516	66.56482175	30.77773491	1.1129	0.00027023
AT3G25530	78.68433759	36.38654111	1.1127	5.87E–05
AT1G67030	50.92972116	23.56641264	1.1118	0.0019634
AT5G19240	111.3422696	51.56331085	1.1106	9.34E–07
AT2G42490	262.7437022	121.7912205	1.1092	3.16E–15
AT4G14010	124.1093932	57.54917966	1.1087	1.90E–07
AT4G20260	549.2638592	254.7057878	1.1087	2.09E–31
AT2G26730	64.43696782	29.88221123	1.1086	0.00037058
AT1G68810	54.4453059	25.31032717	1.1051	0.0013381
AT5G02050	64.34445243	29.92934405	1.1043	0.00039072
AT3G45600	97.04864213	45.15324662	1.1039	6.44E–06
AT2G39900	52.03990581	24.22627219	1.103	0.0018305
AT1G62800	54.95414054	25.59312413	1.1025	0.0012829
AT3G47620	56.66567522	26.39438216	1.1022	0.00104

AT5G50400	45.84137481	21.39830268	1.0992	0.0040682
AT4G18040	76.97280291	35.96234569	1.0979	8.71E–05
AT2G38120	135.6738167	63.53504847	1.0945	5.89E–08
AT1G45201	73.64224894	34.54836093	1.0919	0.00014015
AT3G23810	368.2112447	172.8360703	1.0911	1.03E–20
AT1G19600	92.83919197	43.59786338	1.0905	1.34E–05
AT5G44020	143.7226554	67.4942058	1.0905	2.37E–08
AT2G05940	158.2475714	74.37559829	1.0893	3.92E–09
AT2G38800	511.656354	240.7073387	1.0879	1.53E–28
AT5G04830	108.2430041	50.95058413	1.0871	2.08E–06
AT3G23600	260.6158483	122.6867442	1.0869	1.06E–14
AT1G64230	418.4008427	197.0623425	1.0862	2.37E–23
AT1G56110	258.9043136	122.1211503	1.0841	1.48E–14
AT2G35940	62.49414467	29.50514862	1.0828	0.0006087
AT3G54010	105.6988309	49.91366197	1.0825	3.08E–06
AT1G66760	97.92753832	46.28443442	1.0812	8.21E–06

AT5G64330	49.44947495	23.37788134	1.0808	0.0029807
AT3G56200	46.53524022	22.0110294	1.0801	0.0042758
AT2G05990	146.9144363	69.61518294	1.0775	2.14E–08
AT4G17340	161.8094138	76.68510673	1.0773	3.40E–09
AT5G01890	63.41929855	30.07074253	1.0766	0.00057765
AT3G13470	75.44629901	35.77381439	1.0765	0.00013452
AT3G18780	1299.656172	618.1941363	1.072	1.00E–70
AT5G06750	76.74151444	36.52793959	1.071	0.00012285
AT3G09980	91.45146115	43.69212903	1.0656	2.24E–05
AT4G39260	789.8038683	377.4396648	1.0652	1.18E–42
AT3G62130	52.78002892	25.26319435	1.063	0.0023075
AT5G14390	65.45463709	31.34332881	1.0623	0.00052114
AT1G48920	603.5241344	289.0184846	1.0622	1.59E–32
AT5G15350	178.2308952	85.35754658	1.0622	6.86E–10
AT2G06850	60.36629074	28.98668755	1.0584	0.00098585
AT5G20885	57.40579832	27.57270279	1.058	0.0013973

AT3G24080	174.2989912	83.84929617	1.0557	1.31E–09
AT5G37310	48.75560954	23.47214699	1.0546	0.0039594
AT1G04820	366.4071946	176.6066963	1.0529	1.19E–19
AT3G22850	146.6368902	70.84063639	1.0496	4.18E–08
AT1G58270	318.0216466	153.7001432	1.049	5.15E–17
AT4G26690	204.875327	99.12033156	1.0475	4.32E–11
AT3G54200	60.96764077	29.50514862	1.0471	0.001019
AT2G25520	210.9813426	102.1368324	1.0466	2.13E–11
AT1G06640	343.5096361	166.3317404	1.0463	2.75E–18
AT2G38080	49.72702111	24.08487372	1.0459	0.0037608
AT2G47180	52.1786789	25.31032717	1.0437	0.0028847
AT5G58750	68.4613872	33.22864182	1.0429	0.00044572
AT4G37870	460.0327674	223.4095918	1.042	3.10E–24
AT2G28570	82.5237262	40.11003431	1.0408	9.00E–05
AT1G28290	581.3204412	282.6084204	1.0405	1.76E–30
AT5G67470	58.14592143	28.32682799	1.0375	0.0015333

AT1G20620	502.2660421	244.7607617	1.0371	2.97E–26
AT5G56000	61.4764754	29.97647688	1.0362	0.0010632
AT4G26010	52.59499814	25.68738978	1.0339	0.0029715
AT2G18280	174.5765374	85.45181223	1.0307	2.50E–09
AT3G61200	59.62616764	29.2694845	1.0265	0.0014327
AT1G12140	49.63450572	24.36767067	1.0264	0.0044144
AT5G07580	61.24518693	30.07074253	1.0262	0.0011994
AT3G23560	50.18959805	24.65046762	1.0258	0.0041635
AT5G09870	169.5344487	83.28370227	1.0255	5.12E–09
AT3G01520	149.7361557	73.57434026	1.0251	5.10E–08
AT2G28890	59.07107531	29.03382037	1.0247	0.0015448
AT4G27652	206.540604	101.5712385	1.0239	7.54E–11
AT3G29360	181.0526145	89.08103978	1.0232	1.46E–09
AT4G16340	113.100062	55.71099948	1.0216	3.59E–06
AT3G24503	355.7216673	175.4755085	1.0195	2.84E–18
AT4G38920	291.0071533	143.5665858	1.0193	5.07E–15

AT5G48560	79.19317222	39.07311216	1.0192	0.00017119
AT5G46290	150.2449903	74.18706699	1.0181	5.65E–08
AT4G10480	189.8415764	93.84145513	1.0165	6.48E–10
AT2G41530	104.9587078	51.89324063	1.0162	9.91E–06
AT5G07340	59.9499715	29.6465471	1.0159	0.0015218
AT2G41220	339.9477936	168.1699206	1.0154	2.20E–17
AT3G21710	57.40579832	28.42109364	1.0142	0.0020413
AT5G45340	89.83244186	44.49338706	1.0136	5.62E–05
AT1G18660	60.7363523	30.11787535	1.0119	0.0014464
AT2G12400	130.8630165	64.94903323	1.0107	5.89E–07
AT5G60690	58.28469451	28.98668755	1.0077	0.0019643
AT1G75680	124.5257124	62.02679807	1.0055	1.32E–06
AT4G24830	79.56323378	39.63870606	1.0052	0.00019436
AT3G53420	738.0415087	367.7303028	1.0051	7.80E–37
AT4G18030	208.2058809	103.7864813	1.0044	1.17E–10
AT3G25860	129.7528318	64.71336911	1.0036	7.63E–07

AT5G52540	56.20309828	28.04403104	1.003	0.0025672
AT5G66730	53.75144049	107.5099745	–1.0001	0.0023915
AT3G49790	49.68076342	99.49739416	–1.002	0.0037103
AT1G56145	76.32519519	152.9931509	–1.0032	0.00016291
ATCG01130	123.6930739	248.2485907	–1.005	5.68E–07
AT1G04400	107.502881	216.0568711	–1.007	3.65E–06
AT1G07820	70.49672574	141.6812728	–1.007	0.00029635
AT4G01000	134.7949205	270.9194797	–1.0071	1.39E–07
AT3G19910	67.11991408	134.9884116	–1.008	0.00043219
AT2G30040	50.00456727	100.628582	–1.0089	0.0031957
AT4G21570	107.6879118	216.8581291	–1.0099	3.24E–06
AT4G03390	48.75560954	98.46047201	–1.014	0.0034218
AT3G07780	167.0827909	337.7538259	–1.0154	1.81E–09
AT1G53430	56.29561367	113.9671715	–1.0175	0.0013102
AT1G33980	57.91463296	117.8320632	–1.0247	0.00094217
AT3G07090	61.52273309	125.1847839	–1.0249	0.00060206

AT5G57560	403.8759268	822.0436057	–1.0253	7.16E–23
AT5G49520	69.47905647	141.7755384	–1.029	0.0002037
AT2G14170	203.0250192	414.5803311	–1.03	7.88E–12
AT5G18130	61.15267154	124.9491198	–1.0309	0.00055907
AT3G10985	184.013107	376.4498755	–1.0326	7.69E–11
AT2G26530	144.9253555	296.7954008	–1.0342	1.12E–08
AT4G21580	67.02739869	137.4864513	–1.0365	0.00023408
AT1G67310	69.47905647	142.8124606	–1.0395	0.00015946
AT4G39640	47.73794027	98.46047201	–1.0444	0.0024038
AT5G44070	67.6750064	139.6074285	–1.0447	0.00017877
AT3G59350	134.1935705	277.0938798	–1.0461	2.61E–08
AT5G42570	85.34544553	176.3238994	–1.0468	1.66E–05
AT2G48020	45.1475094	93.32299405	–1.0476	0.0031957
AT5G44290	100.7492577	208.4684862	–1.0491	1.99E–06
AT5G28770	57.26702524	118.6333212	–1.0507	0.00061985
AT5G21940	303.8205346	629.5060144	–1.051	1.81E–18

AT5G53310	48.43180568	100.3929178	–1.0516	0.0019554
AT2G38470	285.4099723	591.846887	–1.0522	2.02E–17
AT1G62380	156.0734597	323.8025097	–1.0529	9.66E–10
AT3G27260	80.1645838	166.6616702	–1.0559	2.56E–05
AT4G27320	81.32102615	169.2068427	–1.0571	2.11E–05
AT3G63310	58.88604453	122.9695412	–1.0623	0.00039358
AT1G22280	77.52789524	162.136919	–1.0644	2.89E–05
AT1G07360	53.47389433	112.0347257	–1.067	0.00075613
AT1G17290	124.7570009	261.6343131	–1.0684	3.44E–08
AT2G30490	690.9511761	1449.899971	–1.0693	1.40E–43
AT1G14900	51.20726732	107.4628416	–1.0694	0.00098901
AT2G18690	210.8425695	442.5772294	–1.0698	1.52E–13
AT4G25810	126.6073086	265.9234002	–1.0707	2.39E–08
AT5G65630	193.9585112	408.2173997	–1.0736	1.28E–12
AT1G27970	59.44113686	125.1376511	–1.074	0.00028589
AT1G32700	43.99106705	92.71026732	–1.0755	0.002432

AT1G67580	64.29819474	135.5540055	–1.076	0.00013746
AT1G19770	254.5560904	536.9842784	–1.0769	1.44E–16
AT5G08500	46.53524022	98.17767505	–1.0771	0.0016617
AT5G08630	38.76394762	81.86971751	–1.0786	0.0048809
AT1G11400	43.6210055	92.19180624	–1.0796	0.0024049
AT3G63070	65.26960631	137.9577796	–1.0797	0.00011014
AT5G27380	83.86519932	177.50222	–1.0817	7.18E–06
AT5G03730	92.70041889	196.6381471	–1.0849	1.79E–06
AT5G25280	84.18900318	178.586275	–1.0849	6.23E–06
AT5G16830	75.72384517	160.9585983	–1.0879	1.96E–05
AT5G01100	94.1344074	200.2202418	–1.0888	1.26E–06
AT1G25220	46.81278638	99.78019111	–1.0919	0.0012502
AT3G45980	124.2481662	265.1221422	–1.0934	1.18E–08
AT2G15970	222.7770546	476.937059	–1.0982	2.51E–15
AT2G31810	142.3349246	304.9022467	–1.0991	5.71E–10
AT4G24060	62.81794853	134.6584818	–1.1001	9.82E–05

AT5G65300	68.69267567	147.4786103	–1.1023	3.81E–05
AT1G64280	53.47389433	114.8626952	–1.103	0.00037737
AT5G39670	39.45781303	84.8390855	–1.1044	0.0030394
AT5G46730	38.39388607	82.62384271	–1.1057	0.0034976
AT4G36730	73.9197951	159.3089494	–1.1078	1.49E–05
AT5G47220	43.66726319	94.12425208	–1.108	0.0015247
AT4G19200	183.8280762	396.7641232	–1.1099	4.50E–13
AT5G18650	74.52114512	161.052864	–1.1118	1.21E–05
AT1G51760	70.58924113	152.6632211	–1.1128	2.16E–05
AT5G14120	123.8781047	268.138643	–1.1141	4.67E–09
AT3G52240	40.79928616	88.42118022	–1.1158	0.0020877
AT5G55100	47.36787871	102.7024263	–1.1165	0.00074656
AT1G08930	341.705586	741.7764042	–1.1182	1.85E–24
AT2G18440	152.8816789	332.0036213	–1.1188	3.55E–11
AT5G43830	170.9684372	371.6423273	–1.1202	1.73E–12
AT4G37120	90.66508035	197.3451394	–1.1221	6.69E–07

AT3G53110	101.7206692	221.5242788	–1.1229	1.11E–07
AT2G18750	126.699824	276.1040905	–1.1238	1.85E–09
AT4G14500	61.01389846	133.6215597	–1.1309	6.30E–05
AT1G25682	44.59241707	97.7063468	–1.1317	0.00088808
AT2G05520	34.69327054	76.02524717	–1.1318	0.0043692
AT2G46740	43.89855166	96.19809639	–1.1318	0.00098901
AT3G10720	43.80603627	96.00956509	–1.132	0.00099952
AT4G12040	130.2154088	285.3892571	–1.132	6.91E–10
AT3G19190	68.41512951	149.97665	–1.1324	1.81E–05
AT1G51680	145.6192209	319.4191569	–1.1333	4.99E–11
AT3G45970	122.7216623	269.3169636	–1.1339	2.17E–09
AT1G55810	87.05698021	191.265005	–1.1355	7.54E–07
AT3G05545	65.96347172	144.9334377	–1.1357	2.48E–05
AT5G20000	44.96247862	98.83753461	–1.1363	0.00077027
AT5G01830	57.22076755	125.8446435	–1.137	0.00010152
AT3G62240	41.12309002	90.49502453	–1.1379	0.0014029

AT4G21660	173.8364143	382.8599397	–1.1391	2.94E–13
AT1G11960	73.31844508	161.5241922	–1.1395	6.59E–06
AT4G20380	40.89180155	90.16509476	–1.1408	0.0013931
AT2G04400	49.68076342	109.5838188	–1.1413	0.00032194
AT3G03790	39.13400917	86.34733591	–1.1417	0.0018324
AT5G26760	34.64701285	76.6379739	–1.1453	0.0036549
AT2G19270	36.26603214	80.31433427	–1.147	0.0027262
AT4G17140	129.7990895	287.6516327	–1.148	3.21E–10
AT5G57580	71.79194118	159.4032151	–1.1508	6.17E–06
AT4G19860	109.5844772	243.3467769	–1.151	9.05E–09
AT5G62640	41.40063618	92.00327494	–1.152	0.001065
AT2G03470	39.41155534	87.66705502	–1.1534	0.0014615
AT5G35690	42.74210931	95.11404141	–1.154	0.00082298
AT3G08730	82.80127236	184.2893468	–1.1542	8.18E–07
AT4G27960	122.5366316	272.8519255	–1.1549	7.95E–10
AT4G02020	59.30236378	132.1133093	–1.1556	4.65E–05

AT3G17860	37.00615525	82.52957706	–1.1571	0.0020728
AT5G11260	86.91820713	193.9044432	–1.1576	3.58E–07
AT2G30600	88.76851489	198.1935303	–1.1588	2.49E–07
AT2G27830	58.70101376	131.1706527	–1.16	4.65E–05
AT3G45960	69.15525261	154.5485341	–1.1601	7.44E–06
AT5G58020	49.7732788	111.3277333	–1.1614	0.00021247
AT1G47380	42.55707853	95.25543989	–1.1624	0.00073346
AT5G46410	59.71868303	134.1871536	–1.168	3.18E–05
AT5G53550	47.59916718	106.9915134	–1.1685	0.00026976
AT3G10550	32.14909737	72.3488868	–1.1702	0.0040321
AT2G04865	31.22394349	70.27504249	–1.1704	0.0047486
AT2G16365	45.33254018	102.231098	–1.1732	0.00037023
AT1G61690	58.00714835	130.840723	–1.1735	3.78E–05
AT5G47120	127.763751	288.3114922	–1.1741	1.13E–10
AT5G50430	30.71510885	69.33238598	–1.1746	0.0049159
AT5G50200	55.37045979	125.0433855	–1.1752	5.86E–05

AT1G16670	35.66468212	80.5499984	–1.1754	0.0020036
AT5G12480	77.43537985	175.0513131	–1.1767	1.01E–06
AT3G54140	35.43339365	80.1729358	–1.178	0.0020073
AT1G08940	38.67143223	87.52565654	–1.1784	0.0011172
AT3G24500	42.32579006	95.82103379	–1.1788	0.00057556
AT1G78420	53.47389433	121.0842281	–1.1791	7.57E–05
AT3G07280	34.69327054	78.66468539	–1.1811	0.0021926
AT2G32240	189.7953187	430.4640933	–1.1814	6.69E–16
AT3G45830	46.53524022	105.5775286	–1.1819	0.00025138
AT2G38230	37.56124757	85.31041375	–1.1835	0.0012609
AT3G57550	78.73059528	179.7174628	–1.1907	5.00E–07
AT2G20560	34.2306936	78.14622431	–1.1909	0.0020722
AT5G45190	60.36629074	137.9577796	–1.1924	1.54E–05
AT5G63160	187.2974032	428.90871	–1.1953	3.44E–16
AT2G40000	866.2215789	1988.015437	–1.1985	2.26E–74
AT4G18710	149.0422902	342.1843115	–1.1991	4.51E–13

AT4G01120	86.08556864	198.2877959	–1.2038	7.69E–08
AT4G31860	61.4764754	141.7284056	–1.205	8.90E–06
AT1G28200	65.03831784	150.0709157	–1.2063	4.27E–06
AT4G39980	175.9642682	406.1435554	–1.2067	1.27E–15
AT3G02340	28.7722857	66.55154929	–1.2098	0.0045254
AT1G12610	48.38554798	111.94046	–1.2101	0.0001002
AT5G13750	72.76335275	168.3584519	–1.2103	8.24E–07
AT1G03290	42.64959392	98.79040178	–1.2118	0.00029601
AT1G55500	35.34087826	81.96398316	–1.2137	0.0011994
AT1G70590	48.98689801	113.6372417	–1.214	8.24E–05
AT5G51980	38.76394762	90.07082911	–1.2163	0.00058751
AT4G36780	90.89636882	211.4378542	–1.2179	1.70E–08
AT2G35710	49.31070186	114.8626952	–1.2199	6.76E–05
AT1G75380	59.30236378	138.3348422	–1.222	8.69E–06
AT3G53260	353.4550403	824.7301767	–1.2224	4.09E–32
AT1G62180	86.08556864	200.8801013	–1.2225	3.76E–08

AT5G50210	36.49732061	85.16901528	–1.2225	0.00083015
AT1G21110	59.85745611	139.7959598	–1.2237	7.39E–06
AT3G18290	78.31427604	182.9224949	–1.2239	1.73E–07
AT5G50960	46.95155947	109.7252172	–1.2247	9.81E–05
AT1G04985	34.92455901	81.68118621	–1.2258	0.0010771
AT4G02380	361.365106	847.2596672	–1.2293	2.52E–33
AT2G42680	107.1790771	251.689287	–1.2316	3.26E–10
AT4G15760	99.13023836	232.8832897	–1.2322	1.68E–09
AT5G11740	240.0774322	564.0856529	–1.2324	2.08E–22
AT2G06025	43.25094394	101.7597698	–1.2344	0.0001704
AT1G53580	99.82410377	234.9100012	–1.2346	1.31E–09
AT1G14860	42.41830545	99.92158959	–1.2361	0.00019536
AT3G06760	43.6210055	102.8909576	–1.238	0.00014657
AT1G53840	244.4719131	577.0000471	–1.2389	3.92E–23
AT1G79245	878.5723832	2073.891445	–1.2391	1.42E–82
AT1G22410	298.5471574	704.7771364	–1.2392	3.39E–28

AT1G08720	36.21977445	85.64034353	–1.2415	0.00064336
AT3G51130	38.62517454	91.62621234	–1.2462	0.00035859
AT3G03310	34.2306936	81.35125643	–1.2489	0.00086766
AT2G27550	42.18701698	100.2986522	–1.2494	0.00015852
AT1G05960	78.22176065	186.127527	–1.2506	6.72E–08
AT2G07783	28.54099723	67.91840123	–1.2508	0.0027963
AT2G38410	41.07683232	97.84774528	–1.2522	0.0001904
AT3G08690	33.53682819	79.89013885	–1.2523	0.00095368
AT3G06860	110.1395695	262.8126337	–1.2547	5.32E–11
AT2G44080	31.8715512	76.11951282	–1.256	0.0012831
AT4G30210	266.6756062	637.1415321	–1.2565	3.60E–26
AT5G04770	25.7192779	61.46120416	–1.2568	0.0047535
AT1G02170	38.99523609	93.2287284	–1.2575	0.00026964
AT4G17900	86.68691866	207.2901656	–1.2578	7.97E–09
AT3G46640	50.32837113	120.4715014	–1.2592	2.22E–05
AT1G09060	34.36946668	82.57670989	–1.2646	0.00065409

AT5G62460	34.55449746	83.04803814	–1.2651	0.00062419
AT4G30490	45.24002479	108.9239592	–1.2676	5.65E–05
AT2G38560	26.41314331	63.72357978	–1.2706	0.0034226
AT3G07310	25.16418557	60.84847743	–1.2738	0.0043675
AT5G10400	33.95314744	82.10538163	–1.2739	0.00061796
AT1G34220	28.54099723	69.09672186	–1.2756	0.0020073
AT1G63090	38.1163399	92.33320472	–1.2764	0.00023183
AT2G20900	46.99781716	113.9671715	–1.278	3.02E–05
AT3G53540	28.72602801	69.75658141	–1.28	0.001818
AT1G66480	40.89180155	99.30886286	–1.2801	0.00011538
AT4G32600	29.23486264	71.17056617	–1.2836	0.0015353
AT4G25170	70.95930268	172.8360703	–1.2843	1.02E–07
AT1G45145	177.814576	434.046188	–1.2875	9.41E–19
AT4G21680	27.47707027	67.1171432	–1.2885	0.0021474
AT1G15230	32.42664353	79.41881059	–1.2923	0.00065058
AT1G14040	88.53722642	217.6593871	–1.2977	9.45E–10

AT1G60940	73.27218739	180.3301895	–1.2993	3.47E–08
AT5G19340	24.05400091	59.24596137	–1.3004	0.0040968
AT1G73920	61.98531004	152.8988852	–1.3026	4.64E–07
AT4G21390	29.37363573	72.5374181	–1.3042	0.0011111
AT5G63320	62.03156773	153.1816822	–1.3042	4.36E–07
AT1G14000	64.94580246	160.3930044	–1.3043	2.15E–07
AT4G16130	32.79670509	81.02132665	–1.3048	0.00048783
AT4G33910	27.93964721	69.04958903	–1.3053	0.0015329
AT2G33770	74.84494898	185.043472	–1.3059	1.85E–08
AT5G52580	50.6059173	125.2790496	–1.3078	6.31E–06
AT1G30720	58.65475606	145.2633675	–1.3084	8.67E–07
AT4G11360	46.44272483	115.0512265	–1.3088	1.69E–05
AT1G25230	41.81695543	103.8336141	–1.3121	4.83E–05
AT3G50950	66.47230636	165.2005526	–1.3134	1.10E–07
AT3G54620	91.63649193	228.21714	–1.3164	1.87E–10
AT5G09390	33.81437436	84.22635877	–1.3166	0.00031162

AT1G19850	25.53424712	63.62931413	–1.3173	0.0023306
AT4G35750	337.0335589	840.849603	–1.319	1.27E–37
AT1G34300	33.07425125	82.52957706	–1.3192	0.0003589
AT3G06250	22.5737547	56.46512468	–1.3227	0.0045189
AT2G46500	144.9716132	363.1584188	–1.3248	1.43E–16
AT3G12500	22.15743545	55.52246818	–1.3253	0.0048711
AT5G12310	23.96148552	60.0472194	–1.3254	0.0030879
AT3G15630	142.3349246	357.4082141	–1.3283	2.18E–16
AT1G03230	50.97597885	128.0127535	–1.3284	3.35E–06
AT3G48520	107.0865617	269.0341667	–1.329	1.88E–12
AT5G23050	99.26901144	249.521177	–1.3297	1.36E–11
AT3G20060	25.53424712	64.19490803	–1.33	0.0019764
AT1G01340	36.17351675	91.20201691	–1.3341	0.0001267
AT1G09940	84.97538398	214.8314176	–1.3381	3.76E–10
AT5G54810	179.017276	452.8993181	–1.3391	5.77E–21
AT1G27290	71.00556038	179.9059941	–1.3412	1.27E–08

AT4G35860	26.73694716	67.82413557	–1.343	0.0012265
AT2G20670	143.8151708	364.8552005	–1.3431	4.75E–17
AT4G21980	65.63966787	166.5202717	–1.3431	4.88E–08
AT1G63800	42.18701698	107.085779	–1.3439	2.20E–05
AT4G30780	53.47389433	135.9310681	–1.346	1.08E–06
AT2G46140	47.13659024	119.9059075	–1.347	5.62E–06
AT1G75220	74.19734127	188.8140981	–1.3475	4.26E–09
AT1G73880	21.41731235	54.57981167	–1.3496	0.0044582
AT1G32230	401.5630421	1025.13895	–1.3521	6.72E–48
AT4G27585	30.02124344	76.73223955	–1.3538	0.00044166
AT3G24070	35.89597059	91.86187647	–1.3556	9.05E–05
AT1G56140	107.8266848	276.8582157	–1.3604	2.51E–13
AT5G54540	55.50923287	142.5296636	–1.3605	4.10E–07
AT4G18340	131.5106242	337.9423572	–1.3616	3.32E–16
AT2G15490	42.788367	110.1965455	–1.3648	1.17E–05
AT5G54500	279.1651836	718.9641168	–1.3648	3.20E–34

AT5G18490	33.67560127	86.86579699	–1.3671	0.0001325
AT4G04620	24.65535093	63.62931413	–1.3678	0.0015123
AT1G76650	175.6404643	453.8419746	–1.3696	7.31E–22
AT1G73260	276.0196604	713.4024434	–1.3699	3.45E–34
AT3G26020	33.67560127	87.05432829	–1.3702	0.00012508
AT1G62300	52.22493659	135.0826772	–1.371	7.33E–07
AT1G31930	55.37045979	143.519453	–1.3741	2.81E–07
AT1G23390	28.49473954	74.04566851	–1.3777	0.00045718
AT4G30280	167.7766563	436.5913605	–1.3797	2.57E–21
AT4G23100	283.6059223	738.3828408	–1.3805	7.28E–36
AT2G44410	29.00357417	75.55391892	–1.3813	0.00037631
AT3G62650	85.99305325	224.0223185	–1.3814	3.70E–11
AT1G71950	52.54874045	136.9208574	–1.3816	4.94E–07
AT4G27520	92.56164581	241.1786669	–1.3816	5.55E–12
AT3G51600	21.0009931	54.8154758	–1.3841	0.0033433
AT3G47160	37.70002066	98.46047201	–1.385	3.05E–05

AT5G51070	106.7165002	278.7435287	–1.3852	7.75E–14
AT1G22930	214.1268658	559.8908315	–1.3867	1.76E–27
AT1G02360	40.01290536	104.6820049	–1.3875	1.51E–05
AT4G33540	29.97498575	78.61755256	–1.3911	0.00024271
AT3G59220	34.2306936	89.92943063	–1.3935	6.93E–05
AT4G39670	22.38872392	58.86889877	–1.3947	0.0019975
AT3G30775	1105.928949	2908.472382	–1.395	1.59E–143
AT2G36310	92.00655348	241.979925	–1.3951	3.19E–12
AT2G31260	33.4905705	88.09125044	–1.3952	8.31E–05
AT4G37790	50.18959805	132.0190436	–1.3953	6.52E–07
AT1G29930	50.18959805	132.0661764	–1.3958	6.44E–07
AT2G39660	50.65217499	133.2916299	–1.3959	5.63E–07
AT3G59110	20.67718924	54.4384132	–1.3966	0.0031713
AT5G17760	34.73952824	91.62621234	–1.3992	5.35E–05
AT3G17000	44.12984013	116.4180784	–1.3995	3.43E–06
AT3G61070	33.76811666	89.17530543	–1.401	6.85E–05

AT5G27030	42.0482439	111.2806005	–1.4041	5.67E–06
AT4G33980	29.69743958	78.61755256	–1.4045	0.00020983
AT5G10980	185.7246416	491.8310318	–1.405	9.69E–25
AT5G12010	44.73119015	118.4919227	–1.4054	2.47E–06
AT5G17380	94.9207882	251.4536229	–1.4055	7.64E–13
AT4G36648	163.5672062	433.7162582	–1.4069	6.53E–22
AT1G12200	43.5747478	115.6168204	–1.4078	3.28E–06
AT2G23320	65.68592556	174.2971879	–1.4079	4.42E–09
AT4G14370	27.43081258	72.9144807	–1.4104	0.00037189
AT3G29160	69.20151031	184.0065499	–1.4109	1.38E–09
AT4G16790	19.84455075	52.78876431	–1.4115	0.003404
AT4G17030	19.75203536	52.55310018	–1.4118	0.0034827
AT2G35510	50.32837113	133.9514894	–1.4123	3.84E–07
AT2G02220	57.31328294	152.8046196	–1.4147	4.36E–08
AT3G62150	54.95414054	146.6302194	–1.4159	8.56E–08
AT4G05020	84.55906473	225.9076316	–1.4177	9.38E–12

AT1G11330	29.65118189	79.27741212	–1.4188	0.00016674
AT4G24590	31.27020118	83.75503052	–1.4214	9.81E–05
AT5G01750	132.8058396	355.8528308	–1.422	2.23E–18
AT1G17060	18.45681993	49.48946654	–1.423	0.0045584
AT5G16630	24.37780477	65.37322866	–1.4231	0.00076485
AT1G72360	37.88505143	101.7126369	–1.4248	1.23E–05
AT3G23150	20.72344694	55.66386665	–1.4255	0.0022289
AT1G30420	49.0331557	132.1133093	–1.4299	3.40E–07
AT2G47450	21.64860082	58.35043769	–1.4305	0.0015839
AT3G16800	26.59817408	71.7832929	–1.4323	0.00033857
AT2G42330	25.58050481	69.14385468	–1.4346	0.00044705
AT1G13195	19.84455075	53.73142082	–1.437	0.0025439
AT5G47450	46.21143636	125.5147137	–1.4415	5.93E–07
AT3G20660	34.2306936	93.08732992	–1.4433	2.56E–05
AT2G24100	41.95572851	114.10857	–1.4435	2.19E–06
AT5G07460	28.26345107	76.87363803	–1.4436	0.00016738

AT3G05580	44.22235552	120.3301029	–1.4441	1.04E–06
AT1G77220	27.75461643	75.64818457	–1.4466	0.00018706
AT1G15110	50.42088652	137.4864513	–1.4472	1.32E–07
AT4G16760	167.5453679	456.9527411	–1.4475	3.14E–24
AT1G53440	45.05499401	122.9695412	–1.4485	7.04E–07
AT5G13800	34.78578593	95.16117424	–1.4519	1.79E–05
AT2G41430	727.1246929	1994.142705	–1.4555	5.08E–106
AT1G03370	62.44788698	171.6106168	–1.4584	1.75E–09
AT4G15420	21.09350849	58.06764074	–1.4609	0.0012819
AT1G09950	17.99424299	49.58373219	–1.4623	0.0034183
AT1G55450	46.07266328	127.0700969	–1.4636	3.32E–07
AT3G05500	94.78201512	261.6814459	–1.4651	2.52E–14
AT1G18570	41.72444004	115.3340235	–1.4669	1.29E–06
AT1G73730	18.41056223	50.95058413	–1.4686	0.0027936
AT1G53190	26.27437022	72.7259494	–1.4688	0.00020931
AT5G63620	57.91463296	160.2987388	–1.4688	5.35E–09

AT5G56870	160.8380022	445.3580661	–1.4694	3.10E–24
AT5G60200	25.44173173	70.46357379	–1.4697	0.00027141
AT1G17170	113.5163812	314.8472729	–1.4718	2.76E–17
AT4G18140	21.50982774	59.6701568	–1.472	0.00096958
AT5G06560	40.75302846	113.1187807	–1.4729	1.53E–06
AT4G30390	24.00774322	66.78721342	–1.4761	0.00039663
AT2G23790	46.85904408	130.5107932	–1.4778	1.70E–07
AT1G32530	39.04149378	109.0653577	–1.4821	2.17E–06
AT2G30250	42.18701698	118.0677273	–1.4847	6.83E–07
AT1G50460	60.45880613	169.2068427	–1.4848	1.24E–09
AT1G30700	79.14691453	221.5242788	–1.4849	1.81E–12
AT3G12350	24.8866394	69.75658141	–1.487	0.00024935
AT1G34260	30.48382038	85.45181223	–1.4871	3.66E–05
AT5G11950	16.93031602	47.46275505	–1.4872	0.0037289
AT5G19230	107.8729425	302.4513398	–1.4874	6.47E–17
AT2G33700	33.4905705	93.93572078	–1.4879	1.29E–05

AT1G42990	73.36470277	205.8761808	–1.4886	1.14E–11
AT4G26260	30.25253191	85.07474963	–1.4917	3.63E–05
AT2G40970	31.22394349	88.09125044	–1.4963	2.36E–05
AT5G57910	36.08100136	101.8069026	–1.4965	4.28E–06
AT1G09070	540.0585781	1524.369835	–1.497	4.60E–85
AT5G47370	33.58308588	94.87837728	–1.4983	9.94E–06
AT1G27300	45.24002479	128.0127535	–1.5006	1.51E–07
AT1G30730	73.9197951	209.5054084	–1.503	4.74E–12
AT5G23340	24.23903169	68.76679208	–1.5044	0.00023729
AT3G14590	51.02223654	144.7920393	–1.5048	1.69E–08
AT1G20780	41.30812079	117.6906647	–1.5105	4.65E–07
AT4G05010	21.41731235	61.03700873	–1.5109	0.00058988
AT1G26270	198.214219	566.6308255	–1.5153	2.15E–32
AT4G29670	76.00139133	217.3294574	–1.5158	1.16E–12
AT3G63010	35.29462056	100.9585117	–1.5162	3.58E–06
AT5G48970	19.28945842	55.1925384	–1.5167	0.0011687

AT5G13200	53.42763664	153.2759478	–1.5205	4.05E–09
AT3G09440	615.9674541	1771.770035	–1.5243	5.63E–102
AT1G19400	51.11475193	147.1486805	–1.5255	7.99E–09
AT4G38470	342.0293899	985.0760483	–1.5261	7.73E–57
AT2G34500	24.42406246	70.36930814	–1.5266	0.00015619
AT4G08555	29.41989342	84.79195267	–1.5271	2.45E–05
AT4G14040	50.28211344	144.9805706	–1.5277	1.01E–08
AT3G13110	78.12924526	225.4363033	–1.5288	2.65E–13
AT1G29520	19.24320073	55.66386665	–1.5324	0.00097374
AT2G46260	81.55231462	236.0883218	–1.5335	5.58E–14
AT4G29950	98.89894989	286.5204449	–1.5346	6.64E–17
AT3G51500	19.93706614	57.78484379	–1.5352	0.00072392
AT4G28260	89.78618416	260.314594	–1.5357	2.04E–15
AT5G16110	267.2306986	775.5235071	–1.5371	2.49E–45
AT5G43620	30.80762424	89.55236803	–1.5394	1.14E–05
AT5G58787	24.05400091	69.94511271	–1.5399	0.00014395

AT3G25495	14.8024621	43.0794023	–1.5412	0.0045872
AT5G01600	58.83978684	171.3278199	–1.5419	2.32E–10
AT1G72680	30.85388194	89.92943063	–1.5433	1.03E–05
AT1G56220	152.3265865	444.3211439	–1.5444	2.71E–26
AT1G55020	127.763751	372.7263823	–1.5446	4.01E–22
AT1G61340	59.07107531	172.4118749	–1.5453	1.84E–10
AT2G35800	34.64701285	101.3355743	–1.5483	2.14E–06
AT2G46270	45.74885942	133.810091	–1.5484	2.93E–08
AT4G28300	91.91403809	269.1755652	–1.5502	3.55E–16
AT5G46180	38.62517454	113.2130463	–1.5514	4.21E–07
AT4G14680	24.8866394	72.96161353	–1.5518	8.67E–05
AT4G38810	40.75302846	119.5288449	–1.5524	1.80E–07
AT2G36320	347.6265708	1020.095737	–1.5531	1.26E–60
AT4G02880	40.66051308	119.6231106	–1.5568	1.65E–07
AT2G41250	26.04308175	76.8265052	–1.5607	4.71E–05
AT1G80120	15.31129673	45.24751227	–1.5632	0.0029974

AT5G14730	46.30395175	137.0622559	–1.5656	1.34E–08
AT4G00550	18.82688148	55.7581323	–1.5664	0.00073346
AT4G34710	336.570982	998.1789737	–1.5684	2.59E–60
AT1G01770	31.45523196	93.46439253	–1.5711	4.44E–06
AT4G36670	29.97498575	89.17530543	–1.5729	7.80E–06
AT2G42750	18.13301607	53.96708494	–1.5735	0.00088215
AT5G11070	78.6380799	234.0616103	–1.5736	1.81E–14
AT2G44790	466.0000099	1387.590376	–1.5742	2.60E–84
AT1G77770	20.39964308	60.75421178	–1.5744	0.00035243
AT5G46910	43.48223241	129.5681367	–1.5752	3.11E–08
AT2G26660	23.96148552	71.5947616	–1.5791	7.85E–05
AT5G57610	17.57792374	52.60023301	–1.5813	0.00099632
AT3G12740	94.87453051	283.9281395	–1.5814	1.31E–17
AT5G60270	15.86638906	47.55702071	–1.5837	0.0019322
AT5G27520	32.61167431	97.84774528	–1.5852	2.02E–06
AT4G11850	103.8485232	311.830772	–1.5863	2.10E–19

AT3G20340	34.55449746	103.7864813	–1.5867	8.63E–07
AT5G04340	122.9992085	370.1812097	–1.5896	4.81E–23
AT1G03740	71.51439501	216.2454024	–1.5964	1.05E–13
AT4G31780	27.15326641	82.10538163	–1.5964	1.54E–05
AT4G09150	80.71967613	244.2894334	–1.5976	1.86E–15
AT5G22920	126.4222779	383.2370023	–1.6	4.23E–24
AT5G55700	21.09350849	64.00637673	–1.6014	0.00017755
AT3G49845	15.21878134	46.28443442	–1.6047	0.0019971
AT5G66650	78.86936837	240.094612	–1.6061	2.47E–15
AT5G47230	78.82311067	240.1888776	–1.6075	2.33E–15
AT3G45730	50.97597885	155.3969249	–1.6081	4.25E–10
AT1G06760	151.4014326	462.2316175	–1.6102	2.65E–29
AT3G22530	15.31129673	46.75576268	–1.6105	0.001803
AT1G21000	57.54457141	175.8997039	–1.612	2.06E–11
AT5G11530	26.69068947	81.63405338	–1.6128	1.35E–05
AT1G69830	25.6730202	78.52328691	–1.6129	2.08E–05

AT1G01650	68.4613872	209.6468068	–1.6146	1.52E–13
AT4G35985	51.57732887	158.083496	–1.6159	2.43E–10
AT1G66500	58.65475606	179.9059941	–1.6169	1.02E–11
AT1G02400	60.13500227	184.525011	–1.6175	5.15E–12
AT4G20780	35.52590904	109.0182249	–1.6176	2.58E–07
AT2G39350	33.4905705	102.8438248	–1.6186	6.12E–07
AT4G00755	16.83780064	51.70470933	–1.6186	0.00085671
AT1G59740	28.12467799	86.58300004	–1.6222	5.96E–06
AT3G15070	22.15743545	68.248331	–1.623	8.04E–05
AT1G20440	1571.558898	4849.637791	–1.6257	0
AT3G10420	15.86638906	48.97100546	–1.626	0.0011913
AT1G54710	29.88247036	92.23893907	–1.6261	2.52E–06
AT2G46030	20.90847771	64.76050193	–1.631	0.0001214
AT4G05050	574.6593333	1782.799116	–1.6334	3.74E–115
AT2G25900	93.67183046	290.809532	–1.6344	5.04E–19
AT4G32480	127.2549163	395.7743339	–1.637	8.10E–26

AT5G04760	29.51240881	91.86187647	–1.6381	2.26E–06
AT1G62770	35.43339365	110.3850768	–1.6394	1.51E–07
AT5G64300	88.99980336	277.2824111	–1.6395	3.06E–18
AT1G76180	993.8928145	3096.720886	–1.6396	3.03E–201
AT5G54730	48.52432107	151.2021035	–1.6397	3.84E–10
AT2G22470	160.2829099	500.2678075	–1.6421	1.05E–32
AT4G17260	17.67043913	55.33393688	–1.6468	0.00040892
AT1G18270	292.9962342	919.5142883	–1.65	1.97E–60
AT1G23870	171.9398488	540.2364433	–1.6517	1.21E–35
AT2G02180	69.61782955	219.4504345	–1.6564	9.28E–15
AT4G21470	28.07842029	88.5625787	–1.6572	2.86E–06
AT4G13830	19.75203536	62.30959502	–1.6575	0.00013564
AT1G35670	68.09132565	215.2084802	–1.6602	1.55E–14
AT2G34910	12.25828892	38.7903152	–1.6619	0.0040824
AT1G19310	15.77387367	50.00792762	–1.6646	0.00077927
AT4G21410	81.22851076	257.5808901	–1.665	2.14E–17

AT2G30620	138.0792168	437.9582125	–1.6653	2.43E–29
AT3G54640	74.89120668	237.5965722	–1.6656	4.35E–16
AT5G08660	28.35596646	89.97656346	–1.6659	2.07E–06
AT5G55180	46.21143636	146.7244851	–1.6668	4.12E–10
AT5G62540	42.41830545	134.7056146	–1.6671	2.49E–09
AT5G56550	23.3601355	74.70552807	–1.6772	1.78E–05
AT5G07440	984.132441	3147.812869	–1.6774	2.46E–212
AT3G43230	45.61008634	145.9232271	–1.6778	3.67E–10
AT5G39660	27.93964721	89.41096955	–1.6781	1.93E–06
AT3G23250	90.80385343	290.8566648	–1.6795	7.14E–20
AT4G39675	27.80087413	89.1281726	–1.6808	1.94E–06
AT3G58750	63.88187549	205.02779	–1.6823	3.72E–14
AT2G41630	103.8947809	333.4647388	–1.6824	8.51E–23
AT1G66580	336.3396935	1080.425754	–1.6836	2.34E–73
AT5G13310	12.81338125	41.24122212	–1.6864	0.0024632
AT1G68440	262.2811253	844.4316977	–1.6869	1.58E–57

AT1G20450	553.7971132	1783.223312		–1.6871	1.98E–121
AT4G34310	26.13559714	84.2734916		–1.6891	3.64E–06
AT2G19800	187.7599802	605.5154063		–1.6893	2.11E–41
AT3G44300	239.5685975	772.6484048		–1.6894	9.05E–53
AT1G74750	12.53583509	40.43996409		–1.6897	0.0027272
AT5G06570	30.25253191	97.65921398		–1.6907	4.59E–07
AT5G53970	23.91522783	77.20356781		–1.6907	1.05E–05
AT5G62480	26.78320486	86.58300004		–1.6928	2.44E–06
AT4G34890	104.2185847	337.0939664		–1.6935	2.79E–23
AT2G33830	472.2910563	1528.611789		–1.6945	7.55E–105
AT5G17650	17.1153468	55.61673383		–1.7002	0.00025579
AT2G23450	61.4764754	199.9845777		–1.7018	4.56E–14
AT5G05730	54.53782129	177.5493528		–1.7029	1.48E–12
AT3G43430	51.71610195	168.4055847		–1.7033	6.20E–12
AT1G62320	11.74945429	38.31898695		–1.7055	0.0034226
AT4G27450	40.75302846	132.9145673		–1.7055	1.53E–09

AT4G39090	363.5854753	1188.12426	–1.7083	1.42E–82
AT5G12340	32.24161276	105.4361301	–1.7094	1.05E–07
AT1G32170	135.9976205	444.8867378	–1.7099	4.35E–31
AT3G20500	28.7722857	94.12425208	–1.7099	6.04E–07
AT4G30790	36.40480522	119.1989151	–1.7112	1.18E–08
AT2G02710	66.37979097	217.9421841	–1.7151	1.74E–15
AT1G02750	14.89497749	48.92387264	–1.7157	0.00063825
AT2G30870	712.7848077	2346.743371	–1.7191	1.20E–164
AT4G11280	272.5965911	897.4561261	–1.7191	4.97E–63
AT4G23730	15.08000826	49.67799784	–1.72	0.00055199
AT1G63440	49.91205189	165.624748	–1.7305	4.93E–12
AT3G49590	28.40222415	94.26565056	–1.7307	4.43E–07
AT4G34230	121.1951584	402.2315309	–1.7307	1.10E–28
AT3G18830	48.52432107	161.0999968	–1.7312	1.00E–11
AT1G08800	55.97180981	186.0332614	–1.7328	1.74E–13
AT5G02490	24.28528938	80.78566253	–1.734	3.64E–06

AT5G15960	22.24995084	74.04566851	–1.7346	1.06E–05
AT1G15800	14.10859669	47.03855963	–1.7373	0.00073557
AT1G15430	25.90430867	86.39446873	–1.7377	1.41E–06
AT4G33030	18.73436609	62.54525914	–1.7392	6.27E–05
AT3G05360	15.49632751	51.89324063	–1.7436	0.00032581
AT3G26460	42.09450159	140.9742804	–1.7437	1.97E–10
AT5G52660	11.33313504	37.98905717	–1.745	0.0029099
AT3G50930	104.773677	351.8936735	–1.7479	1.58E–25
AT4G08770	41.07683232	138.0049124	–1.7483	2.90E–10
AT5G13330	16.69902755	56.27659338	–1.7528	0.00015067
AT5G18400	55.13917132	185.9389957	–1.7537	9.89E–14
AT1G70290	117.7258314	397.4239827	–1.7552	5.57E–29
AT4G14630	42.92714008	145.0748362	–1.7568	7.57E–11
AT5G46050	10.73178502	36.29227546	–1.7578	0.0035484
AT2G03270	10.68552733	36.19800981	–1.7603	0.0035557
AT1G14870	270.9775718	918.5716318	–1.7612	4.72E–67

AT4G22212	325.6541662	1106.631605	–1.7648	5.19E–81
AT4G15260	16.00516214	54.48554602	–1.7673	0.00017907
AT4G32070	82.75501467	281.7128967	–1.7673	7.32E–21
AT2G29670	10.59301194	36.15087699	–1.7709	0.0033918
AT5G66170	60.50506383	206.5360404	–1.7713	1.88E–15
AT2G26670	52.96505969	180.9900491	–1.7728	1.33E–13
AT1G28260	13.78479283	47.1799581	–1.7751	0.00055802
AT2G34930	26.87572025	92.00327494	–1.7754	3.48E–07
AT3G49570	11.14810427	38.17758848	–1.7759	0.0023827
AT1G76590	24.42406246	83.84929617	–1.7795	1.27E–06
AT5G18470	31.31645888	107.6042401	–1.7807	2.43E–08
AT1G62570	65.54715248	225.8604987	–1.7848	4.69E–17
AT1G52240	26.18185484	90.44789171	–1.7885	3.77E–07
AT2G32140	15.68135829	54.20274907	–1.7893	0.00015769
AT1G78850	153.3442558	530.3856829	–1.7903	8.55E–40
AT3G51430	15.86638906	55.09827275	–1.796	0.00012902

AT4G32190	20.26087	70.65210509	–1.802	9.03E–06
AT3G13100	12.72086587	44.44625424	–1.8049	0.00071681
AT4G33150	139.7907514	488.9559294	–1.8064	3.18E–37
AT5G55970	22.15743545	77.53349758	–1.807	2.66E–06
AT4G21990	276.9448143	969.8521457	–1.8082	9.08E–74
AT5G40690	39.73535919	139.6545613	–1.8134	5.72E–11
AT3G50910	48.10800182	169.1125771	–1.8136	3.44E–13
AT5G59690	74.61366051	262.3884383	–1.8142	2.91E–20
AT3G14780	13.27595819	46.75576268	–1.8163	0.00044937
AT5G49665	13.41473128	47.41562223	–1.8215	0.00038813
AT3G02555	9.714115752	34.35982963	–1.8226	0.0035005
AT5G18670	142.0573784	502.4830503	–1.8226	8.95E–39
AT3G57750	10.36172347	36.66933807	–1.8233	0.0023685
AT4G03320	20.67718924	73.48007461	–1.8293	4.16E–06
AT5G23360	19.24320073	68.6253936	–1.8344	9.17E–06
AT5G57655	173.7901566	620.6921761	–1.8365	2.09E–48

AT5G05600	69.43279878	248.6727862	–1.8406	1.19E–19
AT2G15890	54.35279052	195.3655608	–1.8458	1.39E–15
AT5G13740	45.84137481	165.1534198	–1.8491	2.85E–13
AT1G36370	69.89537572	252.5376778	–1.8532	3.69E–20
AT3G46930	30.48382038	110.2908112	–1.8552	4.53E–09
AT3G54130	17.06908911	61.79113394	–1.856	2.45E–05
AT2G38290	22.66627009	82.15251446	–1.8578	6.52E–07
AT1G77680	27.52332796	99.78019111	–1.8581	2.88E–08
AT2G24500	66.84236791	242.4983861	–1.8591	1.82E–19
AT1G29760	29.46615111	107.368576	–1.8654	6.50E–09
AT5G17460	50.65217499	184.8078079	–1.8673	5.00E–15
AT4G28240	29.23486264	106.7558493	–1.8686	6.91E–09
AT5G47040	72.3932912	264.7922124	–1.8709	2.02E–21
AT3G10960	18.87313917	69.14385468	–1.8733	5.66E–06
AT1G21130	17.67043913	65.18469736	–1.8832	1.05E–05
AT3G03640	36.589836	135.1298101	–1.8848	2.98E–11

AT3G22890	317.2352658	1173.418818	–1.8871	2.51E–95
AT4G01700	19.05816995	70.51070662	–1.8874	3.80E–06
AT2G35170	8.280127236	30.63633643	–1.8875	0.0049686
AT5G54170	25.62676251	95.06690859	–1.8913	4.15E–08
AT3G02840	30.90013963	114.8155624	–1.8936	1.07E–09
AT1G17180	32.19535506	119.764509	–1.8953	4.15E–10
AT4G27020	28.44848184	106.4730523	–1.9041	4.17E–09
AT3G51730	120.7325815	452.8521853	–1.9072	1.75E–37
AT3G25900	19.79829306	74.28133264	–1.9076	1.53E–06
AT2G43400	34.46198207	129.3324726	–1.908	5.54E–11
AT1G69890	100.1479076	375.9785472	–1.9085	3.39E–31
AT3G06780	23.22136242	87.19572676	–1.9088	1.40E–07
AT1G56660	1009.62043	3791.930059	–1.9091	0
AT1G55920	76.60274136	287.840164	–1.9098	5.42E–24
AT1G09932	16.3752237	61.60260264	–1.9115	1.55E–05
AT3G61740	9.852888834	37.09353349	–1.9125	0.0013707

AT5G16650	15.45006981	58.25617204	–1.9148	2.77E–05
AT4G13300	8.742704177	32.99297769	–1.916	0.0028358
AT3G15850	15.31129673	57.83197662	–1.9173	2.94E–05
AT2G15480	61.33770232	231.9406332	–1.9189	1.54E–19
AT5G43190	26.96823563	102.1368324	–1.9212	7.16E–09
AT3G53180	75.16875284	284.7765303	–1.9216	5.83E–24
AT1G64065	8.141354154	30.91913338	–1.9252	0.003988
AT4G35110	12.4433197	47.27422375	–1.9257	0.00019423
AT1G25275	61.43021771	234.0144775	–1.9296	7.14E–20
AT1G15030	21.23228157	81.06845948	–1.9329	3.24E–07
AT5G54940	361.7814252	1387.920306	–1.9397	1.62E–117
AT2G38870	27.06075102	103.8807469	–1.9407	3.85E–09
AT1G08570	53.7051828	206.5831732	–1.9436	9.06E–18
AT2G36950	94.64324204	364.3838722	–1.9449	4.13E–31
AT3G48390	12.58209278	48.45254439	–1.9452	0.00013608
AT5G20910	15.95890445	61.50833699	–1.9464	1.15E–05

AT2G32560	17.48540835	67.39994015	–1.9466	3.72E–06
AT2G40113	8.280127236	32.09745401	–1.9547	0.0027936
AT5G65207	64.20567935	248.9555831	–1.9551	1.58E–21
AT4G36500	98.57514603	382.4828771	–1.9561	6.57E–33
AT5G19855	7.540004131	29.2694845	–1.9568	0.0047402
AT5G11920	13.04466972	50.66778717	–1.9576	8.24E–05
AT5G20250	1134.793751	4413.70629	–1.9596	0
AT5G13820	22.85130086	88.89250847	–1.9598	5.23E–08
AT4G14930	12.30454662	47.88695048	–1.9604	0.00013608
AT5G49360	100.7955153	392.4279033	–1.961	6.89E–34
AT4G12400	43.52849011	170.0081008	–1.9656	6.08E–15
AT1G76680	285.4099723	1115.681107	–1.9668	1.84E–96
AT3G09370	9.344054199	36.57507242	–1.9687	0.0011192
AT5G54640	22.52749701	88.18551609	–1.9689	5.32E–08
AT4G15120	9.991661916	39.16737781	–1.9709	0.00067592
AT3G47950	11.05558888	43.59786338	–1.9795	0.0002726

AT2G43140	20.49215847	81.02132665	–1.9832	1.80E–07
AT5G66850	30.25253191	119.764509	–1.9851	8.35E–11
AT5G47070	10.8705581	43.0794023	–1.9866	0.00028888
AT3G07565	19.4744892	77.48636476	–1.9924	3.24E–07
AT5G20010	204.2277193	812.7584391	–1.9926	1.14E–71
AT4G32870	31.64026273	125.986042	–1.9934	2.07E–11
AT5G01410	75.86261825	303.7710589	–2.0015	4.15E–27
AT5G27610	9.529084976	38.17758848	–2.0023	0.00068637
AT5G21170	68.13758334	273.4175194	–2.0046	1.73E–24
AT1G19380	58.88604453	236.5596501	–2.0062	2.92E–21
AT3G14075	20.58467386	82.81237401	–2.0083	9.38E–08
AT3G11170	24.05400091	96.85795595	–2.0096	5.43E–09
AT1G58200	19.98332383	80.7385297	–2.0145	1.32E–07
AT5G52190	8.002581072	32.38025097	–2.0166	0.0019869
AT5G16120	52.22493659	211.6263855	–2.0187	3.08E–19
AT1G57990	88.58348412	359.4820584	–2.0208	1.81E–32

AT4G37390	10.45423886	42.46667558	–2.0222	0.00026105
AT1G76070	32.47290123	132.3489734	–2.027	2.99E–12
AT2G40420	20.16835461	82.24678011	–2.0279	8.28E–08
AT1G64400	6.892396414	28.13829669	–2.0295	0.0043595
AT4G36040	196.1788805	803.614671	–2.0343	4.68E–73
AT4G22530	10.63926963	43.59786338	–2.0349	0.00019262
AT5G40590	13.36847358	54.8154758	–2.0357	1.99E–05
AT5G21990	59.71868303	245.1378243	–2.0373	1.62E–22
AT5G51440	20.76970463	85.45181223	–2.0406	3.69E–08
AT4G39780	14.84871979	61.13127439	–2.0416	5.27E–06
AT5G65280	25.16418557	103.59795	–2.0416	8.71E–10
AT5G13700	11.79571198	48.68820851	–2.0453	6.43E–05
AT5G10695	41.2618631	170.5265619	–2.0471	6.96E–16
AT4G20830	400.6378882	1656.624543	–2.0479	6.54E–152
AT2G45180	8.973992647	37.18779914	–2.051	0.00064156
AT3G15450	774.585087	3210.263863	–2.0512	2.34E–295

AT3G28580	15.17252365	62.92232175	–2.0521	3.31E–06
AT4G34350	127.9025241	531.0455424	–2.0538	5.50E–49
AT5G51130	9.80663114	40.76989387	–2.0557	0.00030066
AT4G03430	88.7222572	369.2385532	–2.0572	3.26E–34
AT1G32870	10.9168158	45.43604357	–2.0573	0.00011496
AT3G14050	53.33512125	222.7025994	–2.062	7.71E–21
AT2G29440	16.88405833	70.51070662	–2.0622	6.22E–07
AT5G53050	9.251538811	38.7903152	–2.0679	0.00041999
AT2G27150	23.54516627	98.93180026	–2.071	1.47E–09
AT5G17850	25.90430867	108.9239592	–2.0721	1.78E–10
AT5G46710	10.63926963	44.77618401	–2.0733	0.0001186
AT5G54490	21.32479696	90.07082911	–2.0785	8.57E–09
AT3G57520	727.4947544	3076.689436	–2.0804	6.39E–289
AT5G66050	59.9037138	253.8573969	–2.0833	4.63E–24
AT5G35735	152.0952981	644.8713155	–2.084	9.93E–61
AT2G41880	16.328966	69.28525316	–2.0851	6.37E–07

AT3G03150	35.61842442	151.2492363	–2.0862	1.62E–14
AT1G32200	13.73853513	58.35043769	–2.0865	6.33E–06
AT5G56580	6.522334862	27.71410126	–2.0872	0.0037583
AT4G18250	9.667858058	41.24122212	–2.0928	0.0002189
AT5G11520	100.1941653	428.107452	–2.0952	1.10E–40
AT1G67810	22.66627009	96.85795595	–2.0953	1.61E–09
ATMG00020	14.10859669	60.33001636	–2.0963	3.82E–06
AT1G58420	6.337304086	27.14850736	–2.0989	0.0040515
AT3G17790	27.38455488	117.3136021	–2.0989	1.88E–11
AT2G19810	12.4433197	53.30722539	–2.099	1.66E–05
AT4G05120	9.714115752	41.61828472	–2.0991	0.00019492
AT1G33590	50.09708266	214.7842848	–2.1001	1.22E–20
AT2G32150	279.257699	1197.833622	–2.1008	5.10E–114
AT2G37430	7.817550296	33.5585716	–2.1019	0.0010468
AT5G41080	516.8372157	2223.491032	–2.105	3.58E–212
AT5G52750	9.529084976	41.09982364	–2.1087	0.00020556

AT5G47740	28.49473954	123.1109396	–2.1112	4.34E–12
AT4G38060	35.29462056	152.9931509	–2.1159	5.65E–15
AT5G03030	15.45006981	66.97574472	–2.116	7.63E–07
AT4G04610	121.1951584	525.483869	–2.1163	1.34E–50
AT5G64310	119.1598199	517.7540857	–2.1194	5.78E–50
AT4G36790	22.15743545	96.38662769	–2.121	1.25E–09
AT4G39660	223.8872392	976.5921398	–2.125	1.59E–94
AT3G11580	7.077427191	30.91913338	–2.1272	0.0016248
AT3G06420	29.92872805	131.2649184	–2.1329	4.81E–13
AT3G62770	32.05658198	140.7386163	–2.1343	5.79E–14
AT1G02660	45.51757095	200.173109	–2.1368	9.75E–20
AT3G10020	104.2185847	458.5552571	–2.1375	8.09E–45
AT1G62975	27.10700872	119.3403136	–2.1383	6.10E–12
AT3G55840	12.07325815	53.54288952	–2.1489	1.08E–05
AT5G62520	56.75819061	251.7835526	–2.1493	6.27E–25
AT2G16900	57.68334449	256.1197726	–2.1506	2.24E–25

AT2G15695	25.58050481	113.5901089	–2.1507	1.77E–11
AT5G24490	35.75719751	158.9318868	–2.1521	6.68E–16
AT5G42940	52.78002892	235.0513997	–2.1549	2.19E–23
AT5G28610	41.12309002	183.6766201	–2.1591	2.11E–18
AT1G10370	41.53940926	186.1746598	–2.1641	1.04E–18
AT5G64410	27.29203949	123.6765335	–2.18	1.09E–12
AT1G78895	18.08675838	82.10538163	–2.1825	1.33E–08
AT2G46980	5.643438675	25.64025695	–2.1838	0.0040725
AT5G60800	10.68552733	48.64107569	–2.1865	2.41E–05
AT1G29640	11.51816582	52.55310018	–2.1899	9.82E–06
AT1G22770	27.29203949	125.2790496	–2.1986	5.42E–13
AT5G58650	11.56442351	53.11869409	–2.1995	8.04E–06
AT5G23510	28.44848184	130.6993245	–2.1998	1.53E–13
AT5G42380	84.23526088	389.5056681	–2.2091	6.75E–40
AT5G47860	12.25828892	56.70078881	–2.2096	3.27E–06
AT2G33150	200.480846	928.233861	–2.211	5.43E–95

AT3G17690	10.63926963	49.34806807	–2.2136	1.71E–05
AT2G20562	6.753623332	31.39046164	–2.2166	0.0009889
AT3G22370	50.05082497	232.9304225	–2.2184	4.02E–24
AT3G19210	6.984911802	32.75731357	–2.2295	0.00068465
AT3G53620	65.63966787	308.1544117	–2.231	4.77E–32
AT4G01010	5.13460404	24.13200654	–2.2326	0.0048284
AT1G02610	6.106015615	28.70389059	–2.2329	0.0016938
AT3G61060	62.95672161	296.0884084	–2.2336	7.38E–31
AT3G01650	33.30553972	156.7637769	–2.2348	1.62E–16
AT1G29400	84.00397241	396.7641232	–2.2398	2.07E–41
AT5G67340	10.31546577	49.01813829	–2.2485	1.44E–05
AT5G23350	26.87572025	127.871355	–2.2503	1.15E–13
AT2G43500	5.13460404	24.46193632	–2.2522	0.0041688
AT5G44260	5.180861734	24.69760045	–2.2531	0.0039449
AT4G20070	35.10958979	167.4157954	–2.2535	8.28E–18
AT3G47420	43.38971702	207.5729625	–2.2582	4.65E–22

AT2G35390	17.53166605	83.99069465	–2.2603	3.40E–09
AT3G04320	13.59976205	65.32609584	–2.2641	2.72E–07
AT5G22290	14.15485438	68.0597997	–2.2655	1.41E–07
AT5G06720	5.273377122	25.35746	–2.2656	0.0032225
AT2G41190	8.927734953	43.03226948	–2.2691	5.12E–05
AT5G63790	163.9372677	791.1716051	–2.2708	5.62E–84
AT5G38200	15.26503904	73.71573873	–2.2717	3.47E–08
AT1G01470	212.1377849	1024.761887	–2.2722	7.77E–109
AT1G72330	10.40798116	50.29072457	–2.2726	9.02E–06
AT2G29500	9.760373446	47.1799581	–2.2732	1.87E–05
AT1G16850	5.689696369	27.66696844	–2.2817	0.0017801
AT1G58180	43.01965547	210.5894633	–2.2914	8.07E–23
AT4G03960	11.33313504	55.569601	–2.2937	2.17E–06
AT1G60730	51.53107118	252.9147404	–2.2951	2.17E–27
AT2G42270	61.75402157	303.2525978	–2.2959	8.81E–33
AT1G14540	16.51399678	81.39838925	–2.3013	3.90E–09

AT5G15870	31.59400504	156.0096517	–2.3039	4.07E–17
AT3G12580	275.2332796	1359.970541	–2.3048	3.51E–147
AT1G19530	148.4871979	733.7638239	–2.305	1.74E–79
AT3G26910	30.85388194	152.7574867	–2.3077	8.31E–17
AT5G04040	54.90788284	272.2863316	–2.31	1.01E–29
AT5G28630	48.47806337	241.2729326	–2.3153	1.92E–26
AT5G28050	179.2485645	897.3147276	–2.3237	3.07E–98
AT5G02160	7.493746437	37.51772892	–2.3238	0.00013951
AT3G13520	104.1260693	522.7501652	–2.3278	2.07E–57
AT2G32120	7.678777213	38.6017839	–2.3297	0.00010421
AT4G39230	14.66368902	73.71573873	–2.3297	1.88E–08
AT3G13080	50.05082497	252.4434122	–2.3345	5.75E–28
AT3G27650	4.764542488	24.03774089	–2.3349	0.0034697
AT1G60750	11.7031966	59.15169572	–2.3375	6.33E–07
AT3G53650	7.493746437	38.03619	–2.3436	0.00011111
AT5G45350	50.83720577	258.8534764	–2.3482	6.89E–29

AT5G02970	29.28112034	149.2696577	–2.3499	7.99E–17
AT5G42900	10.31546577	52.60023301	–2.3503	2.91E–06
AT3G54150	11.88822737	60.80134461	–2.3546	3.64E–07
AT4G38940	16.19019292	82.85950684	–2.3555	1.44E–09
AT3G16330	8.187611848	42.56094123	–2.378	2.98E–05
AT1G33600	7.308715661	38.08332282	–2.3815	9.00E–05
AT1G61360	32.33412815	168.6412488	–2.3828	2.60E–19
AT2G38400	96.81735366	505.9237465	–2.3856	2.12E–57
AT4G19870	5.088346346	26.63004628	–2.3878	0.0015316
AT5G66700	6.984911802	36.66933807	–2.3923	0.0001213
AT3G47040	4.255707853	22.43522483	–2.3983	0.0042235
AT5G01810	157.5999636	832.6013585	–2.4014	3.18E–95
AT3G19970	19.19694303	102.0896996	–2.4109	5.05E–12
AT2G30140	11.51816582	61.31980569	–2.4124	1.95E–07
AT3G23920	75.53881439	402.3257966	–2.4131	2.31E–46
AT5G02230	41.49315157	221.6656773	–2.4174	9.70E–26

AT2G17880	11.79571198	63.06372022	–2.4185	1.19E–07
AT5G50450	4.949573264	26.53578063	–2.4226	0.0013849
AT1G30040	5.504665593	29.74081275	–2.4337	0.00058695
AT2G20500	5.273377122	28.70389059	–2.4444	0.00073254
AT3G45300	117.8646045	641.9962131	–2.4454	3.53E–75
AT5G54080	40.42922461	220.2988253	–2.446	5.80E–26
AT1G77000	12.21203123	66.55154929	–2.4462	3.72E–08
AT5G51260	4.857057876	26.72431193	–2.46	0.0011534
AT3G18950	4.90331557	27.05424171	–2.464	0.0010427
AT2G47550	24.74786632	136.8265918	–2.467	1.92E–16
AT5G18630	35.57216673	197.4865379	–2.4729	1.22E–23
AT5G25250	13.27595819	73.71573873	–2.4732	4.24E–09
AT1G55510	29.74369728	165.6718809	–2.4777	6.11E–20
AT3G60450	58.97855992	329.5055815	–2.482	2.03E–39
AT1G70670	36.03474367	202.1526876	–2.488	2.24E–24
AT5G65110	93.20925352	523.881353	–2.4907	7.76E–63

AT1G80380	290.5908341	1634.849178	–2.4921	5.95E–196
AT3G50980	5.458407899	30.77773491	–2.4953	0.00034643
AT5G15120	4.995830958	28.18542952	–2.4962	0.00068784
AT1G11210	22.75878548	129.0968084	–2.504	7.96E–16
AT1G72900	30.94639732	175.6640398	–2.505	2.08E–21
AT5G61560	5.180861734	29.4580158	–2.5074	0.00046865
AT1G68290	3.978161689	22.62375613	–2.5077	0.002878
AT1G13480	9.020250341	51.56331085	–2.5151	1.16E–06
AT3G13650	20.39964308	116.6537426	–2.5156	2.01E–14
AT1G65390	4.440738629	25.45172565	–2.5189	0.001311
AT4G18950	118.0033775	679.8910046	–2.5265	5.48E–83
AT3G51890	24.79412401	143.2837888	–2.5308	9.41E–18
AT4G18510	11.28687735	65.37322866	–2.5341	2.31E–08
AT3G03990	74.33611435	431.07682	–2.5358	6.75E–53
AT2G38820	13.2297005	76.77937238	–2.5369	9.59E–10
AT1G12950	18.73436609	108.971092	–2.5402	1.18E–13

AT1G13990	17.71669682	103.1266217	–2.5412	5.91E–13
AT5G38940	13.92356591	81.16272513	–2.5433	2.66E–10
AT5G02810	18.08675838	105.4361301	–2.5434	3.01E–13
AT1G03905	17.62418144	102.9852232	–2.5468	5.68E–13
AT2G38250	10.50049655	61.41407134	–2.5481	6.07E–08
AT4G23550	11.24061966	65.79742409	–2.5493	1.79E–08
AT1G71695	15.08000826	88.27978175	–2.5494	3.39E–11
AT1G20630	30.66885116	179.8117284	–2.5516	2.06E–22
AT2G17280	12.85963895	75.45965327	–2.5529	1.18E–09
AT4G19720	8.233869542	48.40541156	–2.5555	2.11E–06
AT2G22880	30.94639732	182.074104	–2.5567	9.47E–23
AT1G76600	54.4453059	322.624189	–2.567	2.96E–40
AT3G02550	14.38614285	85.49894505	–2.5712	5.71E–11
AT1G04310	15.35755443	91.43768104	–2.5738	1.02E–11
AT1G74360	13.50724666	80.50286557	–2.5753	2.25E–10
AT5G02320	7.447488743	44.39912141	–2.5757	5.67E–06

AT4G05320	1031.824124	6167.094523	–2.5794	0
AT5G02580	6.707365638	40.25143279	–2.5852	1.71E–05
AT5G08790	159.7278176	961.9809639	–2.5904	4.33E–121
AT1G79360	3.931903995	23.80207677	–2.5978	0.0015846
AT1G05060	7.632519519	46.28443442	–2.6003	2.86E–06
AT1G27760	114.9503697	698.3670721	–2.603	1.87E–88
AT5G40780	34.60075515	210.7308618	–2.6065	6.83E–27
AT1G28190	9.667858058	58.9160316	–2.6074	7.58E–08
AT1G61820	110.3246003	672.8682137	–2.6086	1.93E–85
AT5G16370	114.7190813	700.5351821	–2.6104	4.90E–89
AT3G13450	26.78320486	164.3521617	–2.6174	3.70E–21
AT1G28760	6.661107944	41.47688624	–2.6385	9.06E–06
AT4G37030	4.6720271	29.12808602	–2.6403	0.00030574
AT2G02230	25.02541248	156.3395814	–2.6432	2.22E–20
AT3G44860	3.376811666	21.11550572	–2.6446	0.0029318
AT3G08970	4.255707853	26.77144476	–2.6532	0.00057479

AT3G05390	6.152273309	38.74318238	–2.6548	1.82E–05
AT4G15530	53.38137894	338.6022168	–2.6652	3.29E–44
AT1G76700	35.75719751	226.9916865	–2.6663	9.60E–30
AT1G25400	150.0137018	956.9377516	–2.6733	3.74E–125
AT4G12120	11.42565043	73.29154331	–2.6814	6.06E–10
AT3G55940	8.372642624	53.87281929	–2.6858	1.81E–07
AT1G67070	6.892396414	44.35198859	–2.6859	3.00E–06
AT3G03110	19.89080844	129.4267382	–2.702	2.22E–17
AT3G29240	46.76652869	305.2793093	–2.7066	1.29E–40
AT1G68410	74.24359896	487.6362103	–2.7155	5.04E–65
AT3G19930	93.48679969	614.6120416	–2.7168	5.70E–82
AT5G54710	3.053007808	20.07858357	–2.7174	0.0032555
AT5G63130	8.048838766	53.02442844	–2.7198	1.84E–07
AT1G54100	221.7593853	1462.107373	–2.721	3.68E–195
AT2G23120	347.5803131	2295.321458	–2.7233	0
AT1G17300	7.956323378	52.55310018	–2.7236	2.06E–07

AT5G16970	21.27853927	140.6443506	–2.7246	4.83E–19
AT5G50720	7.910065684	52.60023301	–2.7333	1.91E–07
AT4G03510	27.75461643	184.7135423	–2.7345	5.46E–25
AT5G52760	3.746873219	25.02753022	–2.7398	0.00071483
AT1G53920	7.123684885	47.65128636	–2.7418	7.96E–07
AT5G51830	42.14075929	283.0797486	–2.7479	2.35E–38
AT1G67480	217.272389	1462.39017	–2.7508	1.13E–197
AT4G15130	10.63926963	72.44315245	–2.7675	3.46E–10
AT1G80920	161.7168984	1107.338597	–2.7756	2.83E–151
AT4G30430	3.238038584	22.19956071	–2.7773	0.0014868
AT1G30755	23.54516627	161.4770594	–2.7778	2.71E–22
AT4G02520	164.2148139	1126.663055	–2.7784	4.31E–154
AT5G27760	47.22910563	329.7883785	–2.8038	1.09E–45
AT3G17770	50.92972116	357.7381439	–2.8123	1.03E–49
AT1G68390	3.376811666	23.75494394	–2.8145	0.00083439
AT1G15010	32.98173586	232.8361569	–2.8196	1.56E–32

AT3G06500	75.72384517	535.0046997	–2.8207	1.57E–74
AT3G13430	27.61584335	195.5069593	–2.8237	2.03E–27
AT2G45170	48.75560954	347.3217895	–2.8326	1.15E–48
AT1G64660	160.9767753	1150.417999	–2.8372	3.83E–161
AT1G09460	32.33412815	231.8935004	–2.8423	1.07E–32
AT1G03090	101.7206692	729.8989323	–2.8431	1.43E–102
AT2G47190	2.914234726	20.92697442	–2.8442	0.0018287
AT5G57660	7.540004131	54.15561624	–2.8445	5.56E–08
AT5G64510	2.821719337	20.45564617	–2.8579	0.0020413
AT3G59480	4.949573264	35.96234569	–2.8611	1.59E–05
AT1G23060	2.636688561	19.23019271	–2.8666	0.002924
AT5G04250	8.696446483	63.53504847	–2.8691	2.34E–09
AT3G12920	31.03891271	227.0859522	–2.8711	2.16E–32
AT5G62350	98.29759987	719.1997809	–2.8712	3.40E–102
AT5G03545	122.8141777	903.7719247	–2.8795	8.36E–129
AT3G60930	4.810800182	35.44388461	–2.8812	1.71E–05

AT4G01026	4.301965547	32.14458684	–2.9015	4.49E–05
AT1G80820	31.96406659	239.3876196	–2.9048	1.42E–34
AT1G19540	3.885646301	29.12808602	–2.9062	0.00011515
AT2G38210	2.590430867	19.46585684	–2.9097	0.0024533
AT1G11260	128.3188433	970.040677	–2.9183	2.84E–140
AT5G25450	4.394480935	33.22864182	–2.9187	2.95E–05
AT3G45060	4.163192465	31.53186011	–2.921	5.06E–05
AT5G52710	5.458407899	41.47688624	–2.9258	1.99E–06
AT1G14640	3.006750114	23.04795156	–2.9384	0.0007306
AT1G78830	65.22334862	502.6244488	–2.946	1.65E–73
AT1G80670	23.86897013	184.2893468	–2.9488	3.96E–27
AT4G38420	5.412150205	42.46667558	–2.9721	1.13E–06
AT2G26380	3.099265502	24.41480349	–2.9778	0.0004186
AT2G45760	2.914234726	23.04795156	–2.9834	0.0006459
AT5G64250	92.14532656	728.8148773	–2.9836	5.32E–108
AT1G04100	5.550923287	44.11632446	–2.9905	5.94E–07

AT2G17840	315.1999273	2505.580992	–2.9908	0
AT3G19250	2.451657785	19.79578662	–3.0134	0.0017384
AT4G29190	9.436569587	76.21377847	–3.0137	1.03E–11
AT2G16660	27.89338952	225.3891705	–3.0144	6.85E–34
AT3G43190	8.002581072	65.09043171	–3.0239	4.17E–10
AT4G36430	24.51657785	200.5030387	–3.0318	2.25E–30
AT4G24380	36.12725906	296.1355412	–3.0351	8.83E–45
AT4G34030	59.07107531	487.6362103	–3.0453	6.84E–74
AT2G35980	13.46098897	113.3544448	–3.074	1.42E–17
AT2G34790	11.88822737	101.0056446	–3.0868	9.11E–16
AT2G40880	4.718284794	40.15716714	–3.0893	1.39E–06
AT5G08350	57.68334449	494.3762043	–3.0994	2.72E–76
AT5G16010	21.60234312	185.5148003	–3.1023	8.37E–29
AT5G15970	80.76593382	697.0944859	–3.1095	6.71E–108
AT5G33355	71.79194118	619.7966524	–3.1099	5.79E–96
AT1G07040	21.0472508	183.0638934	–3.1206	1.34E–28

AT5G48180	114.7190813	998.650302	–3.1219	4.38E–155
AT5G20830	35.29462056	307.8244819	–3.1246	5.97E–48
AT1G67850	5.597180981	49.30093524	–3.1388	4.54E–08
AT5G48430	23.08258933	203.4724067	–3.14	6.05E–32
AT3G50970	231.4735011	2048.251188	–3.1455	0
AT4G01870	93.30176891	826.2855599	–3.1467	1.94E–129
AT5G57500	2.174111621	19.27732554	–3.1484	0.0015316
AT1G68500	4.995830958	44.49338706	–3.1548	2.25E–07
AT4G15610	172.0786219	1532.665212	–3.1549	1.26E–240
AT5G49700	6.291046392	56.04092926	–3.1551	3.81E–09
AT2G16060	7.031169497	62.92232175	–3.1617	3.18E–10
AT3G15356	5.689696369	51.2805139	–3.172	1.87E–08
AT5G65690	122.7216623	1108.846847	–3.1756	3.41E–175
AT3G49160	6.753623332	61.08414156	–3.1771	5.49E–10
AT2G30400	5.088346346	46.56723137	–3.194	8.85E–08
AT4G24960	31.82529351	291.3751259	–3.1946	2.01E–46

AT1G30760	17.30037758	161.0999968	–3.2191	5.47E–26
AT2G29460	4.949573264	46.6143642	–3.2354	7.03E–08
AT1G49570	5.227119428	49.48946654	–3.243	2.40E–08
AT4G02890	124.9882893	1190.198104	–3.2513	1.67E–192
AT3G02800	11.98074276	114.8155624	–3.2605	7.30E–19
AT1G22400	12.25828892	117.6435319	–3.2626	2.48E–19
AT4G18280	31.96406659	306.7875597	–3.2627	6.85E–50
AT2G31945	4.440738629	42.65520688	–3.2639	2.56E–07
AT5G06730	3.885646301	37.37633045	–3.2659	1.74E–06
AT1G21400	45.05499401	437.2040873	–3.2785	2.23E–71
AT3G14770	22.52749701	219.2147704	–3.2826	6.27E–36
AT4G36880	2.035338538	19.93718509	–3.2921	0.00089755
AT1G14890	15.03375057	149.175392	–3.3107	9.53E–25
AT1G05340	33.53682819	335.5385832	–3.3227	1.65E–55
AT5G39720	2.497915479	25.02753022	–3.3247	0.00013037
AT5G13210	11.00933119	110.9978035	–3.3337	1.24E–18

AT2G32020	14.84871979	149.8352516	–3.335	5.03E–25
AT4G24230	88.67599951	903.3005965	–3.3486	2.77E–150
AT2G30395	2.867977031	29.22235167	–3.349	2.59E–05
AT5G52300	1.572761598	16.16655907	–3.3616	0.0030877
AT1G32970	1.757792374	18.09900491	–3.3641	0.0015202
AT5G10625	23.26762011	240.3302761	–3.3686	2.48E–40
AT3G06850	112.8687735	1172.146232	–3.3764	1.74E–196
AT5G42830	5.458407899	56.70078881	–3.3768	7.70E–10
AT3G11020	3.376811666	35.11395483	–3.3783	2.61E–06
AT2G03760	11.51816582	119.8116419	–3.3788	2.49E–20
AT1G23550	2.96049242	30.82486773	–3.3802	1.30E–05
AT1G07150	11.14810427	116.6066097	–3.3868	7.70E–20
AT1G75860	27.15326641	284.3523349	–3.3885	6.21E–48
AT4G33070	10.130435	106.1902554	–3.3899	4.11E–18
AT2G05580	3.006750114	31.53186011	–3.3905	9.67E–06
AT3G23550	2.821719337	29.6465471	–3.3932	1.93E–05

AT4G28290	2.914234726	30.77773491	–3.4007	1.24E–05
AT3G15500	14.61743132	154.9255967	–3.4058	2.29E–26
AT4G35480	15.5888429	165.2948182	–3.4065	4.01E–28
AT3G08860	15.5888429	165.624748	–3.4093	3.38E–28
AT5G20150	63.37304086	674.2821984	–3.4114	3.43E–114
AT4G17090	8.973992647	96.29236204	–3.4236	1.32E–16
AT5G41610	4.255707853	46.14303595	–3.4386	3.13E–08
AT3G19240	156.8598405	1704.322962	–3.4417	6.52E–291
AT1G15040	124.3406816	1361.573057	–3.4529	5.23E–233
AT5G40880	9.482827282	104.5406065	–3.4626	3.55E–18
AT1G21790	4.810800182	53.40149104	–3.4725	1.59E–09
AT5G28145	2.867977031	31.86178989	–3.4737	6.51E–06
AT5G13080	16.60651217	184.6192766	–3.4747	6.27E–32
AT1G06570	34.46198207	383.1427367	–3.4748	4.96E–66
AT5G28646	2.174111621	24.36767067	–3.4865	0.00011156
AT3G06490	3.885646301	43.92779316	–3.4989	5.58E–08

AT3G48450	16.00516214	181.0371819	–3.4997	1.64E–31
AT2G18700	65.13083323	740.9280134	–3.5079	1.18E–128
AT1G74010	5.458407899	62.21532937	–3.5107	4.08E–11
AT1G14260	2.220369315	25.35746	–3.5135	7.17E–05
AT3G09350	27.89338952	319.7019539	–3.5187	1.01E–55
AT1G26380	1.989080844	22.81228743	–3.5196	0.00018779
AT5G04120	1.572761598	18.05187208	–3.5208	0.0011722
AT4G36410	9.945404222	114.5798983	–3.5262	3.32E–20
AT5G40010	2.867977031	33.22864182	–3.5343	3.17E–06
AT3G22120	1.71153468	19.84291944	–3.5353	0.00057479
AT1G66050	1.433988516	16.68502015	–3.5404	0.0019125
AT1G10070	52.36370967	612.5853301	–3.5483	1.88E–107
AT4G28085	15.03375057	176.418165	–3.5527	4.07E–31
AT2G46240	18.22553146	214.1244252	–3.5544	1.00E–37
AT3G20670	20.53841616	245.5148869	–3.5794	1.78E–43
AT5G39610	5.828469451	69.94511271	–3.585	1.16E–12

AT3G01970	7.447488743	89.8822978	–3.5932	3.49E–16
AT2G47950	1.989080844	24.08487372	–3.598	9.65E–05
AT3G26740	6.059757921	73.81000439	–3.6065	2.13E–13
AT5G64530	2.682946255	32.85157922	–3.6141	2.86E–06
AT3G09405	3.238038584	39.92150301	–3.624	1.65E–07
AT3G16150	33.53682819	417.1255037	–3.6367	1.00E–74
AT1G76410	9.57534267	121.131361	–3.6611	5.14E–22
AT2G43120	62.49414467	802.3420847	–3.6824	4.41E–145
AT4G27310	1.387730822	17.81620796	–3.6824	0.0009857
AT5G61390	3.238038584	41.99534732	–3.697	5.44E–08
AT5G55090	1.94282315	25.35746	–3.7062	4.57E–05
AT5G67080	3.053007808	39.92150301	–3.7089	1.22E–07
AT2G04040	2.821719337	36.90500219	–3.7092	4.13E–07
AT1G43670	29.14234726	381.9644161	–3.7122	1.31E–69
AT1G19250	1.48024621	19.46585684	–3.717	0.000481
AT5G57510	6.38356178	84.22635877	–3.7218	1.27E–15

AT3G19390	166.5739563	2252.383454	–3.7572	0
AT3G05630	3.700615525	50.24359175	–3.7631	1.39E–09
AT5G43450	2.266627009	30.87200056	–3.7677	4.14E–06
AT1G67980	6.984911802	95.58536966	–3.7745	7.22E–18
AT5G57220	9.57534267	131.8776451	–3.7837	1.46E–24
AT3G17110	29.88247036	415.0516594	–3.7959	5.98E–77
AT5G45630	2.729203949	37.94192435	–3.7972	2.03E–07
AT2G24850	2.035338538	28.32682799	–3.7988	1.10E–05
AT3G29670	1.387730822	19.51298967	–3.8136	0.00040085
AT1G10160	9.344054199	132.3961062	–3.8247	7.39E–25
AT5G48570	14.29362746	204.3679304	–3.8377	2.63E–38
AT5G52050	50.51340191	723.7245321	–3.8407	2.70E–135
AT3G02040	29.88247036	429.4743039	–3.8452	2.05E–80
AT1G74310	50.37462883	725.4684467	–3.8481	8.41E–136
AT5G65600	1.24895774	18.38180186	–3.8795	0.00058182
AT4G36010	13.73853513	203.6138052	–3.8895	1.53E–38

AT5G54165	1.341473128	20.17284922	–3.9105	0.00026142
AT4G22710	27.10700872	409.3957204	–3.9168	1.06E–77
AT5G07010	15.40381212	233.5431493	–3.9223	1.91E–44
AT1G43160	35.38713595	536.7486143	–3.9229	5.63E–102
AT5G14470	6.059757921	92.23893907	–3.928	9.06E–18
AT4G36820	2.174111621	33.13437617	–3.9298	1.04E–06
AT2G43510	1.896565456	28.93955472	–3.9316	6.25E–06
AT5G40000	6.753623332	103.7864813	–3.9418	5.30E–20
AT1G77380	10.130435	156.999441	–3.954	3.84E–30
AT1G05560	1.063926963	16.54362167	–3.9588	0.0011243
AT4G24040	2.312884703	36.10374416	–3.9644	2.68E–07
AT2G18193	15.68135829	247.6829968	–3.9814	1.28E–47
AT5G52640	54.21401743	866.207063	–3.998	1.35E–166
AT1G59860	2.220369315	35.63241591	–4.0043	2.94E–07
AT4G22690	29.92872805	484.6668423	–4.0174	1.17E–93
AT1G56060	1.063926963	17.39201253	–4.031	0.00071729

AT2G41100	158.4788598	2596.31168	–4.0341	0
AT2G34390	1.989080844	32.61591509	–4.0354	1.00E–06
AT4G20730	3.284296278	54.01421777	–4.0397	8.72E–11
AT2G32210	4.579511712	76.02524717	–4.0532	4.88E–15
AT3G03660	1.619019292	27.00710888	–4.0601	1.09E–05
AT1G08050	11.33313504	189.6153561	–4.0645	4.93E–37
AT1G08090	3.053007808	51.6575765	–4.0807	2.12E–10
AT5G38430	1.24895774	21.20977138	–4.0859	0.00012877
AT1G22985	6.152273309	105.7189271	–4.103	6.39E–21
AT2G24130	4.718284794	81.58692056	–4.112	2.97E–16
AT1G54050	2.544173173	44.21059011	–4.1191	4.94E–09
AT1G08630	13.46098897	234.0144775	–4.1197	4.72E–46
AT4G28350	4.440738629	77.39209911	–4.1233	1.82E–15
AT5G38410	3.700615525	64.94903323	–4.1335	4.57E–13
AT4G35770	42.0019862	743.2375218	–4.1453	1.69E–146
AT4G16690	0	14.84683996	–4.1563	0.0018603

AT1G63180	19.19694303	349.3013681	–4.1855	2.29E–69
AT5G66400	0	15.22390256	–4.1925	0.0015247
AT5G49620	2.544173173	46.52009855	–4.1926	1.42E–09
AT1G69680	9.390311893	174.1557894	–4.2131	8.02E–35
AT4G33666	2.405400091	44.72905119	–4.2169	2.93E–09
AT1G72800	4.255707853	82.10538163	–4.27	9.76E–17
AT5G20790	1.94282315	37.61199457	–4.275	6.24E–08
AT1G70130	1.989080844	39.02597933	–4.2943	3.17E–08
AT1G15330	6.291046392	124.4306587	–4.3059	2.68E–25
AT4G01360	17.34663527	345.3422108	–4.3153	7.47E–70
AT1G47510	1.341473128	26.91284323	–4.3264	7.08E–06
AT3G46080	3.238038584	65.18469736	–4.3313	1.78E–13
AT5G24600	2.174111621	43.88066033	–4.3351	3.10E–09
AT2G38823	0	13.15005825	–4.3438	0.0033196
AT3G16530	6.799881026	141.3042102	–4.3772	5.94E–29
AT1G02920	26.69068947	560.3150269	–4.3918	2.24E–114

AT2G32190	8.141354154	171.5163512	–4.3969	3.69E–35
AT3G22740	3.793130913	79.93727167	–4.3974	1.42E–16
AT2G22860	37.51498988	797.5345365	–4.41	4.78E–163
AT2G44578	2.867977031	61.13127439	–4.4138	8.49E–13
AT2G01340	3.700615525	79.46594342	–4.4245	1.55E–16
AT2G18550	1.295215434	27.85549974	–4.4267	3.92E–06
AT3G03670	1.48024621	32.00318836	–4.4343	5.75E–07
AT1G21120	15.49632751	335.9156458	–4.4381	4.99E–69
AT4G35180	1.665276986	36.10374416	–4.4383	8.71E–08
AT5G06980	1.572761598	35.53815026	–4.498	1.01E–07
AT5G46330	0	17.95760643	–4.5132	0.00032877
AT4G18170	1.665276986	39.49730758	–4.5679	1.38E–08
AT5G20230	47.41413641	1125.861797	–4.5696	1.84E–234
AT1G63040	1.48024621	35.49101743	–4.5835	8.81E–08
AT1G05680	7.308715661	176.6538291	–4.5952	4.93E–37
AT3G60140	25.62676251	620.7864417	–4.5984	9.62E–130

AT2G17660	6.013500227	145.9232271	–4.6009	1.17E–30
AT5G14740	0	20.31424769	–4.6087	0.00010152
AT1G10170	66.42604866	1628.533379	–4.6157	0
AT1G26240	2.266627009	55.61673383	–4.6169	6.08E–12
AT3G12510	3.42306936	84.8390855	–4.6314	5.01E–18
AT5G59080	4.163192465	103.315153	–4.6332	7.04E–22
AT5G02020	5.550923287	137.8635139	–4.6344	4.41E–29
AT1G74000	1.850307762	46.33156725	–4.6462	4.69E–10
AT5G43370	0	19.93718509	–4.6641	0.00011487
AT5G43360	0	13.4328552	–4.7477	0.0022365
AT4G34380	1.804050068	48.64107569	–4.7529	1.24E–10
AT1G01480	1.341473128	36.24514264	–4.7559	4.66E–08
AT3G47380	2.220369315	60.56568048	–4.7696	3.78E–13
AT3G46090	1.295215434	36.99926784	–4.8362	2.90E–08
AT5G52670	0	27.05424171	–4.87	3.25E–06
AT5G39580	28.44848184	837.3146411	–4.8793	2.40E–179

AT1G71520	1.48024621	44.16345729	–4.8989	8.24E–10
AT5G52310	39.18026687	1179.734617	–4.9122	2.15E–253
AT1G56250	3.469327054	107.2271775	–4.9499	2.64E–23
AT4G16680	8.09509646	253.8573969	–4.9708	7.55E–55
AT5G01380	5.597180981	175.8997039	–4.9739	4.35E–38
AT3G14210	0	13.2443239	–4.9915	0.0021693
AT3G28210	16.23645061	517.1413589	–4.9933	8.96E–112
AT5G16980	0	11.9246048	–5.01	0.0040473
AT2G02990	0	19.79578662	–5.0408	9.29E–05
AT3G16120	0	29.97647688	–5.092	6.39E–07
AT3G47340	76.37145289	2625.298368	–5.1033	0
AT5G62360	3.284296278	113.6372417	–5.1127	6.31E–25
Novel00001	1.804050068	62.73379044	–5.1199	6.22E–14
AT4G37370	4.116934771	144.0850469	–5.1292	1.52E–31
AT1G16030	8.002581072	285.3892571	–5.1563	2.36E–62
AT2G39400	4.440738629	159.4032151	–5.1657	6.19E–35

AT4G12735	0	23.75494394	–5.197	1.25E–05
AT3G28600	0	23.09508439	–5.2632	1.66E–05
AT5G52400	0	12.53733152	–5.275	0.0027639
AT2G10940	0	19.46585684	–5.2829	9.64E–05
AT2G36770	0	19.74865379	–5.3037	8.35E–05
AT4G37710	0	22.05816223	–5.3124	2.68E–05
AT1G59660	11.93448507	475.2874101	–5.3156	1.50E–104
AT5G38420	1.202700045	48.21688026	–5.3252	6.43E–11
AT1G73010	9.112765729	371.7837258	–5.3504	6.44E–82
AT5G26340	17.67043913	749.7889845	–5.4071	2.58E–165
AT1G63530	4.348223241	188.9554965	–5.4415	7.72E–42
AT3G60420	0	22.67088896	–5.4775	1.86E–05
AT5G26000	0	22.34095918	–5.4816	2.18E–05
AT1G73220	0	17.10921558	–5.5309	0.0002811
AT1G77120	12.81338125	611.548408	–5.5767	1.45E–135
AT1G64160	2.544173173	131.5005825	–5.6917	2.22E–29

AT5G39120	0	26.48864781	–5.702	2.65E–06
AT5G01760	0	12.44306587	–5.7495	0.0026768
AT2G41730	0	51.6575765	–5.8031	8.63E–12
AT2G40340	3.238038584	183.4880888	–5.8244	6.21E–41
AT1G02930	9.945404222	575.2561325	–5.854	4.92E–128
AT1G80160	4.070677077	240.9901356	–5.8876	1.05E–53
AT3G01500	0	38.83744803	–5.9062	5.37E–09
AT1G29395	0	27.85549974	–5.9121	1.30E–06
AT1G66090	1.341473128	83.66076487	–5.9627	7.43E–19
AT2G43620	0	41.00555799	–5.9846	1.82E–09
AT5G06760	0	46.94429398	–5.987	9.18E–11
AT2G36800	0	15.36530104	–6.0538	0.00064022
AT4G06746	2.405400091	161.0057311	–6.0647	6.08E–36
AT1G14550	0	21.77536528	–6.0714	2.71E–05
AT5G22300	1.017669269	69.09672186	–6.0853	1.26E–15
AT1G07400	1.94282315	136.4495292	–6.1341	1.73E–30

AT1G69490	6.059757921	429.2857726	–6.1465	1.89E–95
AT3G12320	0	16.4022232	–6.1481	0.00038711
AT5G49690	0	49.77226349	–6.2641	2.39E–11
AT1G69930	0	68.0597997	–6.275	2.33E–15
AT4G21920	1.110184657	86.63013286	–6.286	1.93E–19
AT2G31345	0	14.75257431	–6.3171	0.00088894
AT5G19890	3.145523196	254.4229909	–6.3378	2.00E–56
AT1G80660	0	42.7023397	–6.4162	9.04E–10
AT5G61890	0	17.20348123	–6.5388	0.0002811
AT3G02480	0	35.58528308	–6.5874	3.45E–08
AT2G11810	0	39.16737781	–6.7257	6.43E–09
AT2G02250	0	14.89397279	–6.7459	0.00091905
AT4G34210	0	20.07858357	–6.7617	7.56E–05
AT3G55970	0	36.19800981	–6.8046	3.00E–08
AT4G33467	0	48.59394286	–6.8669	7.14E–11
AT2G15780	0	28.56249212	–6.9483	1.36E–06

AT1G21529	0	17.39201253	–6.9696	0.00030015
AT3G09922	0	58.77463312	–6.9894	5.63E–13
AT3G05950	0	23.51927981	–6.9899	1.62E–05
AT3G63380	10.03791961	1293.984585	–7.0102	4.85E–279
AT3G60120	0	12.01887045	–7.0214	0.0039499
AT2G47520	0	48.45254439	–7.0327	9.36E–11
AT2G04050	0	50.9034513	–7.1039	3.15E–11
AT3G43250	0	38.27185413	–7.1074	1.44E–08
AT3G19615	0	39.45017476	–7.1512	8.52E–09
AT4G25580	0	101.7597698	–7.2958	8.76E–22
AT1G69920	0	134.3756849	–7.3344	1.39E–28
AT3G22910	4.024419383	652.3654347	–7.3408	7.73E–138
AT1G34047	0	150.3065798	–7.344	6.56E–32
AT1G17710	0	22.57662331	–7.346	3.19E–05
AT5G39150	0	15.36530104	–7.3758	0.00095012
AT1G48710	0	15.88376212	–7.4236	0.00076404

AT3G27690	0	96.00956509	–7.4343	2.22E–20
AT1G71000	0	17.48627818	–7.5623	0.00039346
AT5G64750	2.590430867	529.537292	–7.6754	3.57E–109
AT4G32950	0	152.8046196	–7.7828	2.25E–31
AT1G06225	0	20.83270877	–7.8149	0.00010244
AT1G75600	0	84.6505542	–7.8376	1.85E–17
AT3G19920	0	34.6897594	–7.9656	2.30E–07
AT2G38240	2.729203949	682.4361772	–7.9661	3.43E–137
AT2G36255	0	103.2208874	–8.1238	1.14E–20
AT4G36600	0	13.76278498	–8.2169	0.0030729
AT1G52890	0	16.92068427	–8.5149	0.0009857
AT4G33720	0	159.167551	–8.5786	2.89E–30
AT5G12030	0	215.0199489	–8.5975	1.08E–40
AT2G42560	0	92.71026732	–8.6469	1.21E–17
AT4G33930	0	116.7480082	–8.7165	5.89E–22
AT1G11925	0	27.43130431	–9.2119	2.88E–05

AT2G02010	0	537.1256769	–9.2554	7.69E–94
AT4G25200	0	29.69367993	–9.3262	1.42E–05
AT5G55150	0	37.4705961	–9.6618	1.30E–06
AT5G24640	0	37.6591274	–9.6691	1.23E–06
AT3G46230	0	682.0591146	–10.147	1.96E–105
AT5G12020	0	109.253889	–10.206	5.54E–17
AT1G05675	0	79.65447472	–10.75	1.55E–11
AT1G53540	0	257.628023	–11.443	1.46E–32
