# Supplementary material for: Expression of Root Genes in Arabidopsis Seedlings Grown by Standard and Improved Growing Methods
Source: Int J Mol Sci. 2017 May 3;18(5):951. doi: 10.3390/ijms18050951 (PMC5454864; doi:10.3390/ijms18050951)
Supplement: Supplementary file 1 [file ijms-18-00951-s001.zip › Table S2.pdf]

Supplementary Table 2. List of all 2471 DEGs from TPG/SG comparison groups.

Gene_id	readcount_TPG	readcount_SG	log2 Fold change (TPG vs SG)	q value
AT5G07990	488.7099391	0	9.4377	5.89E–89
AT5G22890	22.94547757	0	8.9318	6.01E–05
AT1G51840	27.74801939	0	8.206	2.56E–06
AT1G73120	44.94686572	0	7.9019	3.51E–10
AT1G51830	115.1378615	0	7.674	6.68E–26
AT4G31940	94.77836799	0	7.5665	1.39E–21
AT5G17040	57.01479131	0	7.4376	2.61E–13
AT3G46900	20.44158825	0	7.1802	3.15E–05
AT5G23990	12.88887291	0	7.0998	0.0015143
AT5G17220	19.08702517	0	7.0813	5.93E–05
AT5G62210	222.2714867	1.785364618	6.96	1.49E–52
AT3G56080	16.17266219	0	6.8422	0.000241
AT5G04950	91.28934189	0	6.8366	6.10E–22
AT3G44450	10.38498359	0	6.7881	0.0049224
AT4G04750	50.0367391	0	6.2493	1.44E–12
AT2G23910	513.5025481	7.517324708	6.094	3.48E–126
AT5G59520	65.10112241	0	6.044	2.60E–16
AT2G25090	25.69565109	0	5.9252	9.00E–07
AT1G08430	13.87400969	0	5.8841	0.0006134
AT4G25100	308.4709552	5.497043693	5.8103	2.84E–76
AT5G35190	65.18321714	1.174581986	5.7943	2.13E–16
AT4G03540	15.63904643	0	5.7938	0.00023144

AT4G04840	99.58090981	1.832347898	5.7641	7.94E–25
AT2G25160	75.19877443	1.456481662	5.6901	7.55E–19
AT4G23496	16.70627794	0	5.6667	0.00012641
AT4G29740	16.62418321	0	5.6596	0.00013209
AT3G56290	61.8583805	1.268548545	5.6077	1.34E–15
AT3G28160	47.65599187	0	5.594	4.01E–12
AT3G09220	236.8022542	4.980227619	5.5713	1.19E–58
AT1G64500	30.53924027	0	5.5369	5.89E–08
AT4G37310	45.68571831	1.080615427	5.4018	1.26E–11
AT1G54970	22.65814601	0	5.3287	4.83E–06
AT5G47980	31.52437705	0	5.2202	3.68E–08
AT4G18630	15.47485697	0	5.1936	0.00026424
AT2G23630	13.7508676	0	5.1932	0.00067413
AT5G48010	334.4128904	9.302689327	5.1678	3.21E–82
AT1G66725	57.26107551	1.597431501	5.1637	2.24E–14
AT3G28345	89.72954198	2.584080369	5.1179	3.09E–22
AT4G11210	29.30781929	0	5.115	1.33E–07
AT3G22550	11.00069408	0	5.0639	0.0030628
AT5G08640	1498.639331	45.33886465	5.0468	0
AT5G48880	383.710777	12.12168609	4.9844	1.08E–93
AT3G49960	22.12453025	0	4.9724	7.40E–06
AT2G24762	14.61286228	0	4.9589	0.00045175
AT3G61220	92.27447867	3.006929883	4.9396	1.06E–22
AT4G12510	22.20662498	0	4.8846	7.44E–06
AT4G18422	17.85560419	0	4.8696	7.99E–05
AT3G62680	46.87609192	1.64441478	4.8332	1.06E–11
AT1G65060	243.739259	8.738889974	4.8017	4.89E–59
AT5G13930	5106.046041	184.9261878	4.7872	0

AT1G57590	16.78837268	0	4.7807	0.00015015
AT4G37160	19.0459778	0	4.7562	4.50E–05
AT4G12520	119.3246928	4.416428266	4.7559	5.76E–29
AT3G57020	422.295301	15.64543205	4.7544	7.04E–102
AT1G31320	11.37012037	0	4.749	0.0027626
AT5G04960	30.12876661	1.127598706	4.7398	1.14E–07
AT1G80340	24.71051431	0	4.7168	2.18E–06
AT4G08400	36.86053463	1.409498383	4.7088	3.02E–09
AT5G57785	48.64112866	1.879331177	4.6939	5.05E–12
AT5G10230	35.21863999	1.362515103	4.692	7.45E–09
AT3G14060	22.86338284	0	4.6787	6.07E–06
AT4G15480	182.2913522	7.141458473	4.6739	7.39E–44
AT1G49450	23.68433016	0	4.6556	3.99E–06
AT3G49330	14.20238862	0	4.6548	0.00064396
AT5G48000	203.471793	8.17509062	4.6375	8.47E–49
AT4G37760	73.3105956	2.959946604	4.6304	8.30E–18
AT1G78990	33.04312959	1.362515103	4.6	2.77E–08
AT2G46420	29.51305612	1.221565265	4.5946	1.85E–07
AT5G13900	39.24128185	1.64441478	4.5767	1.01E–09
AT5G07190	11.86268876	0	4.5684	0.0023095
AT4G08410	57.46631234	2.44313053	4.5559	5.59E–14
AT4G11780	10.91859934	0	4.5385	0.0038056
AT4G31330	11.8216414	0	4.5156	0.0024199
AT3G23290	11.329073	0	4.4542	0.0032094
AT5G58910	13.3814413	0	4.4534	0.0011023
AT2G22590	169.3614319	7.893190944	4.4234	5.74E–40
AT3G21560	370.5345725	17.28984683	4.4216	3.43E–87
AT3G02885	39.93908708	1.879331177	4.4095	9.42E–10

AT5G66690	216.4838081	10.19537164	4.4083	6.19E–51
AT5G47990	185.9856151	8.785873253	4.4039	8.66E–44
AT3G55120	677.6509646	32.46544608	4.3836	1.06E–158
AT5G22410	11.5753572	0	4.3597	0.00298
AT4G04745	28.77420354	1.409498383	4.3515	3.81E–07
AT1G42550	102.8236517	5.121177458	4.3276	3.54E–24
AT5G23660	31.11390339	1.550448221	4.3268	1.16E–07
AT5G09520	52.78691262	2.631063648	4.3265	1.26E–12
AT5G26270	13.87400969	0	4.2991	0.00094891
AT4G20240	40.71898703	2.114247574	4.2675	8.44E–10
AT1G52100	15.26962014	0	4.2568	0.00048222
AT5G19730	18.75864624	0	4.2489	7.97E–05
AT5G50760	31.11390339	1.64441478	4.2419	1.34E–07
AT5G66580	13.99715179	0	4.2188	0.00094766
AT2G34610	31.97589808	1.738381339	4.2012	9.24E–08
AT2G36090	24.13585118	1.315531824	4.1975	5.29E–06
AT3G26290	12.06792559	0	4.1975	0.0025813
AT2G34810	33.45360325	1.832347898	4.1904	4.36E–08
AT5G08050	48.47693919	2.678046927	4.178	1.84E–11
AT5G36180	62.30990152	3.476762678	4.1636	1.42E–14
AT1G64170	32.63265593	1.832347898	4.1546	7.19E–08
AT4G11050	14.12029389	0	4.1439	0.0009471
AT3G49120	410.2273754	23.91448923	4.1005	3.01E–93
AT4G28740	20.93415664	1.221565265	4.0991	3.13E–05
AT3G08040	39.44651868	2.302180692	4.0988	2.44E–09
AT3G44990	185.9035204	10.90012083	4.0921	2.61E–42
AT5G49080	41.53993435	2.44313053	4.0877	8.57E–10
AT1G77760	215.7039081	12.779452	4.0772	5.71E–49

AT1G16400	69.24690637	4.13452859	4.066	5.84E–16
AT4G02850	11.73954666	0	4.0581	0.0033456
AT5G50100	46.67085509	2.818996766	4.0493	6.85E–11
AT4G04850	43.51020791	2.631063648	4.0476	3.51E–10
AT1G22750	65.79892763	3.993578751	4.0423	3.86E–15
AT2G40130	21.63196186	1.315531824	4.0394	2.39E–05
AT5G26310	26.72183524	1.64441478	4.0224	1.89E–06
AT4G17280	75.65029546	4.839277781	3.9665	3.60E–17
AT2G25000	16.74732531	1.080615427	3.954	0.00031092
AT1G55990	82.46415821	5.356093855	3.9445	1.30E–18
AT5G05270	746.2411131	48.8626106	3.9328	1.10E–165
AT3G45710	80.82226357	5.309110575	3.9282	3.29E–18
AT1G15550	18.59445678	1.221565265	3.9281	0.00012746
AT1G23010	76.47124278	5.027210899	3.9271	2.96E–17
AT3G58990	60.01124903	3.946595472	3.9266	1.24E–13
AT1G12740	87.43088949	5.872909928	3.896	1.44E–19
AT1G14160	22.69919337	1.550448221	3.8719	1.82E–05
AT5G13630	88.7444052	6.107826326	3.8609	9.38E–20
AT2G24980	115.5072878	8.034140782	3.8457	1.55E–25
AT5G19600	28.85629827	2.020281015	3.8363	9.41E–07
AT4G13390	55.00347038	3.852628913	3.8356	2.25E–12
AT2G15370	16.04952009	1.127598706	3.8312	0.00050507
AT3G52740	56.48117556	3.993578751	3.822	1.15E–12
AT1G78370	47.73808661	3.382796119	3.8189	8.77E–11
AT1G01580	145.3076755	10.33632147	3.8133	6.98E–32
AT2G18050	53.81309677	3.852628913	3.804	4.63E–12
AT5G47950	19.57959356	1.409498383	3.7961	9.46E–05
AT5G64940	62.63828045	4.557378104	3.7808	6.59E–14

AT2G03090	82.05368455	6.013859767	3.7702	4.75E–18
AT4G35420	15.35171487	1.127598706	3.7671	0.00076098
AT1G51860	46.95818665	3.476762678	3.7556	1.64E–10
AT5G48850	25.77774582	1.926314457	3.7422	5.15E–06
AT2G21100	36.4090136	2.725030207	3.74	2.99E–08
AT4G08040	17.52722526	1.315531824	3.7359	0.0002818
AT1G22430	22.86338284	1.738381339	3.7172	2.22E–05
AT3G01420	353.0073472	26.96840239	3.7104	8.71E–76
AT1G13830	19.66168829	1.503464942	3.709	0.00010443
AT4G15500	22.65814601	1.738381339	3.7042	2.51E–05
AT4G15393	83.03882133	6.389726002	3.7	4.75E–18
AT1G07500	12.19106769	0	3.6975	0.0036749
AT4G22460	16.91151477	1.315531824	3.6843	0.00040467
AT4G12330	15.63904643	1.221565265	3.6783	0.00074226
AT1G18870	31.81170862	2.49011381	3.6753	3.32E–07
AT3G44310	351.2012631	27.6261683	3.6682	7.51E–75
AT4G01680	11.94478349	0	3.6681	0.0042547
AT5G41040	166.570211	13.10833496	3.6676	1.27E–35
AT4G30470	189.7209254	14.94068286	3.6666	1.61E–40
AT1G73620	27.33754573	2.161230854	3.661	2.94E–06
AT1G10960	78.11313741	6.201792884	3.6548	7.04E–17
AT4G33610	115.7125246	9.208722768	3.6514	8.87E–25
AT2G39310	222.1483446	17.71269634	3.6487	2.90E–47
AT4G36610	57.71259653	4.604361384	3.6478	1.43E–12
AT2G43100	64.11598562	5.121177458	3.6461	6.55E–14
AT3G25790	15.22857277	1.221565265	3.64	0.00094519
AT1G73650	109.9658934	8.832856532	3.638	1.66E–23
AT5G20700	290.6974457	23.49163971	3.6293	1.50E–61

AT4G28250	119.0373613	9.631572283	3.6275	2.25E–25
AT5G62920	23.76642489	1.926314457	3.625	1.72E–05
AT1G01420	12.10897296	0	3.6174	0.004148
AT5G24770	86.93832109	7.141458473	3.6057	1.45E–18
AT5G17050	281.2155042	23.25672332	3.596	3.47E–59
AT4G25250	13.6277255	1.127598706	3.5952	0.0021191
AT4G26850	119.1605034	9.86648868	3.5942	3.04E–25
AT5G37690	76.34810068	6.342742723	3.5894	2.58E–16
AT1G35515	12.35525715	1.033632147	3.5793	0.0038489
AT2G19970	29.14362983	2.44313053	3.5764	1.54E–06
AT5G16530	12.31420979	1.033632147	3.5745	0.0039408
AT4G35060	66.98930124	5.637993531	3.5707	2.56E–14
AT2G24190	17.1167516	1.456481662	3.5548	0.00044545
AT5G47110	58.45144912	4.980227619	3.553	1.64E–12
AT4G31910	70.27309052	6.013859767	3.5466	6.24E–15
AT5G06630	69.0827169	5.919893208	3.5447	1.11E–14
AT1G14960	43.55125528	3.758662354	3.5344	2.02E–09
AT5G64120	755.3536283	65.40072496	3.5298	1.17E–156
AT3G09600	15.72114116	1.362515103	3.5284	0.00087529
AT3G46270	13.54563077	1.174581986	3.5276	0.0023868
AT3G20370	302.7243239	26.31063648	3.5243	8.11E–63
AT5G49270	27.50173519	2.396147251	3.5207	3.81E–06
AT5G62720	40.92422386	3.570729237	3.5187	7.24E–09
AT4G29905	163.1222323	14.28291695	3.5136	6.55E–34
AT4G00880	41.17050805	3.617712516	3.5085	6.73E–09
AT5G06640	132.0083289	11.60487002	3.5078	1.83E–27
AT2G22980	13.21725184	1.174581986	3.4922	0.0028772
AT1G34040	42.77135533	3.805645634	3.4904	3.46E–09

AT1G67195	36.36796624	3.241846281	3.4878	6.87E–08
AT2G25530	72.73593247	6.483692561	3.4878	2.90E–15
AT4G15290	16.29580428	1.456481662	3.4839	0.00071936
AT2G40230	12.56049398	1.127598706	3.4776	0.0039278
AT2G48130	144.7330124	13.0143684	3.4752	6.83E–30
AT1G54540	29.14362983	2.631063648	3.4695	2.05E–06
AT3G10710	14.48972018	1.315531824	3.4613	0.001693
AT4G19690	218.3309395	19.92091048	3.4542	9.81E–45
AT5G26010	24.05375645	2.208214133	3.4453	2.24E–05
AT4G25640	799.1101204	73.57581558	3.4411	9.39E–163
AT5G48485	17.85560419	1.64441478	3.4407	0.00038213
AT1G78090	55.5781335	5.121177458	3.44	1.15E–11
AT2G29340	13.7508676	1.268548545	3.4383	0.0024303
AT2G28470	76.30705331	7.047491914	3.4366	7.84E–16
AT1G77990	14.20238862	1.315531824	3.4324	0.0020067
AT2G15620	381.4121245	35.33142613	3.4323	1.25E–77
AT1G30530	70.92984837	6.57765912	3.4307	9.98E–15
AT4G34881	351.2012631	32.6533792	3.427	2.02E–71
AT3G48100	46.0551446	4.322461707	3.4134	1.06E–09
AT5G07475	44.82372362	4.228495148	3.406	1.93E–09
AT3G51820	33.98721901	3.241846281	3.3901	2.85E–07
AT4G13770	135.0047866	13.0143684	3.3748	2.60E–27
AT5G64700	12.56049398	1.221565265	3.3621	0.004523
AT5G24120	55.00347038	5.356093855	3.3603	2.32E–11
AT1G65970	60.21648586	5.919893208	3.3465	2.37E–12
AT2G41370	25.69565109	2.537097089	3.3403	1.38E–05
AT2G05620	20.40054088	2.020281015	3.336	0.00014778
AT2G01530	456.4467094	45.29188137	3.3331	6.99E–91

AT1G69310	102.4952728	10.33632147	3.3098	1.58E–20
AT3G09270	244.7243958	24.76018826	3.3051	1.69E–48
AT4G19030	59.19030171	6.013859767	3.299	5.04E–12
AT2G37970	313.1503549	31.85466345	3.2973	6.89E–62
AT3G46130	16.09056745	1.64441478	3.2906	0.0010665
AT2G37460	23.39699859	2.396147251	3.2875	4.37E–05
AT2G47360	21.91929342	2.255197413	3.2809	8.47E–05
AT2G43920	310.6464656	31.99561329	3.2793	3.89E–61
AT2G47010	17.77350946	1.832347898	3.278	0.00052803
AT4G22666	41.74517118	4.322461707	3.2717	1.38E–08
AT3G09260	9154.876124	952.1631409	3.2653	0
AT3G01260	18.06084102	1.879331177	3.2646	0.00047829
AT4G35030	30.70342973	3.194863001	3.2646	1.88E–06
AT5G56080	50.48826013	5.262127296	3.2622	3.05E–10
AT3G09925	20.68787244	2.161230854	3.2589	0.00015321
AT5G48800	19.33330937	2.020281015	3.2585	0.00027895
AT1G13510	13.46353603	1.409498383	3.2558	0.0035043
AT4G09990	23.19176177	2.44313053	3.2468	5.25E–05
AT1G64780	144.4867282	15.31654909	3.2378	2.63E–28
AT5G48290	34.02826638	3.617712516	3.2336	4.82E–07
AT1G28130	54.59299672	5.825926649	3.2282	5.96E–11
AT1G68238	18.47131468	1.973297736	3.2266	0.00043063
AT5G13580	68.95957481	7.37637487	3.2248	1.09E–13
AT4G37470	63.99284353	6.859558796	3.2217	1.00E–12
AT3G54600	55.98860716	6.013859767	3.2188	3.42E–11
AT4G37750	33.57674535	3.617712516	3.2143	6.33E–07
AT2G24820	20.40054088	2.208214133	3.2077	0.00019459
AT3G54590	210.4498453	22.97482364	3.1954	1.19E–40

AT5G24380	66.37359075	7.282408311	3.1881	4.46E–13
AT5G66460	16.45999375	1.832347898	3.1672	0.0011241
AT1G13250	14.28448335	1.597431501	3.1606	0.0028532
AT2G28690	13.01201501	1.456481662	3.1593	0.0048712
AT5G07130	29.34886666	3.28882956	3.1577	4.74E–06
AT2G18800	20.52368298	2.302180692	3.1562	0.00020727
AT4G28660	23.35595123	2.631063648	3.1501	6.26E–05
AT4G36530	22.00138815	2.49011381	3.1433	0.00011337
AT1G07610	346.0703424	39.2780216	3.1393	9.30E–66
AT3G50300	37.96881351	4.322461707	3.1349	1.31E–07
AT1G69230	25.49041426	2.912963325	3.1294	2.68E–05
AT2G48080	19.25121463	2.208214133	3.124	0.00038153
AT1G01600	17.15779897	1.973297736	3.1202	0.00091731
AT1G50250	126.5900766	14.70576646	3.1057	4.99E–24
AT2G38380	539.0750571	62.67569476	3.1045	2.04E–101
AT2G34080	29.43096139	3.429779398	3.1011	5.51E–06
AT3G25930	76.59438487	8.926823091	3.101	1.07E–14
AT4G12030	32.63265593	3.805645634	3.1001	1.44E–06
AT4G11190	50.24197593	5.919893208	3.0853	9.06E–10
AT3G21520	17.1167516	2.020281015	3.0828	0.0010004
AT5G61420	34.02826638	4.040562031	3.0741	8.84E–07
AT4G02290	175.1080632	20.86057607	3.0694	8.54E–33
AT5G06330	15.35171487	1.832347898	3.0666	0.0021456
AT4G14440	54.92137565	6.57765912	3.0617	1.44E–10
AT5G23190	64.32122245	7.752241106	3.0526	2.93E–12
AT3G63520	212.0506925	25.60588729	3.0499	2.00E–39
AT3G59340	15.88533062	1.926314457	3.0438	0.0017979
AT5G14200	196.4526935	23.86750595	3.0411	1.84E–36

AT1G03820	30.53924027	3.711679075	3.0405	4.28E–06
AT4G00700	42.48402376	5.168160737	3.0392	3.01E–08
AT2G34490	18.14293575	2.208214133	3.0385	0.00071936
AT3G45700	13.87400969	1.691398059	3.0361	0.0040957
AT3G22620	221.1632078	27.01538567	3.0333	6.51E–41
AT1G58360	37.55833985	4.604361384	3.0281	2.47E–07
AT3G62270	102.9057465	12.68548545	3.0201	3.73E–19
AT2G21960	29.67724559	3.664695795	3.0176	6.62E–06
AT2G48140	87.47193685	10.85313755	3.0107	2.57E–16
AT1G60270	33.04312959	4.13452859	2.9986	1.80E–06
AT1G13970	19.4154041	2.44313053	2.9904	0.0004788
AT2G30210	129.217108	16.30319796	2.9866	9.98E–24
AT5G67400	36.61425043	4.651344663	2.9767	4.53E–07
AT1G10470	131.3515711	16.77303076	2.9692	5.41E–24
AT5G42590	21.63196186	2.772013486	2.9642	0.00020829
AT3G60550	47.40970768	6.154809605	2.9454	6.50E–09
AT3G20100	59.64182273	7.752241106	2.9436	4.58E–11
AT1G62870	20.56473034	2.678046927	2.9409	0.00033962
AT5G06270	16.21370955	2.114247574	2.939	0.0019194
AT4G29140	20.15425668	2.631063648	2.9374	0.00040274
AT1G21910	115.6714773	15.17559926	2.9302	6.27E–21
AT4G09760	20.40054088	2.678046927	2.9294	0.00037318
AT1G53090	35.71120838	4.698327943	2.9262	8.16E–07
AT1G20160	26.7628826	3.523745957	2.9251	2.96E–05
AT5G24090	19.62064093	2.584080369	2.9246	0.0005132
AT4G25820	43.71544474	5.77894337	2.9193	3.37E–08
AT2G20750	15.26962014	2.020281015	2.918	0.0028772
AT1G46480	20.56473034	2.725030207	2.9158	0.00036156

AT1G52870	17.3630358	2.302180692	2.9149	0.0012699
AT1G06000	164.3536533	21.80024165	2.9144	1.87E–29
AT3G29250	104.2603095	13.86006743	2.9112	8.43E–19
AT1G44970	18.01979365	2.396147251	2.9108	0.00098975
AT2G41170	21.18044083	2.818996766	2.9095	0.00028867
AT4G14690	65.26531187	8.691906694	2.9086	6.14E–12
AT3G26440	104.2603095	13.90705071	2.9063	8.95E–19
AT3G26210	24.8336564	3.335812839	2.8962	6.96E–05
AT1G55320	142.5985493	19.21616129	2.8916	2.01E–25
AT1G78570	1139.474879	153.8232568	2.889	3.16E–201
AT1G30510	320.1284071	43.22461707	2.8887	1.21E–56
AT1G48930	20.15425668	2.725030207	2.8867	0.00045611
AT2G47540	29.96457715	4.08754531	2.874	9.89E–06
AT5G54230	25.81879319	3.523745957	2.8732	5.08E–05
AT4G00370	75.36296389	10.28933819	2.8727	1.46E–13
AT1G52070	72.24336408	9.913471959	2.8654	5.45E–13
AT2G22920	41.99145537	5.825926649	2.8495	9.77E–08
AT1G74460	129.0118712	17.99459602	2.8419	1.11E–22
AT1G24280	106.2716305	14.8467163	2.8395	9.72E–19
AT1G49430	53.6489073	7.517324708	2.8353	1.09E–09
AT2G25810	54.96242301	7.705257826	2.8345	6.54E–10
AT5G03760	82.54625294	11.74581986	2.8131	1.53E–14
AT4G37070	67.56396436	9.725538842	2.7964	6.33E–12
AT4G09750	85.46061592	12.40358577	2.7845	6.71E–15
AT1G67750	16.78837268	2.44313053	2.7807	0.0021189
AT5G02780	45.97304987	6.718608958	2.7746	3.16E–08
AT1G02730	43.63335001	6.389726002	2.7716	7.90E–08
AT5G42500	36.7784399	5.403077134	2.767	1.11E–06

AT1G13420	64.56750665	9.490622444	2.7662	2.63E–11
AT2G22510	136.1541129	20.15582687	2.756	2.99E–23
AT5G53370	93.71113648	13.90705071	2.7524	3.99E–16
AT4G33110	20.11320932	3.006929883	2.7418	0.00066776
AT4G24130	120.3919243	18.0415793	2.7383	1.75E–20
AT2G32990	63.2950383	9.490622444	2.7375	5.44E–11
AT1G78120	58.73878068	8.832856532	2.7334	3.21E–10
AT5G25460	154.8306644	23.3037066	2.7321	3.57E–26
AT5G38030	88.12869471	13.34325136	2.7235	4.84E–15
AT1G12110	208.3153822	31.57276378	2.722	5.41E–35
AT2G28950	18.26607785	2.772013486	2.7202	0.001391
AT1G07090	21.2214882	3.241846281	2.7106	0.00048147
AT4G21230	15.35171487	2.349163971	2.7082	0.0041563
AT3G02020	48.47693919	7.42335815	2.7072	1.85E–08
AT5G65380	289.5070721	44.5401489	2.7004	3.75E–48
AT5G01840	18.26607785	2.818996766	2.6959	0.0014758
AT5G07680	35.91644521	5.544026972	2.6956	2.17E–06
AT5G58330	30.33400344	4.745311222	2.6764	1.85E–05
AT3G23530	201.0910458	31.5257805	2.6732	3.26E–33
AT1G80240	55.08556511	8.644923415	2.6717	2.02E–09
AT4G40090	35.25968736	5.544026972	2.669	3.13E–06
AT3G27170	95.59931531	15.03464942	2.6687	5.62E–16
AT5G19940	32.7968454	5.168160737	2.6658	7.80E–06
AT2G33250	35.38282945	5.591010252	2.6619	3.10E–06
AT5G24850	64.93693294	10.28933819	2.6579	5.81E–11
AT2G31570	194.7697515	30.86801458	2.6576	5.37E–32
AT5G51110	21.91929342	3.476762678	2.6564	0.00043567
AT3G50400	28.11744568	4.463411546	2.6552	4.54E–05

AT1G07590	517.8535689	82.220739	2.655	1.40E–84
AT4G18640	47.45075504	7.564307988	2.6492	3.93E–08
AT4G00080	24.99784587	3.993578751	2.646	0.00014647
AT5G38020	121.6643927	19.45107768	2.645	5.00E–20
AT3G32980	1072.157199	171.4889699	2.6443	3.29E–174
AT5G09530	457.8012725	73.34089919	2.642	1.97E–74
AT5G24930	60.13439113	9.678555562	2.6353	4.18E–10
AT1G16060	29.10258246	4.698327943	2.6309	3.48E–05
AT4G30980	20.93415664	3.382796119	2.6296	0.00066898
AT4G32770	17.73246209	2.865980045	2.6293	0.0021065
AT4G20390	78.76989527	12.73246872	2.6291	4.70E–13
AT5G28500	20.89310927	3.382796119	2.6267	0.00068388
AT2G17500	244.1086853	39.60690456	2.6237	1.60E–39
AT2G40480	56.68641238	9.208722768	2.6219	1.64E–09
AT2G01900	17.89665156	2.912963325	2.6191	0.002036
AT3G57010	100.2376677	16.35018124	2.616	2.06E–16
AT4G01440	35.13654526	5.73196009	2.6159	4.20E–06
AT1G13080	21.54986713	3.523745957	2.6125	0.00056459
AT1G02810	115.5483352	18.93426161	2.6094	8.53E–19
AT1G08990	25.61355636	4.228495148	2.5987	0.00013761
AT4G18570	18.18398312	3.006929883	2.5963	0.0019442
AT4G21740	19.00493044	3.147879722	2.5939	0.0014562
AT2G28250	60.62695952	10.0544218	2.5921	5.00E–10
AT3G56940	30.58028764	5.074194178	2.5914	2.40E–05
AT1G74470	78.64675317	13.06135168	2.5901	7.53E–13
AT1G12370	42.03250274	7.000508635	2.586	4.13E–07
AT3G44550	81.60216352	13.62515103	2.5823	2.80E–13
AT4G08150	20.76996717	3.476762678	2.5787	0.00081583

AT3G16450	676.9531593	113.4646198	2.5768	2.66E–107
AT1G75500	237.8694857	39.88880423	2.5761	7.71E–38
AT1G78510	43.46916055	7.329391591	2.5682	2.76E–07
AT5G53110	20.81101454	3.523745957	2.5622	0.00084131
AT1G62980	18.01979365	3.053913163	2.5609	0.0022419
AT1G79410	20.31844615	3.476762678	2.547	0.0010398
AT5G22020	237.0895858	40.78148654	2.5394	3.52E–37
AT3G50640	28.32268251	4.88626106	2.5352	6.69E–05
AT1G72230	34.80816633	6.013859767	2.5331	7.01E–06
AT4G25470	30.70342973	5.309110575	2.5319	2.96E–05
AT2G45750	63.25399094	10.99408739	2.5244	3.51E–10
AT4G11650	193.0047147	33.54606151	2.5244	3.79E–30
AT2G41660	124.0040926	21.56532526	2.5236	1.72E–19
AT1G49310	32.63265593	5.684976811	2.5211	1.59E–05
AT2G32430	28.40477724	4.980227619	2.5119	7.12E–05
AT5G57625	25.40831953	4.463411546	2.5091	0.00020399
AT1G59940	17.1167516	3.006929883	2.509	0.0034331
AT5G10130	33.86407691	5.966876487	2.5047	1.12E–05
AT1G06650	24.25899328	4.275478428	2.5044	0.00030886
AT2G36570	77.78475849	13.71911759	2.5033	2.64E–12
AT1G72240	23.60223542	4.181511869	2.4968	0.00039525
AT4G38620	81.80740035	14.56481662	2.4897	7.63E–13
AT2G25060	18.71759888	3.335812839	2.4883	0.0021204
AT1G74090	56.56327029	10.10140508	2.4853	5.02E–09
AT5G14650	20.48263561	3.664695795	2.4826	0.0011787
AT5G11420	25.36727216	4.557378104	2.4767	0.00023296
AT1G72160	229.2084915	41.25131934	2.4741	5.09E–35
AT3G26300	22.65814601	4.08754531	2.4707	0.00059227

AT2G41380	62.35094889	11.27598706	2.4672	8.02E–10
AT4G32650	93.46485228	16.91398059	2.4662	1.79E–14
AT4G03210	97.85692044	17.71269634	2.4659	3.95E–15
AT1G18060	18.92283571	3.429779398	2.4639	0.0021123
AT3G45070	44.74162889	8.128107341	2.4606	3.45E–07
AT3G18170	35.13654526	6.389726002	2.4591	9.02E–06
AT5G10470	108.1598093	19.68599408	2.4579	1.27E–16
AT3G14940	376.0349195	68.45463813	2.4576	7.01E–57
AT3G25640	18.75864624	3.429779398	2.4514	0.0023052
AT3G15680	20.81101454	3.805645634	2.4511	0.0011574
AT3G43670	49.25683915	9.02078965	2.449	8.06E–08
AT1G54010	32.5505612	5.966876487	2.4476	2.29E–05
AT3G18710	22.78128811	4.181511869	2.4458	0.00061557
AT3G50740	94.36789433	17.33683011	2.4445	1.77E–14
AT4G31890	24.25899328	4.463411546	2.4423	0.00038101
AT1G66800	22.45290918	4.13452859	2.4411	0.00069402
AT3G25110	57.54840707	10.61822115	2.4382	5.25E–09
AT3G17040	26.39345631	4.88626106	2.4334	0.00019258
AT4G18760	18.75864624	3.476762678	2.4317	0.0024234
AT4G02410	22.04243552	4.08754531	2.431	0.00081942
AT3G53980	61.24267001	11.36995362	2.4293	1.64E–09
AT3G58810	26.27031421	4.88626106	2.4266	0.00020597
AT2G05440	4932.785109	917.8183636	2.4261	0
AT3G59370	50.32407066	9.396655886	2.421	6.88E–08
AT2G47460	151.6289698	28.33091749	2.4201	9.29E–23
AT3G23210	18.59445678	3.476762678	2.4191	0.0026433
AT2G22240	41.37574488	7.752241106	2.4161	1.42E–06
AT4G25090	24.66946694	4.651344663	2.407	0.00037606

AT5G53250	80.61702674	15.22258253	2.4049	3.09E–12
AT2G41310	64.48541192	12.21565265	2.4002	7.19E–10
AT5G26670	17.56827263	3.335812839	2.3969	0.0038804
AT4G34830	17.73246209	3.382796119	2.3901	0.0037444
AT5G42250	84.39338441	16.11526484	2.3887	1.07E–12
AT2G20724	17.19884634	3.28882956	2.3867	0.0044976
AT1G73885	45.84990777	8.785873253	2.3837	3.93E–07
AT3G25190	118.9142192	22.78689052	2.3836	1.14E–17
AT5G45380	40.67793966	7.799224385	2.3828	2.17E–06
AT1G30650	34.76711896	6.671625679	2.3816	1.51E–05
AT3G44540	40.34956073	7.752241106	2.3799	2.47E–06
AT1G12380	23.64328279	4.557378104	2.3752	0.00058714
AT5G44110	141.5723652	27.29728535	2.3747	7.33E–21
AT4G02270	31.77066125	6.154809605	2.3679	4.30E–05
AT1G47530	388.0207504	75.17324708	2.3678	1.89E–56
AT5G26290	72.69488511	14.14196711	2.3619	6.92E–11
AT4G33950	17.85560419	3.476762678	2.3606	0.0038803
AT5G10180	25.81879319	5.027210899	2.3606	0.00030723
AT1G48500	29.0615351	5.684976811	2.3539	0.00010984
AT3G63540	25.90088792	5.074194178	2.3518	0.00030913
AT3G23810	877.797921	172.2876857	2.3491	2.03E–126
AT1G35250	28.938393	5.684976811	2.3478	0.00011727
AT4G26320	53.7720494	10.57123787	2.3467	3.86E–08
AT1G16720	64.60855401	12.73246872	2.3432	1.19E–09
AT1G65310	62.76142255	12.40358577	2.3391	2.25E–09
AT2G30840	22.98652494	4.557378104	2.3345	0.00082426
AT3G22800	85.05014226	16.86699731	2.3341	1.71E–12
AT5G43630	129.0118712	25.74683713	2.325	1.23E–18

AT3G22840	249.7321745	49.84925947	2.3247	1.01E–35
AT2G31790	39.44651868	7.893190944	2.3212	4.61E–06
AT3G54770	38.7076661	7.752241106	2.3199	5.88E–06
AT4G18020	24.50527748	4.93324434	2.3125	0.00055492
AT4G14550	33.82302955	6.812575517	2.3117	2.92E–05
AT3G19450	534.2725153	107.9205928	2.3076	1.48E–75
AT3G15354	35.34178209	7.141458473	2.3071	1.84E–05
AT3G52370	54.26461779	11.04107067	2.2971	4.94E–08
AT3G50060	96.2150258	19.59202752	2.296	7.84E–14
AT1G30870	42.11459747	8.597940135	2.2923	2.38E–06
AT4G30670	45.31629202	9.255706047	2.2916	8.68E–07
AT1G70370	110.0069408	22.50499085	2.2893	1.05E–15
AT5G44550	129.5044396	26.54555288	2.2865	2.23E–18
AT1G64110	34.27455057	7.047491914	2.282	2.94E–05
AT4G12880	141.0797968	29.08264997	2.2783	6.64E–20
AT3G24300	86.5688948	17.85364618	2.2776	2.17E–12
AT4G23490	23.39699859	4.839277781	2.2735	0.00089138
AT5G61480	41.78621854	8.644923415	2.2731	2.98E–06
AT5G58770	18.59445678	3.852628913	2.271	0.0039014
AT3G45780	38.29719244	7.940174223	2.27	8.91E–06
AT4G27657	51.18606535	10.61822115	2.2692	1.62E–07
AT2G22990	36.45006097	7.564307988	2.2686	1.60E–05
AT5G57150	47.9843708	9.960455239	2.2683	4.42E–07
AT5G62710	23.23280913	4.839277781	2.2633	0.00097103
AT2G47200	19.99006722	4.181511869	2.2572	0.0026704
AT5G55730	34.80816633	7.282408311	2.2569	2.84E–05
AT1G64390	121.6643927	25.51192073	2.2537	4.89E–17
AT5G37550	45.23419728	9.490622444	2.2528	1.16E–06

AT1G65560	44.04382367	9.255706047	2.2505	1.71E–06
AT3G51860	69.78052212	14.70576646	2.2464	6.12E–10
AT1G71870	25.40831953	5.356093855	2.246	0.0005354
AT2G46430	23.60223542	4.980227619	2.2446	0.00092627
AT1G72430	36.2858715	7.658274547	2.2443	1.92E–05
AT1G32450	102.9057465	21.75325838	2.242	2.14E–14
AT5G41140	61.98152259	13.10833496	2.2414	7.09E–09
AT5G23020	227.8539284	48.25182797	2.2395	2.44E–31
AT4G19810	52.49958106	11.13503722	2.2372	1.39E–07
AT5G40890	192.0606253	40.78148654	2.2356	2.09E–26
AT5G47060	30.29295608	6.436709282	2.2346	0.00012629
AT3G50820	28.48687197	6.060843046	2.2327	0.00022232
AT5G05500	21.63196186	4.604361384	2.2321	0.001762
AT1G64590	48.76427076	10.38330475	2.2316	4.53E–07
AT1G04280	86.36365797	18.41744554	2.2294	4.35E–12
AT5G22940	25.03889323	5.356093855	2.2249	0.00064885
AT5G67070	25.40831953	5.450060414	2.221	0.00058983
AT3G23510	51.18606535	11.04107067	2.2129	2.51E–07
AT2G16430	55.08556511	11.8867697	2.2123	7.70E–08
AT1G13930	937.1934595	202.8268173	2.2081	3.43E–126
AT1G50630	22.82233547	4.980227619	2.1962	0.0013833
AT3G54390	21.50881976	4.698327943	2.1947	0.0020626
AT4G32460	32.6737033	7.141458473	2.1938	7.44E–05
AT2G35770	31.81170862	6.953525355	2.1937	9.63E–05
AT2G44230	21.05729874	4.604361384	2.1932	0.0023665
AT5G40730	57.54840707	12.59151889	2.1923	4.33E–08
AT1G31050	32.63265593	7.141458473	2.192	7.59E–05
AT1G18250	19.94901985	4.369444987	2.1908	0.0032791

AT1G65985	38.78976083	8.503973576	2.1895	1.23E−05
AT2G19130	39.85699234	8.738889974	2.1893	8.91E−06
AT4G00430	132.3777552	29.08264997	2.1864	6.72E−18
AT1G79040	155.4463749	34.15684414	2.1862	6.27E−21
AT3G07340	33.12522433	7.282408311	2.1854	6.79E−05
AT2G23610	59.19030171	13.0143684	2.1853	2.82E−08
AT2G16750	66.66092231	14.65878318	2.1851	2.99E−09
AT5G26280	800.5057309	176.1872979	2.1838	1.71E−106
AT4G36180	51.80177584	11.4169369	2.1818	2.66E−07
AT1G01750	35.95749258	7.940174223	2.179	3.03E−05
AT5G38010	24.01270908	5.309110575	2.1773	0.0010397
AT5G15910	59.6828701	13.20230152	2.1765	2.64E−08
AT3G21510	262.9083789	58.16529993	2.1763	5.38E−35
AT4G30450	38.87185556	8.644923415	2.1688	1.35E−05
AT2G40460	50.48826013	11.22900378	2.1687	4.34E−07
AT5G61640	38.2150977	8.503973576	2.1679	1.65E−05
AT5G17010	156.9651274	35.14349301	2.1591	7.58E−21
AT1G71960	24.9567985	5.591010252	2.1583	0.00084836
AT4G16190	497.904549	111.6792552	2.1565	2.27E−65
AT5G17160	31.31914022	7.047491914	2.1519	0.00013657
AT5G66230	21.05729874	4.745311222	2.1497	0.0027015
AT3G04720	120.2277349	27.15633551	2.1464	5.62E−16
AT3G21260	37.64043458	8.503973576	2.1461	2.23E−05
AT1G28400	304.6125028	68.87748764	2.1449	7.74E−40
AT5G23750	27.83011412	6.295759443	2.1442	0.00039319
AT4G17250	35.38282945	8.034140782	2.1388	4.47E−05
AT2G39220	26.06507738	5.919893208	2.1385	0.00066777
AT1G19715	29.75934032	6.765592238	2.1371	0.00023255

AT2G13820	66.33254338	15.0816327	2.1369	5.38E–09
AT3G52900	20.31844615	4.651344663	2.1271	0.0035661
AT4G30190	1271.483208	291.2023659	2.1264	4.60E–164
AT1G64640	49.62626544	11.36995362	2.1259	7.79E–07
AT1G04220	75.56820072	17.33683011	2.1239	4.21E–10
AT3G29410	29.22572456	6.718608958	2.121	0.00029134
AT1G11545	50.20092856	11.55788674	2.1188	6.97E–07
AT3G11720	27.74801939	6.389726002	2.1186	0.00044831
AT5G21950	20.35949351	4.698327943	2.1155	0.003654
AT5G41670	382.930877	88.51649844	2.1131	3.55E–49
AT5G54510	29.02048773	6.718608958	2.1108	0.0003234
AT1G31070	74.66515867	17.28984683	2.1105	6.39E–10
AT3G03130	21.30358293	4.93324434	2.1105	0.0028533
AT5G49300	21.0983461	4.88626106	2.1103	0.003024
AT5G23730	31.23704549	7.235425032	2.1101	0.00017152
AT4G24780	113.1265406	26.21666992	2.1094	8.98E–15
AT5G12940	36.69634516	8.503973576	2.1094	3.60E–05
AT4G34160	32.59160857	7.564307988	2.1072	0.00011775
AT5G23010	46.75294982	10.85313755	2.1069	2.06E–06
AT3G22600	311.3442708	72.44821688	2.1035	7.88E–40
AT3G54580	605.3665531	141.3257045	2.0988	4.80E–77
AT4G23500	28.97944036	6.765592238	2.0987	0.00034478
AT5G42860	40.80108176	9.537605724	2.0969	1.20E–05
AT2G16890	94.20370487	22.03515805	2.096	2.71E–12
AT1G67360	126.9595029	29.928349	2.0848	2.60E–16
AT3G20110	26.10612475	6.154809605	2.0846	0.00081583
AT1G13300	62.35094889	14.70576646	2.084	2.90E–08
AT5G60890	60.42172269	14.28291695	2.0808	5.19E–08

AT5G16770	20.35949351	4.839277781	2.0728	0.0041711
AT2G38170	36.49110833	8.691906694	2.0698	4.77E–05
AT3G28050	46.46561826	11.08805394	2.0672	2.99E–06
AT4G37800	68.79538534	16.4441478	2.0647	5.66E–09
AT4G16260	125.1534188	29.928349	2.0641	6.54E–16
AT2G18150	106.7231515	25.55890401	2.062	1.30E–13
AT1G19050	26.27031421	6.295759443	2.061	0.00085577
AT1G45130	114.8094826	27.53220174	2.06	1.34E–14
AT5G05180	23.47909333	5.637993531	2.0581	0.0018747
AT5G09760	28.36372988	6.812575517	2.0578	0.00048964
AT3G62040	61.16057527	14.70576646	2.0562	5.33E–08
AT5G11110	59.51868064	14.32990023	2.0543	8.57E–08
AT5G16590	74.13154292	17.90062946	2.0501	1.51E–09
AT5G58860	131.1463342	31.76069689	2.0459	1.76E–16
AT4G24140	44.16696577	10.71218771	2.0437	6.63E–06
AT4G12480	46.01409724	11.1820205	2.0409	4.07E–06
AT5G54040	22.9044302	5.591010252	2.0344	0.0023849
AT4G20820	33.86407691	8.269057179	2.034	0.00012046
AT3G60330	28.65106144	7.000508635	2.0331	0.00050481
AT5G60450	51.22711271	12.54453561	2.0298	1.06E–06
AT4G29800	23.31490386	5.73196009	2.0242	0.0022139
AT1G78580	52.54062842	12.92040184	2.0238	7.73E–07
AT3G17609	53.07424418	13.06135168	2.0227	6.73E–07
AT3G12610	50.36511803	12.40358577	2.0217	1.43E–06
AT5G10170	95.76350477	23.67957283	2.0158	5.83E–12
AT2G33620	35.62911365	8.832856532	2.0121	8.36E–05
AT2G28160	28.36372988	7.047491914	2.0089	0.00060778
AT1G72180	24.01270908	5.966876487	2.0088	0.0019464

AT3G06125	35.54701892	8.832856532	2.0088	8.71E–05
AT1G56320	24.38213538	6.060843046	2.0082	0.0017646
AT1G06690	24.54632484	6.107826326	2.0068	0.0016983
AT5G65390	95.88664687	23.86750595	2.0063	6.50E–12
AT3G30180	21.50881976	5.356093855	2.0057	0.0037916
AT3G16770	153.9276223	38.38533929	2.0036	8.34E–19
AT5G46790	49.01055495	12.26263593	1.9988	2.47E–06
AT1G12040	52.00701267	13.0143684	1.9986	1.10E–06
AT3G63110	22.12453025	5.544026972	1.9966	0.0033267
AT4G11320	727.7697984	182.6709904	1.9942	2.63E–87
AT5G67210	26.35240894	6.624642399	1.992	0.0011054
AT2G01830	89.52430515	22.69292396	1.98	5.42E–11
AT1G60390	44.61848679	11.32297034	1.9784	9.26E–06
AT1G70230	27.95325622	7.094475194	1.9782	0.00077086
AT5G61820	231.4250493	58.77608256	1.9772	1.18E–27
AT3G16420	2029.176536	516.2992576	1.9746	1.68E–240
AT3G45650	50.89873379	12.96738512	1.9727	1.84E–06
AT5G22740	174.6975895	44.58713218	1.9702	7.05E–21
AT4G08930	40.96527122	10.47727131	1.9671	2.64E–05
AT2G26980	62.80246991	16.11526484	1.9624	8.54E–08
AT1G21440	64.40331718	16.53811436	1.9613	5.66E–08
AT1G53830	33.61779272	8.644923415	1.9593	0.00019194
AT3G23470	39.81594498	10.24235492	1.9588	3.76E–05
AT3G26570	22.45290918	5.77894337	1.958	0.0034876
AT5G52882	148.5504174	38.29137273	1.9559	1.12E–17
AT3G57040	28.938393	7.470341429	1.9537	0.00066776
AT1G08320	61.57104893	15.92733173	1.9507	1.33E–07
AT3G11410	81.52006879	21.09549246	1.9502	6.94E–10

AT2G27840	64.77274348	16.77303076	1.9492	5.81E–08
AT1G80690	22.65814601	5.872909928	1.9479	0.0034254
AT4G02330	38.78976083	10.0544218	1.9478	5.26E–05
AT3G43960	27.46068782	7.141458473	1.9431	0.0010185
AT5G53760	37.72252931	9.8195054	1.9417	7.20E–05
AT5G63380	32.42741911	8.456990297	1.939	0.00029176
AT5G03555	47.90227607	12.49755233	1.9384	5.23E–06
AT5G42030	27.66592465	7.235425032	1.935	0.0010004
AT4G36640	40.92422386	10.71218771	1.9337	3.31E–05
AT4G22120	65.42950133	17.14889699	1.9318	5.87E–08
AT3G45410	21.50881976	5.637993531	1.9317	0.0048676
AT4G29720	27.41964046	7.188441752	1.9315	0.0010802
AT3G61590	24.01270908	6.295759443	1.9313	0.0025921
AT3G61820	72.16126935	18.93426161	1.9302	1.02E–08
AT5G01340	33.28941379	8.738889974	1.9295	0.00024569
AT2G26355	55.8244177	14.65878318	1.9291	7.26E–07
AT1G11450	24.01270908	6.342742723	1.9206	0.0026926
AT1G76790	152.7372487	40.40562031	1.9184	9.24E–18
AT4G23670	1068.052462	283.0272753	1.916	3.83E–122
AT1G75820	58.32830702	15.45749893	1.9159	4.29E–07
AT3G16410	54.05938096	14.32990023	1.9155	1.30E–06
AT4G04810	25.36727216	6.765592238	1.9067	0.0020316
AT4G13550	30.29295608	8.081124062	1.9064	0.00059018
AT3G45160	80.08341098	21.37739214	1.9054	1.81E–09
AT4G13860	29.88248242	7.987157503	1.9035	0.00066275
AT1G33560	69.00062217	18.46442882	1.9019	3.21E–08
AT4G38080	312.5756918	83.77118722	1.8997	1.55E–35
AT4G15390	180.9778365	48.72166077	1.8932	1.23E–20

AT4G16890	24.42318274	6.57765912	1.8926	0.0027015
AT3G06035	56.85060185	15.31654909	1.8921	7.80E–07
AT2G22230	32.75579803	8.832856532	1.8908	0.00034397
AT3G53510	54.83928092	14.79973302	1.8896	1.33E–06
AT1G18880	77.90790058	21.04850918	1.8881	3.92E–09
AT1G01610	105.8611568	28.75376701	1.8804	3.62E–12
AT4G31840	26.80392997	7.282408311	1.88	0.001581
AT4G27654	55.12661248	14.98766614	1.879	1.36E–06
AT5G26260	461.6186775	125.5863059	1.878	1.28E–51
AT1G80050	31.56542442	8.597940135	1.8763	0.00049681
AT1G49860	80.53493201	21.94119149	1.876	2.35E–09
AT1G59970	29.26677193	7.987157503	1.8735	0.00088294
AT5G66040	228.592781	62.44077836	1.8722	1.26E–25
AT1G65690	37.80462405	10.33632147	1.8708	0.00010848
AT2G24270	32.63265593	8.926823091	1.8701	0.00039487
AT1G05710	39.89803971	10.94710411	1.8658	6.65E–05
AT3G20015	29.75934032	8.17509062	1.864	0.00081879
AT5G10280	25.94193528	7.141458473	1.861	0.0021189
AT4G31730	27.74801939	7.658274547	1.8573	0.0013783
AT5G34940	30.29295608	8.363023738	1.8569	0.00074326
AT5G46900	34.64397687	9.584589003	1.8538	0.00026356
AT3G54810	234.9961701	65.11882529	1.8515	5.51E–26
AT1G68850	48.47693919	13.43721792	1.8511	8.88E–06
AT4G27595	30.66238237	8.503973576	1.8503	0.00070323
AT5G36160	51.7196811	14.3768835	1.847	4.14E–06
AT2G01890	86.6920369	24.24337218	1.8383	8.46E–10
AT2G41820	39.98013444	11.1820205	1.8381	7.76E–05
AT1G22550	49.99569173	14.00101727	1.8363	6.88E–06

AT1G30270	152.3267751	42.66081772	1.8362	7.86E–17
AT2G02680	41.58098171	11.6518533	1.8354	5.36E–05
AT1G18980	23.2738565	6.53067584	1.8334	0.0044558
AT1G18660	106.5179147	30.02231555	1.827	7.71E–12
AT5G08330	34.06931374	9.631572283	1.8226	0.00035841
AT5G66280	53.15633891	15.03464942	1.822	3.62E–06
AT3G56240	245.8326747	69.58223683	1.8209	1.26E–26
AT3G19030	320.1284071	90.77169585	1.8183	1.55E–34
AT1G80870	25.49041426	7.235425032	1.8168	0.0028115
AT1G78060	79.67293732	22.64594068	1.8148	6.36E–09
AT3G19370	184.0974363	52.38635656	1.8132	6.14E–20
AT3G27150	67.81024856	19.31012784	1.8121	1.17E–07
AT5G40830	30.621335	8.738889974	1.809	0.00086603
AT4G02970	60.70905425	17.33683011	1.8081	6.68E–07
AT4G34260	55.9475598	16.02129828	1.8041	2.17E–06
AT1G80280	38.05090824	10.90012083	1.8036	0.000154
AT2G04780	114.2758668	32.74734576	1.8031	1.81E–12
AT2G34420	25.57250899	7.329391591	1.8028	0.002916
AT3G51950	162.4654745	46.65439647	1.8	1.68E–17
AT5G25810	33.61779272	9.678555562	1.7964	0.00045926
AT5G11790	137.9191496	39.84182095	1.7915	7.69E–15
AT1G75780	70.56042208	20.39074327	1.7909	7.67E–08
AT2G46680	40.80108176	11.79280314	1.7907	8.70E–05
AT3G19680	151.3826856	43.83539971	1.788	3.28E–16
AT3G16240	155.8978959	45.15093153	1.7878	1.12E–16
AT1G53300	86.73308426	25.13605449	1.7868	1.74E–09
AT3G28200	52.82795999	15.31654909	1.7862	5.29E–06
AT2G18570	27.78906675	8.081124062	1.7819	0.0019161

AT2G02130	368.6463936	107.5447266	1.7773	1.25E–38
AT1G19230	26.92707207	7.893190944	1.7704	0.0024497
AT5G05960	83.81872128	24.6662217	1.7647	4.68E–09
AT3G26520	1014.157271	298.5317575	1.7643	5.87E–105
AT5G07030	178.6381366	52.62127296	1.7633	1.01E–18
AT5G56090	70.60146944	20.86057607	1.7589	1.10E–07
AT5G62220	48.96950759	14.47085006	1.7587	1.64E–05
AT5G17820	175.8879631	52.01049033	1.7578	2.25E–18
AT5G20110	25.73669845	7.611291267	1.7576	0.0033703
AT3G14680	85.41956855	25.27700433	1.7567	3.60E–09
AT1G56010	38.37928717	11.36995362	1.7551	0.00019283
AT2G05920	382.5204033	113.5585864	1.7521	2.25E–39
AT1G63000	226.4172706	67.32703942	1.7497	2.12E–23
AT4G13010	127.1647397	37.82153994	1.7494	2.46E–13
AT1G04680	94.0395154	28.00203454	1.7477	5.59E–10
AT5G08260	36.86053463	10.99408739	1.7453	0.00029073
AT1G36160	825.6677662	246.3803173	1.7447	2.59E–84
AT5G56530	40.96527122	12.26263593	1.7401	0.0001172
AT1G25520	42.15564484	12.63850217	1.7379	9.06E–05
AT5G04830	169.3203846	50.78892506	1.7372	1.85E–17
AT3G61270	38.05090824	11.4169369	1.7368	0.00023375
AT4G34610	27.05021416	8.128107341	1.7346	0.0027722
AT4G23510	25.94193528	7.799224385	1.7339	0.0035436
AT4G04640	27.50173519	8.269057179	1.7337	0.0025243
AT5G60660	59.02611224	17.75967962	1.7327	2.07E–06
AT1G23030	69.37004847	20.90755935	1.7303	2.01E–07
AT2G01520	383.0950665	115.4849008	1.73	7.98E–39
AT5G10430	171.0033266	51.58764081	1.7289	1.59E–17

AT2G37180	125.3586556	37.86852322	1.727	5.98E–13
AT4G26220	57.67154917	17.43079667	1.7262	2.99E–06
AT1G55210	115.302051	34.90857661	1.7238	6.31E–12
AT5G12420	112.1824512	34.01589431	1.7216	1.34E–11
AT1G45201	113.4959669	34.43874382	1.7205	1.01E–11
AT5G06390	96.95387839	29.4585162	1.7186	4.53E–10
AT5G59050	26.51659841	8.081124062	1.7143	0.0033921
AT1G30840	41.29365015	12.59151889	1.7135	0.00012993
AT2G14890	315.3258653	96.45667267	1.7089	1.57E–31
AT1G04250	122.0748664	37.39869042	1.7067	1.91E–12
AT5G23920	31.27809286	9.584589003	1.7064	0.0012373
AT3G57540	28.8152509	8.832856532	1.7059	0.0021424
AT2G47400	87.18460529	26.73348599	1.7054	4.97E–09
AT5G46110	26.22926685	8.081124062	1.6985	0.0038524
AT2G24580	43.14078162	13.29626808	1.698	9.57E–05
AT3G16180	30.45714554	9.396655886	1.6966	0.0015619
AT4G14760	81.52006879	25.18303777	1.6947	2.03E–08
AT1G20950	157.1703642	48.62769421	1.6925	9.78E–16
AT4G01450	88.53916837	27.48521847	1.6877	4.75E–09
AT3G63120	67.89234329	21.09549246	1.6863	4.57E–07
AT1G33800	74.09049555	23.0687902	1.6833	1.23E–07
AT1G41830	180.2389839	56.23898547	1.6803	8.13E–18
AT1G64370	152.6962014	47.68802862	1.679	3.79E–15
AT1G09560	508.4126747	158.8504677	1.6783	1.60E–49
AT1G70850	1059.51461	331.23212	1.6775	5.56E–103
AT1G44575	29.10258246	9.114756209	1.6749	0.0023251
AT1G51850	42.81240269	13.4842012	1.6668	0.00012827
AT1G77690	132.8703236	41.90908525	1.6647	4.21E–13

AT5G58900	48.68217602	15.36353237	1.6639	3.71E–05
AT2G47800	131.3926184	41.48623573	1.6632	5.99E–13
AT3G27390	82.50520557	26.21666992	1.654	2.85E–08
AT2G21050	30.8676192	9.8195054	1.6524	0.0017822
AT3G49670	28.938393	9.208722768	1.6519	0.0026714
AT3G11430	37.18891356	11.83978642	1.6512	0.00047385
AT5G57350	56.35803346	17.94761274	1.6508	7.96E–06
AT3G30875	29.0615351	9.255706047	1.6507	0.0026224
AT2G30520	160.4131062	51.21177458	1.6472	1.58E–15
AT5G18270	73.72106926	23.53862299	1.647	2.04E–07
AT3G27060	38.2150977	12.21565265	1.6454	0.00039525
AT5G44510	26.72183524	8.550956856	1.6439	0.0043845
AT5G27420	74.62411131	23.91448923	1.6418	1.80E–07
AT2G35840	39.69280288	12.73246872	1.6404	0.00029975
AT1G63940	249.116464	79.91855831	1.6402	9.72E–24
AT2G43360	74.74725341	24.00845579	1.6385	1.82E–07
AT1G78100	55.7012756	17.90062946	1.6377	1.03E–05
AT1G79270	192.7584305	62.06491212	1.6349	2.17E–18
AT2G23540	157.0061748	50.55400866	1.6349	4.58E–15
AT1G78340	65.51159607	21.09549246	1.6348	1.35E–06
AT3G01860	27.95325622	9.02078965	1.6317	0.0035744
AT4G00040	54.55194936	17.61872979	1.6305	1.41E–05
AT2G30200	73.84421135	23.86750595	1.6294	2.47E–07
AT2G28550	90.50944193	29.27058308	1.6286	7.29E–09
AT2G36290	251.5382586	81.65693965	1.6231	1.18E–23
AT5G27930	28.03535095	9.114756209	1.621	0.003688
AT2G33850	45.80886041	14.89369958	1.6209	9.45E–05
AT1G01630	77.41533219	25.18303777	1.6202	1.32E–07

AT5G49990	29.02048773	9.443639165	1.6197	0.0030453
AT5G61030	72.32545881	23.53862299	1.6195	3.82E–07
AT3G03520	73.10535877	23.86750595	1.6149	3.43E–07
AT5G02270	604.01199	197.235807	1.6147	5.23E–56
AT2G43800	37.31205565	12.21565265	1.6109	0.00058946
AT2G18980	96.78968892	31.71371361	1.6097	2.65E–09
AT3G12110	53.19738628	17.43079667	1.6097	2.24E–05
AT3G26650	76.84066907	25.27700433	1.604	1.80E–07
AT4G02280	63.82865406	21.0015259	1.6037	2.67E–06
AT2G33480	30.94971393	10.19537164	1.602	0.0022505
AT4G30020	59.51868064	19.68599408	1.5962	6.91E–06
AT1G78140	30.8676192	10.24235492	1.5915	0.0024099
AT2G46535	51.63758637	17.14889699	1.5903	3.65E–05
AT2G35380	49.74940754	16.53811436	1.5889	5.39E–05
AT2G36080	27.41964046	9.114756209	1.5889	0.004834
AT1G68560	127.7394029	42.51986788	1.587	6.62E–12
AT5G24030	39.36442395	13.10833496	1.5864	0.00045221
AT5G59090	236.55597	78.7909596	1.5861	1.26E–21
AT4G40060	119.2425981	39.84182095	1.5815	4.25E–11
AT4G34138	177.4067157	59.3868652	1.5788	3.01E–16
AT4G27860	88.86754729	29.78739916	1.577	2.23E–08
AT2G23340	35.83435048	12.02771953	1.575	0.00097307
AT3G02230	381.1658403	127.9354699	1.575	2.36E–34
AT4G23400	176.3805315	59.19893208	1.575	4.14E–16
AT2G04160	44.78267626	15.03464942	1.5746	0.00016452
AT4G36190	28.938393	9.725538842	1.5731	0.0038282
AT2G44940	37.96881351	12.779452	1.571	0.000658
AT3G16430	129.9149133	43.78841643	1.5689	6.27E–12

AT5G57070	75.19877443	25.37097089	1.5675	3.98E–07
AT1G36060	34.23350321	11.55788674	1.5665	0.0014019
AT4G39350	190.2545412	64.36709282	1.5635	3.50E–17
AT4G37150	43.83858684	14.8467163	1.5621	0.00021883
AT4G25630	166.0365953	56.23898547	1.5619	4.97E–15
AT5G02480	162.0960482	54.97043693	1.5601	1.15E–14
AT5G55550	36.94262936	12.54453561	1.5582	0.00086442
AT1G31770	61.94047523	21.09549246	1.5539	6.46E–06
AT3G15020	41.74517118	14.23593367	1.5521	0.00035371
AT2G36380	340.0774269	116.1426667	1.55	4.61E–30
AT1G61580	59.559728	20.34375999	1.5498	1.08E–05
AT4G34620	99.00624669	33.82796119	1.5493	4.49E–09
AT5G42825	35.46492419	12.12168609	1.5488	0.0012154
AT4G31920	43.79753947	14.98766614	1.5471	0.0002457
AT2G29750	59.02611224	20.20281015	1.5468	1.23E–05
AT5G65310	195.5496514	66.99815646	1.5453	2.17E–17
AT3G14350	107.338862	36.88187435	1.5412	9.91E–10
AT5G25220	35.99853994	12.40358577	1.5372	0.0011731
AT5G66052	46.54771299	16.06828156	1.5345	0.00015659
AT4G00030	32.63265593	11.27598706	1.5331	0.002303
AT5G63800	38.3382398	13.2492848	1.5329	0.00077024
AT3G21190	58.04097546	20.1088436	1.5292	1.77E–05
AT4G22130	74.50096921	25.84080369	1.5276	7.46E–07
AT4G01660	95.14779429	33.02924544	1.5264	1.37E–08
AT3G24190	115.8356667	40.21768719	1.5262	2.47E–10
AT1G28290	810.726525	281.7117434	1.525	8.00E–70
AT4G29270	78.68780054	27.34426863	1.5249	3.43E–07
AT1G30120	155.4874222	54.1247379	1.5224	1.17E–13

AT1G56550	33.86407691	11.79280314	1.5218	0.0019373
AT5G10480	92.68495233	32.27751297	1.5218	2.39E–08
AT3G52960	97.65168361	34.01589431	1.5214	9.09E–09
AT1G66400	44.08487104	15.36353237	1.5208	0.00028066
AT5G13650	183.4817258	64.03820986	1.5186	5.44E–16
AT2G40080	234.9551227	82.07978916	1.5173	2.55E–20
AT5G60120	49.05160232	17.19588027	1.5122	0.00011518
AT1G47670	54.18252306	19.02822817	1.5097	4.44E–05
AT2G17630	32.50951384	11.4169369	1.5097	0.0026575
AT1G22440	164.3126059	57.7894337	1.5076	3.13E–14
AT1G34000	33.69988745	11.8867697	1.5034	0.0022054
AT5G65640	66.00416446	23.3037066	1.502	5.08E–06
AT5G59450	54.75718619	19.35711112	1.5002	4.34E–05
AT2G43290	113.0444458	39.98277079	1.4994	7.01E–10
AT3G55360	84.06500548	29.83438244	1.4945	1.84E–07
AT1G14480	47.65599187	16.91398059	1.4944	0.00017302
AT1G29025	38.42033453	13.67213431	1.4906	0.00098334
AT5G54770	81.56111615	29.08264997	1.4877	3.24E–07
AT2G39890	32.38637174	11.55788674	1.4865	0.0030586
AT1G12140	68.05653275	24.29035546	1.4864	4.11E–06
AT1G65510	33.69988745	12.02771953	1.4864	0.0024171
AT3G26770	36.32691887	12.96738512	1.4862	0.001489
AT5G40240	33.65884008	12.02771953	1.4846	0.002456
AT1G15290	42.03250274	15.03464942	1.4832	0.00053389
AT4G35300	76.18391121	27.25030207	1.4832	9.39E–07
AT3G54260	55.37289667	19.8739272	1.4783	4.70E–05
AT3G49780	223.0103392	80.05950814	1.478	1.13E–18
AT1G18670	32.30427701	11.60487002	1.477	0.0032587

AT5G66310	34.93130843	12.59151889	1.4721	0.0020928
AT5G62440	56.93269658	20.53169311	1.4714	3.76E–05
AT5G39850	30.33400344	10.94710411	1.4704	0.0048146
AT2G48030	39.77489761	14.3768835	1.4681	0.00088467
AT5G44400	73.14640613	26.45158632	1.4674	2.01E–06
AT5G63760	32.71475067	11.83978642	1.4663	0.0031975
AT2G24570	58.61563858	21.2364423	1.4647	2.94E–05
AT4G17770	45.60362358	16.53811436	1.4634	0.00032125
AT1G58080	84.72176333	30.72706475	1.4632	2.52E–07
AT1G11840	548.8443302	199.2560881	1.4618	8.35E–45
AT4G13290	36.61425043	13.29626808	1.4614	0.0016399
AT1G52280	45.27524465	16.4441478	1.4611	0.000346
AT2G46650	64.11598562	23.3037066	1.4601	1.12E–05
AT1G07870	47.65599187	17.33683011	1.4588	0.00022956
AT1G27930	38.17405034	14.00101727	1.4471	0.0013477
AT5G57740	39.85699234	14.65878318	1.4431	0.0010237
AT5G62020	46.34247616	17.05493043	1.4421	0.00032888
AT4G39955	56.0707019	20.67264295	1.4395	5.88E–05
AT5G10100	42.52507113	15.69241533	1.4382	0.00066465
AT5G45100	37.68148195	13.90705071	1.438	0.0015576
AT5G47560	65.3474066	24.14940563	1.4361	1.16E–05
AT1G77510	71.54555886	26.45158632	1.4355	3.89E–06
AT1G76930	834.9444709	309.1499786	1.4334	2.49E–66
AT2G36830	588.9065594	218.0963831	1.4331	7.70E–47
AT3G52430	102.0847991	37.82153994	1.4325	1.73E–08
AT3G54400	76.75857434	28.47186733	1.4308	1.64E–06
AT2G36910	156.0620854	57.97736681	1.4286	1.19E–12
AT2G24300	34.11036111	12.68548545	1.427	0.0030885

AT5G21020	266.5205472	99.46360255	1.422	3.77E–21
AT2G27000	36.61425043	13.67213431	1.4212	0.0020818
AT2G21970	90.6736314	33.87494447	1.4205	1.60E–07
AT1G54030	99.58090981	37.21075731	1.4201	3.31E–08
AT1G06430	78.31837424	29.27058308	1.4199	1.42E–06
AT1G23330	50.61140222	18.93426161	1.4185	0.00018637
AT4G37540	60.99638581	22.8338738	1.4175	3.04E–05
AT3G55230	59.19030171	22.22309117	1.4133	4.35E–05
AT5G66570	62.43304362	23.49163971	1.4102	2.55E–05
AT5G27950	68.38491168	25.74683713	1.4093	9.12E–06
AT1G30400	336.6294482	126.8548545	1.408	2.81E–26
AT4G04830	300.1383399	113.1357369	1.4076	1.82E–23
AT2G26900	56.93269658	21.4713587	1.4068	6.82E–05
AT2G45970	43.42811318	16.39716452	1.4052	0.00071201
AT3G62830	334.700222	126.5259715	1.4034	5.04E–26
AT4G14130	87.59507895	33.123212	1.403	3.53E–07
AT2G04030	177.5709051	67.23307286	1.4012	5.58E–14
AT1G66200	771.4031484	292.3769479	1.3997	2.08E–59
AT1G10120	62.84351728	23.82052267	1.3996	2.65E–05
AT5G49810	144.5277755	54.87647037	1.3971	1.98E–11
AT5G20960	423.649864	161.246615	1.3936	1.45E–32
AT1G19140	56.89164921	21.65929182	1.3932	7.78E–05
AT1G67730	138.3706706	52.80920608	1.3897	6.83E–11
AT3G08030	94.08056277	35.98919204	1.3863	1.48E–07
AT1G47480	59.8060122	22.92784036	1.3832	5.23E–05
AT3G05490	126.4258871	48.53372765	1.3812	6.45E–10
AT3G56370	47.94332344	18.41744554	1.3803	0.00039936
AT1G69252	58.53354385	22.55197413	1.376	6.93E–05

AT5G24165	69.53423793	26.82745255	1.374	1.12E–05
AT5G08710	35.05445053	13.53118448	1.3733	0.0035315
AT3G18130	102.1258465	39.46595472	1.3717	4.78E–08
AT4G31990	167.7605847	64.83692561	1.3715	7.07E–13
AT2G30970	89.03173676	34.43874382	1.3703	4.42E–07
AT5G63590	34.4797874	13.34325136	1.3696	0.0039587
AT1G20225	68.22072222	26.45158632	1.3669	1.51E–05
AT1G64330	89.72954198	34.81461006	1.3659	4.19E–07
AT5G49350	37.35310302	14.51783334	1.3634	0.0025828
AT1G05570	276.5361044	107.5917099	1.3619	9.70E–21
AT5G53460	1223.539884	476.175537	1.3615	9.10E–91
AT2G16400	41.49888698	16.16224812	1.3604	0.0013304
AT5G12900	49.7904549	19.4040944	1.3595	0.00034651
AT5G54160	383.5876349	149.5007951	1.3594	1.47E–28
AT4G21960	1453.815607	566.9472329	1.3586	1.65E–107
AT3G01220	35.17759262	13.71911759	1.3585	0.0037603
AT4G20260	650.6007504	253.897642	1.3575	3.74E–48
AT4G30080	39.93908708	15.59844877	1.3564	0.0017713
AT4G29100	71.05299047	27.81410142	1.3531	1.10E–05
AT5G07580	76.51229014	29.97533227	1.3519	4.54E–06
AT4G03200	59.6828701	23.39767315	1.351	7.27E–05
AT1G50750	46.71190246	18.32347898	1.3501	0.00061581
AT3G56170	46.1782867	18.13554586	1.3484	0.00067851
AT5G03190	37.31205565	14.65878318	1.3479	0.0028423
AT1G20560	44.1259184	17.33683011	1.3478	0.00094607
AT3G55010	47.82018134	18.79331177	1.3474	0.00052505
AT2G16720	35.25968736	13.90705071	1.3422	0.0040663
AT1G17220	72.32545881	28.56583389	1.3402	1.04E–05

AT4G13930	567.110408	224.2511927	1.3385	3.26E–41
AT5G10580	70.10890105	27.81410142	1.3338	1.60E–05
AT5G24660	63.82865406	25.32398761	1.3337	4.43E–05
AT2G03480	39.77489761	15.83336517	1.3289	0.0021656
AT5G01890	75.2398218	29.97533227	1.3277	7.49E–06
AT5G37790	46.1782867	18.41744554	1.3261	0.0007975
AT2G46700	45.39838675	18.13554586	1.3238	0.00091731
AT2G46750	120.3098296	48.06389485	1.3237	5.64E–09
AT1G04040	120.4329717	48.25182797	1.3196	5.99E–09
AT4G29700	63.86970143	25.60588729	1.3187	5.12E–05
AT5G65970	56.68641238	22.73990724	1.3178	0.00016187
AT3G61380	40.5137502	16.25621468	1.3174	0.0020775
AT3G02910	126.3437924	50.74194178	1.3161	2.51E–09
AT2G34710	69.53423793	27.95505126	1.3146	2.19E–05
AT1G28580	148.0988964	59.66876487	1.3115	8.49E–11
AT4G24240	54.38775989	21.94119149	1.3096	0.00025217
AT1G64440	47.94332344	19.35711112	1.3085	0.00069157
AT3G26590	36.98367673	14.94068286	1.3076	0.0037825
AT2G45400	102.4542254	41.39226918	1.3075	1.33E–07
AT4G37300	103.6035517	41.86210197	1.3074	1.11E–07
AT2G02120	47.20447085	19.12219473	1.3037	0.00080334
AT4G35040	64.32122245	26.07572008	1.3026	5.62E–05
AT3G63200	108.1598093	43.88238299	1.3015	5.98E–08
AT3G16470	328.6662592	133.3855303	1.301	3.85E–23
AT1G76090	129.1350133	52.43333984	1.3003	2.25E–09
AT3G46290	52.66377052	21.42437542	1.2976	0.00036519
AT1G43710	163.5737533	66.76324007	1.2928	1.18E–11
AT3G52500	49.29788651	20.20281015	1.287	0.0006655

AT5G48300	49.83150227	20.43772655	1.2858	0.00061961
AT3G23090	83.73662655	34.39176054	1.2838	3.49E–06
AT1G75270	121.1718243	49.80227619	1.2828	1.10E–08
AT4G01480	150.2333594	61.78301245	1.2819	1.25E–10
AT5G26820	63.82865406	26.2636532	1.2811	7.57E–05
AT3G06390	38.91290293	16.02129828	1.2803	0.0033292
AT5G57685	82.9567266	34.20382742	1.2782	4.23E–06
AT1G27200	47.04028139	19.4040944	1.2775	0.00099994
AT4G14960	485.220913	200.2427369	1.2769	3.57E–33
AT5G03300	203.5949351	84.10007018	1.2755	3.93E–14
AT5G40910	60.25753322	24.94812138	1.2722	0.00014312
AT4G30460	101.4280413	42.00305181	1.2719	2.79E–07
AT1G37130	354.8544787	147.0576646	1.2708	3.44E–24
AT1G15210	234.7498859	97.44332153	1.2685	4.10E–16
AT4G26130	45.80886041	19.02822817	1.2675	0.0012951
AT1G21310	6046.277005	2519.854226	1.2627	0
AT1G64950	75.81448492	31.61974705	1.2616	1.54E–05
AT3G23640	89.31906832	37.25774059	1.2614	2.05E–06
AT3G03780	507.0170643	211.6126905	1.2606	4.64E–34
AT5G10770	77.74371112	32.51242936	1.2577	1.21E–05
AT5G37770	215.9091449	90.30186306	1.2576	1.09E–14
AT3G24503	418.149517	174.9187493	1.2573	4.85E–28
AT5G20070	122.1569611	51.21177458	1.2542	1.67E–08
AT4G12390	43.71544474	18.37046226	1.2508	0.0020009
AT4G30440	312.2062655	131.2712827	1.2499	7.32E–21
AT3G12700	86.60994217	36.50600812	1.2464	3.79E–06
AT3G22850	167.2680163	70.61586898	1.2441	2.51E–11
AT2G46890	39.15918712	16.53811436	1.2436	0.004019

AT2G41220	395.7787025	167.636341	1.2394	4.77E–26
AT3G15820	71.1350852	30.16326539	1.2378	4.08E–05
AT2G26730	70.14994842	29.78739916	1.2357	4.81E–05
AT5G44480	49.21579178	20.90755935	1.2351	0.0010028
AT1G44170	115.4662404	49.097527	1.2337	6.71E–08
AT3G21690	137.3034391	58.40021633	1.2333	2.75E–09
AT3G19170	139.3558074	59.33988192	1.2317	2.12E–09
AT3G27300	96.2150258	40.96941966	1.2317	1.15E–06
AT3G55340	41.70412381	17.75967962	1.2316	0.0030218
AT1G53240	225.3910865	96.08080643	1.2301	7.12E–15
AT3G06930	38.54347663	16.4441478	1.2289	0.0048146
AT5G15350	199.2439143	85.08671904	1.2275	3.66E–13
AT1G26450	40.3906081	17.28984683	1.2241	0.0038056
AT2G37170	563.9497609	241.7289727	1.2222	2.66E–36
AT2G40840	75.97867438	32.60639592	1.2204	2.49E–05
AT3G19710	76.75857434	33.02924544	1.2166	2.33E–05
AT3G23600	284.1298671	122.2974764	1.2162	2.38E–18
AT1G80270	44.94686572	19.35711112	1.2154	0.002143
AT5G67470	65.42950133	28.23695094	1.2124	0.0001224
AT2G39900	55.9475598	24.14940563	1.2121	0.00047039
AT1G35320	44.29010787	19.12219473	1.2117	0.0024099
AT5G42180	45.48048148	19.6390108	1.2115	0.002048
AT3G05890	146.3338596	63.19251083	1.2114	1.23E–09
AT2G28720	59.88810693	25.88778696	1.21	0.00027619
AT1G51070	152.4909645	66.05849088	1.2069	5.71E–10
AT1G60950	76.67647961	33.21717856	1.2069	2.65E–05
AT4G15920	57.6305018	24.99510466	1.2052	0.00039525
AT1G64650	85.83004221	37.25774059	1.2039	7.54E–06

AT3G28550	989.4467564	429.6151071	1.2036	3.36E–62
AT5G56540	98.88310459	42.9427174	1.2033	1.23E–06
AT5G61790	458.3348883	199.2091048	1.2021	6.63E–29
AT3G22240	71.21717993	30.96198114	1.2017	6.06E–05
AT1G06550	344.1411162	149.6887283	1.201	9.29E–22
AT4G20270	66.94825387	29.17661652	1.1982	0.0001146
AT3G29360	203.7180772	88.79839812	1.198	5.04E–13
AT3G58550	60.99638581	26.63951944	1.1952	0.00027288
AT1G30690	156.9651274	68.59558796	1.1943	4.16E–10
AT3G05165	159.1406378	69.58223683	1.1935	3.11E–10
AT4G09670	53.44367047	23.39767315	1.1917	0.00078923
AT4G16340	126.7132187	55.53423628	1.1901	3.13E–08
AT4G26010	58.32830702	25.60588729	1.1877	0.00042082
AT4G24830	89.89373144	39.512938	1.1859	5.53E–06
AT5G37990	141.777602	62.3468118	1.1852	4.28E–09
AT5G35970	67.19453807	29.55248276	1.1851	0.00012746
AT5G26780	87.47193685	38.47930585	1.1847	7.83E–06
AT5G56000	67.85129592	29.88136572	1.1831	0.00011884
AT1G74030	197.3146881	86.91906694	1.1828	2.03E–12
AT1G54000	275.6741098	121.4987606	1.182	3.67E–17
AT1G14210	47.32761295	20.86057607	1.1819	0.0019706
AT5G42270	196.0011724	86.40225087	1.1817	2.51E–12
AT1G12000	237.007491	104.5377967	1.1809	8.51E–15
AT3G23490	76.55333751	33.78097791	1.1803	3.73E–05
AT1G78290	101.6743255	44.86903185	1.1802	1.20E–06
AT4G06744	143.009023	63.19251083	1.1783	4.24E–09
AT3G28860	145.0203439	64.08519314	1.1782	3.21E–09
AT1G33170	44.08487104	19.49806096	1.177	0.0031326

AT3G25530	82.01263718	36.27109172	1.177	1.84E–05
AT1G19700	44.45429733	19.68599408	1.1752	0.0030231
AT3G44320	149.0429858	66.15245743	1.1719	2.15E–09
AT2G05990	156.1852275	69.39430371	1.1704	8.41E–10
AT4G34135	83.49034235	37.11679075	1.1695	1.66E–05
AT5G09870	186.2729467	83.01945475	1.1659	1.55E–11
AT3G15530	84.02395811	37.49265698	1.1642	1.66E–05
AT5G28540	523.9696264	233.8357817	1.164	1.35E–31
AT5G48560	87.26670002	38.94913865	1.1638	1.08E–05
AT2G30860	1041.617958	465.2284329	1.1628	1.83E–62
AT2G43710	100.4839519	44.91601513	1.1617	1.91E–06
AT5G23890	185.5340941	82.97247147	1.161	1.98E–11
AT5G02050	66.66092231	29.83438244	1.1599	0.00017941
AT5G64030	97.61063624	43.69444987	1.1596	2.89E–06
AT4G17520	310.1128498	138.882574	1.1589	9.75E–19
AT4G13660	113.7832984	51.02384146	1.157	3.45E–07
AT1G62800	56.85060185	25.51192073	1.156	0.00068195
AT1G35720	1122.686506	504.0366217	1.1554	1.01E–66
AT4G19120	95.02465219	42.66081772	1.1554	4.33E–06
AT2G05940	164.887269	74.13961494	1.1532	4.04E–10
AT2G45470	70.31413788	31.71371361	1.1487	0.00012438
AT1G74770	78.85199	35.56634253	1.1486	4.04E–05
AT3G60750	434.3221792	196.2961414	1.1457	1.10E–25
AT5G47870	67.07139597	30.35119851	1.1439	0.00020116
AT5G56950	185.9445678	84.14705345	1.1439	3.12E–11
AT2G43150	2447.243958	1108.147629	1.143	3.03E–143
AT3G44100	71.05299047	32.23052969	1.1405	0.00012375
AT5G08240	109.7606566	49.80227619	1.1401	7.92E–07

AT4G36920	55.16765984	25.04208793	1.1395	0.0009755
AT2G21250	66.33254338	30.11628211	1.1392	0.0002336
AT2G39700	105.2864937	47.92294502	1.1355	1.54E–06
AT3G13690	49.54417071	22.55197413	1.1355	0.0020892
AT1G78610	43.14078162	19.6390108	1.1353	0.0047016
AT2G22970	47.32761295	21.56532526	1.134	0.002789
AT1G79530	231.0966703	105.6653954	1.129	1.34E–13
AT2G22170	210.2035611	96.22175627	1.1274	2.17E–12
AT2G01950	64.73169611	29.64644932	1.1266	0.00032604
AT2G12400	141.2439863	64.74295905	1.1254	1.76E–08
AT3G43600	100.9354729	46.27853024	1.125	3.21E–06
AT4G03020	181.5114523	83.25437115	1.1245	9.83E–11
AT1G53730	55.8244177	25.65287057	1.1218	0.001044
AT3G46700	106.8873409	49.19149356	1.1196	1.64E–06
AT4G17340	166.0365953	76.44179563	1.1191	8.46E–10
AT4G12470	50.61140222	23.3037066	1.1189	0.0020758
AT3G09390	257.1617477	118.4448474	1.1185	6.75E–15
AT4G05390	75.69134282	34.86159334	1.1185	8.75E–05
AT1G77590	78.19523215	36.03617532	1.1176	6.44E–05
AT1G23190	110.1711302	50.78892506	1.1172	1.12E–06
AT3G63410	47.28656558	21.80024165	1.1171	0.0031634
AT2G35940	63.6644646	29.41153292	1.1141	0.00042187
AT2G41475	65.79892763	30.39818179	1.1141	0.00032373
AT4G02540	66.82511178	30.91499786	1.1121	0.00029073
AT3G45600	97.28225732	45.00998169	1.1119	6.22E–06
AT1G01090	198.1766828	91.71136144	1.1116	1.70E–11
AT3G15950	1116.529401	516.863057	1.1112	6.78E–63
AT1G67030	50.73454432	23.49163971	1.1108	0.0021732

AT2G47180	54.46985462	25.23002105	1.1103	0.0013672
AT4G12420	178.4739472	82.69057179	1.1099	2.21E–10
AT1G48920	620.6361733	288.1014695	1.1072	6.62E–35
AT3G27820	120.4740191	56.00406908	1.1051	3.79E–07
AT3G54010	106.9283883	49.75529291	1.1037	2.13E–06
AT4G15160	54.26461779	25.27700433	1.1022	0.0015025
AT3G48115	49.70836017	23.16275676	1.1017	0.0026433
AT5G35460	65.30635924	30.49214835	1.0988	0.00040088
AT3G44590	55.49603877	25.98175352	1.0949	0.0013736
AT2G37220	192.0606253	89.9729801	1.094	6.32E–11
AT2G01140	499.8748226	234.3525978	1.0929	1.14E–27
AT5G46470	58.61563858	27.48521847	1.0926	0.00095671
AT3G53480	242.6309802	113.7935028	1.0923	1.22E–13
AT1G53280	79.63188995	37.39869042	1.0904	7.52E–05
AT5G46290	157.4576958	73.95168182	1.0903	5.19E–09
AT3G54040	105.65592	49.66132636	1.0892	3.17E–06
AT2G21790	65.96311709	31.00896442	1.089	0.00040838
AT1G15500	387.076661	182.1541743	1.0875	2.34E–21
AT3G09820	414.2910646	195.215526	1.0856	8.72E–23
AT3G13470	75.65029546	35.66030909	1.085	0.00013062
AT1G08190	199.3260091	94.01354213	1.0842	3.49E–11
AT4G23800	55.53708614	26.2636532	1.0804	0.0015505
AT5G56500	139.0274285	65.7765912	1.0797	6.40E–08
AT1G72130	49.05160232	23.20974004	1.0796	0.0033646
AT2G05380	65.26531187	30.91499786	1.078	0.00049661
AT4G10480	197.3557355	93.54370934	1.0771	5.54E–11
AT1G23820	143.009023	67.84385549	1.0758	4.29E–08
AT1G09850	56.23489136	26.68650271	1.0754	0.0014872

AT1G75840	86.4457527	41.06338622	1.0739	4.08E–05
AT3G52880	410.186328	194.9336263	1.0733	3.25E–22
AT1G74450	102.0847991	48.53372765	1.0727	6.34E–06
AT4G37520	236.2686384	112.3370211	1.0726	5.64E–13
AT1G19570	367.9485884	175.3885821	1.0689	7.31E–20
AT4G18040	75.03458497	35.8482422	1.0657	0.00017716
AT5G20885	57.42526497	27.48521847	1.063	0.001438
AT5G54800	110.6226513	52.95015591	1.0629	2.71E–06
AT1G04820	367.7023042	176.046348	1.0626	1.09E–19
AT1G56110	254.16529	121.733677	1.062	9.71E–14
AT1G47128	934.7716649	447.7976362	1.0618	1.75E–49
AT3G15353	92.64390496	44.39919906	1.0612	2.33E–05
AT3G53420	764.3840488	366.5635461	1.0602	1.97E–40
AT2G28890	60.17543849	28.94170013	1.056	0.0011073
AT5G64290	61.7352384	29.6934326	1.056	0.00092725
AT3G50530	64.44436455	31.10293098	1.051	0.00071303
AT3G02630	67.6460591	32.6533792	1.0508	0.00049539
AT4G13340	578.1931969	279.2686129	1.0499	2.96E–30
AT5G63980	56.89164921	27.48521847	1.0496	0.0017276
AT1G66330	47.90227607	23.16275676	1.0483	0.0048466
AT3G25860	133.2807973	64.50804265	1.0469	2.54E–07
AT2G26650	150.0691699	72.73011655	1.045	3.73E–08
AT1G33240	63.00770674	30.58611491	1.0427	0.00090937
AT3G48690	110.9099828	53.93680478	1.04	3.86E–06
AT2G21660	1731.952559	842.4571834	1.0397	5.63E–89
AT3G16400	780.556711	379.7658476	1.0394	3.64E–40
AT2G06850	59.35449117	28.89471685	1.0386	0.0014314
AT1G20620	500.9420541	243.9841701	1.0379	7.74E–26

AT1G14120	51.88387057	25.27700433	1.0375	0.0033456
AT5G15490	93.62904175	45.62076432	1.0373	2.93E–05
AT3G06650	221.8199656	108.108526	1.0369	1.12E–11
AT2G10410	181.5524996	88.51649844	1.0364	1.21E–09
AT3G07460	51.92491793	25.32398761	1.0359	0.0033703
AT2G42490	248.8701798	121.404794	1.0356	5.14E–13
AT4G12600	87.63612632	42.75478428	1.0354	5.93E–05
AT3G59970	271.8977521	132.7747477	1.0341	3.78E–14
AT5G51040	220.6706394	107.779643	1.0338	1.42E–11
AT4G08390	178.7202314	87.38889974	1.0322	1.89E–09
AT4G21910	49.37998124	24.14940563	1.0319	0.0046278
AT1G48480	51.47339691	25.18303777	1.0314	0.0036679
AT4G29680	74.37782711	36.50600812	1.0267	0.00029964
AT3G09980	88.58021573	43.55350003	1.0242	6.20E–05
AT1G62640	68.50805378	33.68701135	1.0241	0.00059257
AT3G01930	52.86900735	26.0287368	1.0223	0.0033777
AT1G05000	55.12661248	27.15633551	1.0215	0.0026658
AT1G65010	122.7316242	60.5144639	1.0202	1.44E–06
AT5G67420	78.7288479	38.90215537	1.017	0.00020585
AT3G01720	128.3961607	63.47441051	1.0164	8.18E–07
AT5G58300	51.76072847	25.60588729	1.0154	0.0040211
AT3G62130	50.89873379	25.18303777	1.0152	0.004439
AT3G21220	49.913597	24.71320498	1.0142	0.0049808
AT5G54960	138.8221917	68.7365378	1.0141	2.69E–07
AT5G52540	56.44012819	27.95505126	1.0136	0.0024664
AT5G13420	418.4368486	207.6191118	1.0111	7.03E–21
AT5G58620	103.8087885	51.54065753	1.0101	1.40E–05
AT2G28000	173.7535001	86.30828431	1.0095	6.07E–09

AT3G46560	61.44790684	30.53913163	1.0087	0.001489
AT3G15090	57.5073597	28.61281717	1.0071	0.0023251
AT3G45310	55.7012756	27.81410142	1.0019	0.0029341
AT1G45160	66.53778021	33.26416183	1.0002	0.00093087
AT5G11740	280.1072253	562.2958882	–1.0054	1.61E–15
AT3G06860	130.4074816	261.9787661	–1.0064	1.61E–07
AT5G57560	407.6003439	819.4353765	–1.0075	1.68E–22
AT4G33940	45.19314992	90.91264569	–1.0084	0.0049566
AT1G17290	129.4223449	260.8041841	–1.0109	1.50E–07
AT1G71880	152.5730593	307.5525471	–1.0113	8.12E–09
AT3G06380	45.76781304	92.41611063	–1.0138	0.0042602
AT2G03730	51.88387057	104.8666797	–1.0152	0.0019873
AT1G45145	213.8567766	432.6690202	–1.0166	2.63E–12
AT2G48020	45.97304987	93.02689327	–1.0169	0.0039606
AT1G73500	77.33323746	156.6422536	–1.0183	7.76E–05
AT1G03290	48.60008129	98.47695368	–1.0188	0.0027918
AT4G30920	63.4181804	128.5932358	–1.0198	0.00043463
AT5G58020	54.67509145	110.974506	–1.0213	0.0012529
AT5G05410	201.4604721	409.0364307	–1.0217	8.96E–12
AT3G27260	81.76635298	166.1328761	–1.0228	3.95E–05
AT4G22330	70.68356418	143.8158183	–1.0248	0.0001542
AT5G62640	45.07000782	91.71136144	–1.0249	0.003929
AT1G19770	262.9494263	535.2805025	–1.0255	2.27E–15
AT4G03390	48.18960763	98.14807072	–1.0262	0.0026199
AT5G53310	49.09264968	100.0743852	–1.0275	0.0022927
AT3G05545	70.80670627	144.4735842	–1.0288	0.00013761
AT3G10300	76.88171644	157.0651031	–1.0307	5.97E–05
AT5G44290	101.3048992	207.8070449	–1.0365	2.05E–06

AT1G25682	47.40970768	97.39633825	–1.0387	0.0023775
AT1G14900	52.13015476	107.1218771	–1.0391	0.0012627
AT5G25350	83.69557918	172.1937191	–1.0408	1.84E–05
AT2G44380	46.67085509	96.08080643	–1.0417	0.0024882
AT3G03790	41.78621854	86.07336791	–1.0425	0.0046596
AT4G27960	132.0493763	271.9862046	–1.0425	2.63E–08
AT1G12200	55.9475598	115.2499844	–1.0426	0.00071689
AT5G53550	51.76072847	106.6520443	–1.043	0.0012368
AT1G08940	42.11459747	87.2479499	–1.0508	0.0039448
AT4G31860	68.13862749	141.2787212	–1.052	0.00011286
AT2G22010	60.13439113	124.6936236	–1.0521	0.00033929
AT3G62240	43.42811318	90.2078965	–1.0546	0.0031313
AT4G17140	137.7960075	286.7389543	–1.0572	5.83E–09
AT5G65630	195.1802251	406.9221831	–1.0599	1.49E–12
AT2G45000	40.30851337	84.19403673	–1.0626	0.0042699
AT3G50930	167.9247741	350.7771642	–1.0627	6.01E–11
AT1G33980	56.0707019	117.4581986	–1.0668	0.00044118
AT4G22820	60.21648586	126.2910551	–1.0685	0.00023661
AT5G50960	51.9659653	109.3770745	–1.0737	0.00069068
AT4G33430	116.8618509	246.0044511	–1.0739	5.51E–08
AT4G16760	216.1143818	455.5028941	–1.0757	2.15E–14
AT3G02750	68.46700641	144.3796177	–1.0764	5.92E–05
AT1G28200	70.72461154	149.5947617	–1.0808	3.81E–05
AT1G78420	56.93269658	120.7000448	–1.0841	0.0002741
AT4G01000	127.3699766	270.0598902	–1.0843	7.13E–09
AT1G69840	38.42033453	81.46900653	–1.0844	0.0041137
AT1G02360	49.09264968	104.3498636	–1.0878	0.00080893
AT5G42570	82.66939504	175.7644483	–1.0882	5.17E–06

AT3G51130	42.93554479	91.33549521	–1.089	0.0019923
AT2G04400	51.18606535	109.2361247	–1.0936	0.00053343
AT3G25730	39.32337659	83.95912034	–1.0943	0.0031305
AT1G62380	150.9311646	322.7751297	–1.0966	1.02E–10
AT2G02860	50.89873379	109.0481916	–1.0993	0.00050019
AT3G12140	40.92422386	87.76476597	–1.1007	0.0022456
AT1G64280	53.36157574	114.498252	–1.1015	0.00032867
AT2G39570	244.2318274	525.3200473	–1.1049	2.16E–17
AT1G67580	62.80246991	135.1239116	–1.1054	6.86E–05
AT5G26760	35.50597155	76.39481235	–1.1054	0.0047971
AT1G62570	104.5065937	225.143875	–1.1073	9.03E–08
AT4G05070	38.95395029	84.00610362	–1.1087	0.0026893
AT2G42680	116.3282351	250.8907121	–1.1089	1.27E–08
AT3G45980	122.4442926	264.2809468	–1.1099	4.57E–09
AT4G12040	131.7620447	284.4837569	–1.1104	1.00E–09
AT2G20900	52.54062842	113.6055697	–1.1125	0.00029955
AT3G18830	74.17259028	160.5888491	–1.1144	8.89E–06
AT2G03240	143.3374019	310.6534436	–1.1159	1.14E–10
AT3G48360	174.5744474	378.4503158	–1.1163	6.81E–13
AT4G18710	157.252459	341.0986086	–1.1171	1.09E–11
AT4G25810	122.1980085	265.0796625	–1.1172	3.36E–09
AT1G29930	60.62695952	131.647149	–1.1186	7.07E–05
AT4G21660	174.9028263	381.6451788	–1.1257	3.34E–13
AT5G13800	43.46916055	94.85924116	–1.1258	0.00099679
AT5G56870	203.1434141	443.9450073	–1.1279	2.43E–15
AT5G51070	127.082645	277.8591145	–1.1286	8.54E–10
AT5G22450	83.98291075	183.7985891	–1.13	1.11E–06
AT5G44070	63.58236987	139.1644737	–1.1301	3.30E–05

AT5G57910	46.3014288	101.4838836	−1.1321	0.00056383
AT5G13160	39.32337659	86.26130103	−1.1333	0.0017547
AT5G15970	316.1468126	694.8827027	−1.1362	4.29E−24
AT3G52240	40.06222917	88.14063221	−1.1376	0.0014452
AT4G27520	109.2680882	240.4134408	−1.1376	1.13E−08
AT3G57280	42.0735501	92.60404375	−1.1382	0.0010191
AT3G63070	62.43304362	137.5200589	−1.1393	3.17E−05
AT5G43180	49.913597	110.128807	−1.1417	0.00025674
AT4G27585	34.64397687	76.48877891	−1.1426	0.0033233
AT1G75380	62.43304362	137.8959251	−1.1432	2.88E−05
AT5G08630	36.86053463	81.60995637	−1.1467	0.0021674
AT3G61070	40.1443239	88.89236468	−1.1469	0.0012242
AT4G33150	219.8086447	487.4045408	−1.1489	2.19E−17
AT5G51980	40.3906081	89.78504699	−1.1525	0.0010711
AT3G25900	33.24836642	74.04564838	−1.1551	0.0035532
AT5G49440	138.5348601	308.5861793	−1.1554	2.71E−11
AT5G52580	56.02965453	124.8815567	−1.1563	6.40E−05
AT5G55100	45.9320025	102.3765659	−1.1563	0.00038385
AT5G39660	39.98013444	89.12728107	−1.1566	0.0010751
AT1G01770	41.78621854	93.16784311	−1.1568	0.00078297
AT1G22020	34.80816633	77.66336089	−1.1578	0.0026276
AT5G27380	79.18036893	176.9390303	−1.16	9.33E−07
AT1G60940	80.32969518	179.7580271	−1.1621	7.09E−07
AT2G47160	40.43165547	90.58376274	−1.1638	0.00088356
AT5G57580	70.80670627	158.897451	−1.1661	3.49E−06
AT1G13880	33.37150852	75.1262638	−1.1707	0.0028219
AT1G47380	42.11459747	94.95320772	−1.1729	0.000562
AT5G18130	55.16765984	124.5526738	−1.1749	4.85E−05

AT4G37820	46.58876036	105.2425459	−1.1757	0.00023517
AT2G44080	33.53569799	75.87799628	−1.178	0.0024821
AT5G47120	127.0005503	287.3967203	−1.1782	6.24E−11
AT3G09440	780.3514742	1766.148457	−1.1784	3.75E−65
AT3G11340	45.48048148	102.9873485	−1.1791	0.00027023
AT2G19270	35.34178209	80.05950814	−1.1797	0.0017398
AT5G18650	70.72461154	160.5418658	−1.1827	2.17E−06
AT2G30250	51.76072847	117.693115	−1.1851	7.25E−05
AT1G53210	237.4179647	540.072797	−1.1857	2.34E−20
AT3G53110	96.87178366	220.8214133	−1.1887	1.17E−08
AT1G02170	40.76003439	92.93292671	−1.189	0.00054352
AT3G45960	67.56396436	154.0581732	−1.1891	3.25E−06
AT1G19180	291.4362983	664.5315042	−1.1892	4.23E−25
AT3G11420	86.19946851	197.1418405	−1.1935	7.70E−08
AT3G17000	50.69349696	116.0487002	−1.1949	7.16E−05
AT5G62460	36.16272941	82.78453835	−1.1949	0.0011752
AT5G50430	30.16981398	69.11240404	−1.1958	0.0036341
AT5G18470	46.75294982	107.2628269	−1.198	0.00014458
AT1G16840	32.14008754	73.81073198	−1.1995	0.0023922
AT5G23830	44.45429733	102.1886328	−1.2008	0.00021513
AT1G73920	66.20940129	152.4137585	−1.2029	2.83E−06
AT5G50210	36.86053463	84.89878593	−1.2037	0.00089352
AT1G55810	82.7104424	190.6581479	−1.2048	1.00E−07
AT3G26020	37.64043458	86.7781171	−1.205	0.00075115
AT5G23050	107.8314304	248.7294813	−1.2058	6.35E−10
AT5G16630	28.24058778	65.16580857	−1.2063	0.0046278
AT5G44090	63.37713304	146.3059321	−1.207	4.39E−06
AT5G41100	33.16627169	76.58274547	−1.2073	0.0017539

AT5G65660	115.6304299	267.005977	–1.2074	1.21E–10
AT3G54020	51.51444427	119.0086468	–1.208	4.54E–05
AT1G21450	61.57104893	142.5002865	–1.2106	5.67E–06
AT2G20560	33.65884008	77.89827729	–1.2106	0.0015094
AT4G33910	29.71829295	68.83050436	–1.2117	0.0032319
AT1G11380	27.66592465	64.13217642	–1.2129	0.0047808
AT1G61340	74.13154292	171.8648361	–1.2131	4.18E–07
AT2G33830	656.5526185	1523.761718	–1.2147	1.73E–59
AT4G21980	71.5045115	165.9919262	–1.215	6.71E–07
AT1G27300	54.92137565	127.6065869	–1.2163	1.88E–05
AT5G02490	34.64397687	80.52934094	–1.2169	0.0011228
AT1G04985	34.93130843	81.42202325	–1.2209	0.00099727
AT1G06760	197.6020197	460.7650213	–1.2214	2.13E–18
AT4G33540	33.53569799	78.36811009	–1.2246	0.001252
AT3G20060	27.37859309	63.99122658	–1.2248	0.0043745
AT5G13750	71.70974832	167.8242741	–1.2267	4.38E–07
AT5G16830	68.54910115	160.4478992	–1.2269	8.43E–07
AT4G19880	222.8051024	522.1721676	–1.2287	5.18E–21
AT1G30720	61.77628576	144.8024672	–1.229	3.28E–06
AT4G21570	92.15133657	216.1700686	–1.2301	5.25E–09
AT3G04350	54.34671253	127.5596036	–1.2309	1.47E–05
AT1G69830	33.33046116	78.27414353	–1.2317	0.0011729
AT1G59870	557.7516086	1310.927463	–1.2329	1.30E–52
AT1G01350	28.938393	68.03178861	–1.2332	0.0028564
AT5G10980	208.5206191	490.2705208	–1.2334	6.75E–20
AT1G22280	68.71329061	161.6224812	–1.234	6.53E–07
AT2G18440	140.5051337	330.9502203	–1.236	1.25E–13
AT2G31260	37.27100829	87.81174925	–1.2364	0.00048089

AT5G17380	106.2305831	250.6557957	–1.2385	1.76E–10
AT1G71697	55.12661248	130.143684	–1.2393	1.01E–05
AT1G31930	60.54486478	143.0640859	–1.2406	3.06E–06
AT3G62010	78.64675317	185.8658534	–1.2408	6.16E–08
AT3G02340	28.03535095	66.34039055	–1.2426	0.0030555
AT4G02380	355.8806628	844.571431	–1.2468	1.04E–34
AT5G14120	112.6339722	267.2878767	–1.2468	2.84E–11
AT1G14000	67.31768017	159.8840999	–1.248	5.62E–07
AT2G31955	26.14717211	62.15887868	–1.2493	0.0041909
AT1G01340	38.2150977	90.91264569	–1.2503	0.00030871
AT4G21390	30.33400344	72.30726704	–1.2532	0.0016243
AT5G09390	35.21863999	83.95912034	–1.2533	0.00056141
AT2G47730	529.0594998	1261.688986	–1.2539	3.10E–52
AT5G63320	63.99284353	152.6956581	–1.2547	9.50E–07
AT1G72416	34.35664531	81.9858226	–1.2548	0.00066147
AT2G46740	40.18537127	95.89287331	–1.2548	0.00018407
AT5G61440	29.22572456	69.77016995	–1.2554	0.0020103
AT2G38560	26.59869314	63.52139379	–1.2559	0.0035043
AT2G32240	179.3359419	429.098291	–1.2586	4.08E–18
AT1G56145	63.62341723	152.507725	–1.2613	8.43E–07
AT1G19020	105.5738252	253.1928928	–1.262	6.17E–11
AT4G36648	180.1158418	432.3401373	–1.2632	2.27E–18
AT3G58750	85.13223699	204.3772655	–1.2635	5.98E–09
AT5G26740	84.5165265	203.1557002	–1.2653	6.36E–09
AT4G05020	93.67008911	225.1908583	–1.2655	7.92E–10
AT1G61690	54.05938096	130.4255837	–1.2706	5.64E–06
AT1G23800	46.46561826	112.149088	–1.2712	3.17E–05
AT3G54620	94.24475223	227.493039	–1.2713	5.30E–10

ATCG01130	102.4952728	247.4609327	–1.2716	7.61E–11
AT4G20380	37.18891356	89.87901355	–1.2731	0.00025743
AT5G10400	33.82302955	81.84487276	–1.2749	0.00053662
AT1G30730	86.28156324	208.8406771	–1.2753	2.81E–09
AT2G23320	71.66870096	173.7441673	–1.2775	7.81E–08
AT2G39350	42.27878693	102.5175157	–1.2779	7.17E–05
AT2G46140	49.25683915	119.5254629	–1.2789	1.39E–05
AT3G22750	24.01270908	58.35323305	–1.281	0.0046278
AT2G23140	25.16203533	61.17222981	–1.2816	0.0035184
AT4G19860	99.66300454	242.5746717	–1.2833	8.24E–11
AT2G03470	35.87539785	87.38889974	–1.2845	0.00028494
AT4G21470	36.20377677	88.28158204	–1.286	0.00025674
AT2G02220	62.39199625	152.3197919	–1.2877	4.94E–07
AT4G30530	158.1144537	386.5784231	–1.2898	4.26E–17
AT5G62480	35.30073472	86.30828431	–1.2898	0.0002966
AT2G31800	70.39623261	172.1467358	–1.2901	6.78E–08
AT1G71950	55.78337033	136.4864267	–1.2909	2.19E–06
AT2G22470	203.5538878	498.6805278	–1.2927	5.32E–22
AT4G27450	54.05938096	132.492848	–1.2933	3.10E–06
AT2G43820	23.72537752	58.21228321	–1.2949	0.0041973
AT3G49790	40.3906081	99.18170287	–1.2961	7.72E–05
AT2G27550	40.59584493	99.98041862	–1.3003	6.74E–05
AT1G05960	75.1166797	185.5369705	–1.3045	1.25E–08
AT1G21680	45.02896045	111.303389	–1.3056	2.04E–05
AT4G39670	23.72537752	58.68211601	–1.3065	0.0036578
AT3G50950	66.49673285	164.6763944	–1.3083	9.33E–08
AT5G57015	34.31559794	84.99275248	–1.3085	0.00027159
AT4G19200	159.5100641	395.5052462	–1.31	5.81E–18

AT2G33700	37.72252931	93.6376759	–1.3117	0.00010913
AT3G45040	30.53924027	75.831013	–1.3121	0.00064643
AT2G18750	110.704746	275.2280509	–1.3139	1.06E–12
AT5G12480	70.10890105	174.4958998	–1.3155	2.90E–08
AT5G45190	55.24975458	137.5200589	–1.3156	1.24E–06
AT4G24590	33.53569799	83.48928754	–1.3159	0.00028952
AT1G55450	50.81663905	126.6669213	–1.3177	3.57E–06
AT3G52060	74.8703955	186.6645692	–1.318	7.80E–09
AT5G23340	27.46068782	68.54860468	–1.3198	0.0012292
AT4G15420	23.1507144	57.88340025	–1.3221	0.0035025
AT1G42990	82.05368455	205.2229645	–1.3226	1.04E–09
AT3G19190	59.6828701	149.5007951	–1.3248	3.04E–07
AT3G03310	32.34532437	81.09314029	–1.326	0.00032856
AT3G44190	204.9905456	514.1850101	–1.3267	9.57E–24
AT3G51730	179.6232734	451.4153487	–1.3295	5.80E–21
AT2G06025	40.267466	101.4369003	–1.3329	3.71E–05
AT3G22370	92.1102892	232.1913669	–1.3339	4.47E–11
AT1G32460	107.2157199	270.3887731	–1.3345	7.95E–13
AT1G55520	23.23280913	58.63513273	–1.3356	0.0029235
AT5G46410	52.99214945	133.7613965	–1.3358	1.25E–06
AT3G02875	22.86338284	57.74245042	–1.3366	0.0031735
AT4G03320	28.97944036	73.24693263	–1.3377	0.00064643
AT1G03230	50.44721276	127.6065869	–1.3389	2.24E–06
AT1G63440	65.18321714	165.0992439	–1.3408	4.22E–08
AT1G06620	24.50527748	62.1118954	–1.3418	0.0019597
AT3G08690	31.36018759	79.63665863	–1.3445	0.00031129
AT5G16360	65.3474066	166.461759	–1.349	3.02E–08
AT2G17440	79.63188995	203.1557002	–1.3512	5.73E–10

AT3G07280	30.70342973	78.41509336	–1.3527	0.0003234
AT3G23590	30.70342973	78.46207664	–1.3536	0.00031879
AT3G10340	45.15210255	115.4379176	–1.3543	6.18E–06
AT2G26670	70.51937471	180.415793	–1.3552	5.81E–09
AT1G15110	53.48471784	137.0502261	–1.3575	5.82E–07
AT1G32700	35.99853994	92.41611063	–1.3602	6.63E–05
AT3G28580	24.42318274	62.72267804	–1.3607	0.0015576
AT3G51600	21.26253557	54.64155397	–1.3617	0.0036037
AT1G63090	35.75225575	92.0402444	–1.3642	6.55E–05
AT1G32230	396.8459341	1021.886328	–1.3646	2.78E–49
AT2G30600	76.67647961	197.56469	–1.3655	6.98E–10
AT3G12740	109.5554197	283.0272753	–1.3693	5.24E–14
AT1G21000	67.85129592	175.3415988	–1.3697	6.97E–09
AT4G17490	97.24120995	251.4075282	–1.3704	1.63E–12
AT1G28330	245.299059	634.6031552	–1.3713	5.51E–31
AT4G24060	51.8428232	134.2312293	–1.3725	5.93E–07
AT1G34300	31.77066125	82.26772228	–1.3726	0.00017064
AT2G24100	43.92068157	113.7465195	–1.3729	5.50E–06
AT3G16800	27.62487729	71.55553457	–1.3731	0.00054211
AT1G75180	22.69919337	58.82306584	–1.3737	0.0021202
AT5G13330	21.63196186	56.09803564	–1.3748	0.002805
AT2G46500	139.4379022	362.006168	–1.3764	5.92E–18
AT4G36780	81.10959513	210.7669915	–1.3777	1.14E–10
AT3G06760	39.40547132	102.564499	–1.3801	1.68E–05
AT1G70590	43.46916055	113.2766867	–1.3818	5.03E–06
AT2G45820	416.0971487	1084.327106	–1.3818	1.95E–53
AT1G09060	31.56542442	82.31470556	–1.3828	0.00015083
AT2G42400	21.05729874	54.92345365	–1.3831	0.0029844

AT1G60140	92.93123652	243.2324376	−1.3881	2.17E−12
AT5G61590	26.39345631	69.15938732	−1.3897	0.00059848
AT5G40590	20.85206191	54.64155397	−1.3898	0.002924
AT5G25280	67.89234329	178.0196458	−1.3907	3.09E−09
AT4G01700	26.7628826	70.28698602	−1.393	0.00051161
AT4G17260	20.975204	55.15837005	−1.3949	0.0026677
AT4G18140	22.61709864	59.48083176	−1.395	0.0016564
AT5G47230	91.04305769	239.426792	−1.395	2.63E−12
AT3G18290	69.205859	182.3421075	−1.3977	1.60E−09
AT3G54140	30.29295608	79.91855831	−1.3995	0.0001625
AT5G54540	53.81309677	142.077437	−1.4006	1.44E−07
AT1G14040	82.17682664	216.9687844	−1.4007	2.82E−11
AT1G27290	67.81024856	179.3351776	−1.4031	1.95E−09
AT4G34710	375.8296827	995.0118917	−1.4046	1.77E−50
AT2G23790	49.01055495	130.0967007	−1.4084	4.80E−07
AT1G15380	132.0083289	351.9517462	−1.4147	2.63E−18
AT2G32560	25.16203533	67.18608958	−1.4169	0.00057729
AT5G04760	34.27455057	91.5704116	−1.4177	3.46E−05
AT5G01830	46.87609192	125.4453561	−1.4201	6.67E−07
AT2G46270	49.7904549	133.3855303	−1.4217	2.58E−07
AT5G01100	74.33677975	199.584971	−1.4249	1.02E−10
AT1G28380	67.35872753	181.3554586	−1.4289	7.96E−10
AT4G08770	51.06292325	137.5670422	−1.4298	1.35E−07
AT4G33980	29.02048773	78.36811009	−1.4332	0.00013313
AT2G44410	27.87116148	75.31419692	−1.4341	0.00018939
AT1G76590	30.8676192	83.5832541	−1.4371	6.88E−05
AT2G15490	40.55479756	109.8469073	−1.4375	3.10E−06
AT3G05360	19.08702517	51.72859065	−1.4384	0.0028398

AT1G15230	29.18467719	79.16682584	–1.4397	0.00011284
AT5G05440	80.69912147	218.9420821	–1.4399	6.54E–12
AT1G32530	40.06222917	108.7193086	–1.4403	3.40E–06
AT2G30620	160.618343	436.5686324	–1.4426	2.28E–23
AT5G63620	58.69773332	159.7901333	–1.4448	6.93E–09
AT5G27030	40.71898703	110.9275227	–1.4458	2.40E–06
AT2G29460	17.03465687	46.46646335	–1.4477	0.0049146
AT3G05580	43.96172894	119.9483124	–1.4481	7.81E–07
AT4G00755	18.88178834	51.54065753	–1.4487	0.0026957
AT5G45110	117.436514	321.3656313	–1.4523	1.63E–17
AT3G62650	81.56111615	223.3115271	–1.4531	2.54E–12
AT3G13650	42.40192903	116.2836166	–1.4554	1.08E–06
AT1G72680	32.63265593	89.64409715	–1.4579	2.59E–05
AT5G40690	50.57035486	139.2114569	–1.4609	6.00E–08
AT1G79245	750.4689918	2067.311278	–1.4619	2.91E–112
AT5G27920	50.2830233	138.6476576	–1.4633	6.13E–08
AT1G77220	27.33754573	75.40816348	–1.4638	0.00013565
AT3G55880	27.41964046	75.69006316	–1.4649	0.00012961
AT2G42330	24.91575114	68.92447092	–1.468	0.00028885
AT4G05050	641.7755667	1777.142544	–1.4694	2.24E–97
AT3G24070	33.04312959	91.5704116	–1.4705	1.74E–05
AT5G43830	133.5270815	370.4631583	–1.4722	1.40E–20
AT3G54640	85.13223699	236.8427116	–1.4762	2.21E–13
AT1G08930	265.494363	739.4228516	–1.4777	5.41E–41
AT1G20780	42.11459747	117.3172487	–1.478	6.49E–07
AT5G65300	52.74586525	147.0106813	–1.4788	1.57E–08
AT1G63800	38.29719244	106.7460109	–1.4789	2.39E–06
AT1G17340	33.28941379	92.88594343	–1.4804	1.30E–05

AT5G57655	221.5326341	618.7228068	–1.4818	1.57E–34
AT3G22530	16.66523058	46.60741319	–1.4837	0.0038077
AT2G22850	16.58313585	46.51344663	–1.4879	0.003749
AT2G43400	45.9320025	128.9221187	–1.4889	1.26E–07
AT5G04340	131.2694763	369.0066766	–1.4911	6.12E–21
AT2G18196	18.71759888	52.62127296	–1.4913	0.0017396
AT1G13210	68.95957481	194.2758604	–1.4943	2.74E–11
AT3G10985	133.0755604	375.2554528	–1.4956	2.15E–21
AT4G39090	419.2167485	1184.354508	–1.4983	4.12E–67
AT1G62770	38.87185556	110.0348404	–1.5012	1.11E–06
AT4G30280	153.4350539	435.2061173	–1.5041	5.36E–25
AT4G14040	50.93978115	144.5205675	–1.5044	1.25E–08
AT1G18270	322.9606753	916.5967984	–1.5049	2.35E–52
AT5G54640	30.8676192	87.90571581	–1.5099	1.66E–05
AT5G08500	34.35664531	97.86617105	–1.5102	4.59E–06
AT1G16370	17.65036736	50.31909227	–1.5114	0.0020063
AT2G21500	28.07639831	80.1534747	–1.5134	4.32E–05
AT3G45970	94.0395154	268.4624586	–1.5134	9.55E–16
AT4G05010	21.30358293	60.84334686	–1.514	0.00051457
AT3G23080	15.6800938	44.82204857	–1.5153	0.0038804
AT5G43520	31.15495076	89.08029779	–1.5156	1.33E–05
AT4G36730	55.53708614	158.8034845	–1.5157	1.55E–09
AT4G35750	293.1192403	838.181705	–1.5158	1.73E–48
AT3G10960	24.09480382	68.92447092	–1.5163	0.00017842
AT1G75220	65.75788026	188.2150174	–1.5171	3.17E–11
AT2G40000	692.2227795	1981.707743	–1.5174	1.17E–114
AT3G26460	49.05160232	140.5269888	–1.5185	1.57E–08
AT3G49590	32.71475067	93.96655886	–1.5222	6.44E–06

AT4G14500	46.34247616	133.1975972	–1.5232	3.75E–08
AT1G66480	34.43874004	98.99376975	–1.5233	3.31E–06
AT4G12550	20.60577771	59.24591536	–1.5237	0.00058304
AT1G78850	183.7690574	528.7028434	–1.5246	5.42E–31
AT1G53840	199.8185775	575.1693068	–1.5253	1.01E–33
AT4G11360	39.81594498	114.6861851	–1.5263	4.04E–07
AT1G13260	114.3579616	329.6346885	–1.5273	1.63E–19
AT2G25735	14.61286228	42.19098493	–1.5297	0.0049867
AT3G59350	95.51722058	276.2146998	–1.532	1.63E–16
AT5G63770	41.41679225	119.9952957	–1.5347	1.74E–07
AT2G18690	152.2446803	441.1729938	–1.535	3.36E–26
AT4G00550	19.1691199	55.58121956	–1.5358	0.00084205
AT5G55700	22.00138815	63.80329346	–1.536	0.00028934
AT3G20500	32.30427701	93.82560902	–1.5383	5.27E–06
AT1G57990	123.1010505	358.3414722	–1.5415	1.70E–21
AT2G41250	26.22926685	76.58274547	–1.5458	4.77E–05
AT2G41630	113.7832984	332.406702	–1.5467	4.38E–20
AT1G20440	1653.880469	4834.25057	–1.5474	1.78E–289
AT3G01830	17.03465687	49.94322603	–1.5518	0.0015728
AT5G11530	27.74801939	81.37503997	–1.5522	2.34E–05
AT1G50460	57.5073597	168.6699731	–1.5524	1.72E–10
AT4G15530	115.0557668	337.5278794	–1.5527	1.60E–20
AT4G25170	58.69773332	172.2876857	–1.5534	1.02E–10
AT5G17460	62.72037518	184.2214386	–1.5544	1.96E–11
AT1G56140	93.71113648	275.9797834	–1.5583	5.76E–17
AT4G34890	114.07063	336.0244145	–1.5586	1.47E–20
AT1G53190	24.58737221	72.49520016	–1.56	7.08E–05
AT1G30320	16.33685165	48.20484469	–1.561	0.0018627

AT4G01120	66.98930124	197.6586566	−1.561	2.58E−12
AT1G17380	17.65036736	52.43333984	−1.5708	0.0009755
AT3G43230	48.80531812	145.4602331	−1.5755	2.48E−09
AT1G20450	596.2540379	1777.565394	−1.5759	1.44E−109
AT3G59110	18.18398312	54.26568774	−1.5774	0.00072476
AT5G48970	18.43026731	55.01742021	−1.5778	0.00065551
AT5G01410	101.3459465	302.8072359	−1.5791	5.81E−19
AT2G40970	29.34886666	87.81174925	−1.5811	6.78E−06
AT3G51430	18.34817258	54.92345365	−1.5818	0.00064379
AT2G44790	461.331346	1383.187746	−1.5841	4.96E−86
AT2G36310	80.37074254	241.2121566	−1.5856	2.63E−15
AT5G08660	29.88248242	89.69108043	−1.5857	4.92E−06
AT1G11100	15.3106675	45.99663056	−1.587	0.0021109
AT5G54500	238.3620541	716.6829444	−1.5882	7.70E−45
AT5G13310	13.66877286	41.1103695	−1.5886	0.0040463
AT5G51440	28.32268251	85.1806856	−1.5886	8.90E−06
AT1G16670	26.68078787	80.29442454	−1.5895	1.74E−05
AT1G25220	32.96103486	99.46360255	−1.5934	1.13E−06
AT5G20910	20.31844615	61.31317965	−1.5934	0.00024134
AT1G51760	50.40616539	152.1788421	−1.5941	6.43E−10
AT4G17900	68.38491168	206.6324629	−1.5953	2.63E−13
AT4G39640	32.46846647	98.14807072	−1.5959	1.31E−06
AT5G54810	148.9608911	451.462332	−1.5997	1.08E−28
AT1G54710	30.33400344	91.94627784	−1.5999	2.99E−06
AT1G35670	70.76565891	214.5256539	−1.6	7.36E−14
AT1G19530	241.153275	731.4356941	−1.6008	2.30E−46
AT5G42900	17.28094107	52.43333984	−1.6013	0.00077683
AT3G50910	55.49603877	168.5760066	−1.6029	4.99E−11

AT1G17170	103.2341254	313.8483066	−1.6041	3.80E−20
AT1G08720	28.07639831	85.36861872	−1.6043	7.11E−06
AT1G17060	16.21370955	49.3324434	−1.6053	0.0011622
AT4G20780	35.71120838	108.6723253	−1.6055	2.54E−07
AT5G18400	60.70905425	185.3490373	−1.6103	3.65E−12
AT3G03640	44.08487104	134.7010621	−1.6114	5.41E−09
AT4G27020	34.6029295	106.1352282	−1.6169	3.06E−07
AT1G52720	22.57605128	69.25335388	−1.6171	6.19E−05
AT5G54730	49.09264968	150.7223604	−1.6183	4.63E−10
AT2G35510	43.38706582	133.5264801	−1.6218	5.24E−09
AT5G67340	15.84428326	48.8626106	−1.6248	0.0010808
AT5G17650	17.93769892	55.44026972	−1.6279	0.00041347
AT1G01650	67.522917	208.9816269	−1.6299	6.46E−14
AT4G23100	237.4590121	736.0400555	−1.6321	3.74E−48
AT1G23760	15.92637799	49.37942668	−1.6325	0.00095147
AT4G28260	83.57243709	259.4886523	−1.6346	2.97E−17
AT3G17860	26.43450368	82.26772228	−1.6379	7.38E−06
AT1G62975	38.17405034	118.9616635	−1.6398	3.19E−08
AT3G12350	22.28871971	69.53525355	−1.6414	4.64E−05
AT5G60270	15.1875254	47.40612894	−1.6422	0.0011821
AT4G30780	43.30497108	135.4997779	−1.6457	2.44E−09
AT4G32070	89.68849461	280.8190611	−1.6466	7.66E−19
AT1G15530	15.26962014	47.82897846	−1.6472	0.0010739
AT1G21130	20.72891981	64.97787545	−1.6483	8.50E−05
AT3G51500	18.34817258	57.60150058	−1.6505	0.00025169
AT4G33070	33.69988745	105.8533286	−1.6513	1.86E−07
AT1G29760	34.02826638	107.0279105	−1.6532	1.52E−07
AT4G30790	37.76357668	118.8207137	−1.6537	2.57E−08

AT1G66580	340.9394216	1076.997714	–1.6594	2.73E–72
AT1G13195	16.95256214	53.56093855	–1.6597	0.00042396
AT1G03370	54.10042833	171.0661204	–1.6608	8.00E–12
AT4G35985	49.83150227	157.5819192	–1.661	6.22E–11
AT1G59740	27.25545099	86.30828431	–1.663	2.99E–06
AT2G35800	31.89380335	101.0140508	–1.6632	3.22E–07
AT3G15070	21.46777239	68.03178861	–1.664	4.63E–05
AT2G28400	15.6800938	49.70830963	–1.6646	0.00072232
AT1G02400	57.95888073	183.939539	–1.6661	9.85E–13
AT1G09940	67.2766328	214.1497876	–1.6704	8.18E–15
AT1G23390	23.1507144	73.81073198	–1.6728	1.76E–05
AT1G18570	36.03958731	114.9680848	–1.6736	3.25E–08
AT4G23730	15.47485697	49.52037652	–1.6781	0.00067611
AT5G64300	86.36365797	276.4026329	–1.6783	4.05E–19
AT3G05500	81.47902142	260.8511674	–1.6787	4.39E–18
AT3G48520	83.73662655	268.180559	–1.6793	1.38E–18
AT1G10150	11.37012037	36.45902484	–1.681	0.0047376
AT4G28300	83.61348445	268.3215088	–1.6822	1.21E–18
AT4G02880	37.14786619	119.2435632	–1.6826	1.43E–08
AT1G29640	16.29580428	52.38635656	–1.6847	0.00041722
AT2G41430	618.2964734	1987.815569	–1.6848	1.22E–136
AT5G01720	11.61640457	37.35170714	–1.685	0.0040416
AT1G56220	137.7139128	442.9113752	–1.6853	1.25E–30
AT2G31810	94.36789433	303.9348346	–1.6874	3.71E–21
AT1G34260	26.43450368	85.1806856	–1.6881	2.59E–06
AT1G25560	47.45075504	152.9775578	–1.6888	6.64E–11
AT5G06560	34.93130843	112.7598706	–1.6907	3.42E–08
AT1G36370	77.94894795	251.7364112	–1.6913	1.13E–17

AT3G53180	87.80031578	283.8729743	−1.6929	6.71E−20
AT4G29950	88.33393154	285.6113556	−1.693	5.10E−20
AT5G64410	38.05090824	123.2841252	−1.696	5.99E−09
AT5G28770	36.49110833	118.2569143	−1.6963	1.31E−08
AT3G20660	28.61001407	92.79197687	−1.6975	6.99E−07
AT4G09150	74.9935376	243.5143373	−1.6992	3.07E−17
AT5G27520	30.00562451	97.53728809	−1.7007	3.18E−07
AT5G06570	29.84143505	97.34935497	−1.7059	3.05E−07
ATMG00020	18.43026731	60.13859767	−1.7062	0.00010473
AT2G41190	13.13515711	42.89573412	−1.7074	0.0015282
AT3G07310	18.55340941	60.65541374	−1.709	9.42E−05
AT4G24390	12.84782554	42.09701837	−1.7122	0.0016847
AT5G47040	80.53493201	263.9520638	−1.7126	7.26E−19
AT4G34310	25.61355636	84.00610362	−1.7136	2.26E−06
AT3G28740	20.81101454	68.40765485	−1.7168	2.58E−05
AT2G46260	71.58660623	235.3392467	−1.717	5.99E−17
AT3G47950	13.21725184	43.45953347	−1.7173	0.0013134
AT1G19310	15.14647804	49.84925947	−1.7186	0.00048075
AT1G14860	30.21086134	99.60455239	−1.7211	1.70E−07
AT1G08570	62.43304362	205.9277137	−1.7218	6.02E−15
AT5G18490	26.18821948	86.59018399	−1.7253	1.30E−06
AT2G02180	66.04521182	218.754149	−1.7278	6.09E−16
AT4G17090	28.97944036	95.98683987	−1.7278	2.76E−07
AT3G09370	11.00069408	36.45902484	−1.7287	0.0036749
AT5G54170	28.56896671	94.76527461	−1.7299	3.26E−07
AT4G34230	120.7203033	400.9553066	−1.7318	5.74E−29
AT3G13110	67.31768017	224.7210255	−1.7391	1.59E−16
AT5G47240	11.00069408	36.74092451	−1.7398	0.0033219

AT5G47370	28.28163514	94.57734149	–1.7416	2.85E–07
AT3G56040	12.72468345	42.56685116	–1.7421	0.0012941
AT1G12950	32.46846647	108.625342	–1.7422	2.86E–08
AT4G14370	21.71405659	72.68313327	–1.743	9.86E–06
AT1G11185	13.70982023	46.04361384	–1.7478	0.00071564
AT1G26270	167.9247741	564.8329853	–1.75	2.40E–41
AT4G38810	35.42387682	119.1495966	–1.75	4.46E–09
AT3G47160	29.10258246	98.14807072	–1.7538	1.34E–07
AT1G30420	39.03604502	131.6941322	–1.7543	5.18E–10
AT3G20340	30.621335	103.4571813	–1.7564	5.36E–08
AT5G01750	104.7939253	354.7237597	–1.7591	2.70E–26
AT1G30820	163.8610849	554.8255468	–1.7596	5.70E–41
AT2G24500	71.29927467	241.7289727	–1.7614	4.33E–18
AT3G14590	42.52507113	144.3326344	–1.763	5.24E–11
AT1G76180	909.4454401	3086.895425	–1.7631	3.81E–228
AT2G27830	38.50242927	130.7544666	–1.7638	5.03E–10
AT3G14780	13.70982023	46.60741319	–1.7653	0.00058304
AT5G02160	11.00069408	37.39869042	–1.7654	0.0026195
AT5G59690	76.9227638	261.5559166	–1.7656	1.32E–19
AT5G11520	125.4817977	426.749127	–1.7659	9.02E–32
AT1G55510	48.51798656	165.1462272	–1.7672	1.45E–12
AT5G43620	26.14717211	89.26823091	–1.7715	4.55E–07
AT2G38400	146.9495701	504.3185214	–1.779	6.36E–38
AT4G11850	90.38629983	310.8413767	–1.782	1.48E–23
AT2G35710	33.28941379	114.498252	–1.7822	5.50E–09
AT1G15430	24.99784587	86.12035119	–1.7846	6.55E–07
AT3G07350	11.12383617	38.33835601	–1.7851	0.0020167
AT4G37790	38.13300297	131.6001657	–1.787	2.77E–10

AT5G13200	44.20801313	152.7896247	–1.7892	6.96E–12
AT1G80840	134.265934	464.1948007	–1.7896	2.95E–35
AT3G49570	11.00069408	38.05645634	–1.7905	0.0020514
AT2G33150	266.6847366	925.288705	–1.7948	2.43E–70
AT2G02710	62.59723308	217.2506841	–1.7952	8.70E–17
AT2G36320	292.8729561	1016.859117	–1.7958	2.36E–77
AT4G30390	19.1691199	66.57530695	–1.7962	1.58E–05
AT3G25495	12.35525715	42.9427174	–1.7973	0.00086402
AT5G52660	10.87755198	37.86852322	–1.7996	0.0020133
AT1G17420	9.399846804	32.74734576	–1.8007	0.0047016
AT1G62320	10.95964671	38.19740617	–1.8013	0.0018865
AT3G45300	183.5227732	639.9592491	–1.802	5.22E–49
AT4G38060	43.71544474	152.507725	–1.8027	5.38E–12
AT1G66500	51.39130218	179.3351776	–1.8031	5.07E–14
AT4G17500	90.91991559	317.6539522	–1.8048	1.53E–24
AT5G13210	31.64751915	110.6456231	–1.8058	7.12E–09
AT4G36430	57.13793341	199.8668707	–1.8065	1.25E–15
AT2G23450	56.97374395	199.3500546	–1.8069	1.35E–15
AT1G78280	60.66800688	212.364423	–1.8075	1.37E–16
AT5G02810	29.96457715	105.1015961	–1.8105	1.74E–08
AT1G19400	41.70412381	146.6817984	–1.8144	1.14E–11
AT4G21830	32.71475067	115.2030012	–1.8162	2.74E–09
AT4G21850	81.72530562	287.8665531	–1.8165	1.70E–22
AT2G30140	17.32198843	61.12524654	–1.8192	3.24E–05
AT1G08800	52.49958106	185.4430039	–1.8206	1.06E–14
AT5G21170	77.0459059	272.550004	–1.8227	2.02E–21
AT5G66650	67.6460591	239.3328254	–1.8229	7.23E–19
AT2G38250	17.28094107	61.21921309	–1.8248	3.04E–05

AT2G15695	31.93485071	113.2297034	−1.826	3.27E−09
AT4G13830	17.4861779	62.1118954	−1.8287	2.51E−05
AT4G34410	63.2950383	224.9559419	−1.8295	7.25E−18
AT2G29440	19.74378302	70.28698602	−1.8319	5.78E−06
AT4G08555	23.72537752	84.52291969	−1.8329	4.66E−07
AT2G27150	27.66592465	98.61790352	−1.8337	3.82E−08
AT2G26660	19.94901985	71.36760145	−1.839	4.45E−06
AT2G26530	82.66939504	295.8537106	−1.8395	1.49E−23
AT1G68440	234.7498859	841.7524342	−1.8423	1.23E−66
AT1G09070	423.1572956	1519.533223	−1.8444	2.11E−120
AT1G77000	18.47131468	66.34039055	−1.8446	1.02E−05
AT2G30720	10.09765203	36.27109172	−1.8448	0.0020758
AT2G34500	19.45645146	70.14603619	−1.8501	4.91E−06
AT1G67070	12.23211505	44.21126594	−1.8537	0.00048699
AT5G03030	18.47131468	66.76324007	−1.8538	8.65E−06
AT1G33590	59.14925434	214.1028044	−1.8559	2.14E−17
AT5G47860	15.59799906	56.52088515	−1.8574	5.24E−05
AT5G13740	45.39838675	164.6294111	−1.8585	1.65E−13
AT3G29160	50.48826013	183.4227229	−1.8612	4.97E−15
AT3G54130	16.95256214	61.59507933	−1.8613	2.04E−05
AT4G31780	22.49395654	81.84487276	−1.8634	5.17E−07
AT5G16110	212.4611662	773.0628797	−1.8634	2.76E−62
AT3G30775	796.6883258	2899.244207	−1.8636	1.85E−233
AT5G21940	172.1937002	627.50868	−1.8656	1.13E−50
AT4G14680	19.94901985	72.73011655	−1.8662	2.62E−06
AT3G13450	44.90581836	163.8306954	−1.8672	1.54E−13
AT5G62540	36.61425043	134.2782126	−1.8747	2.99E−11
AT3G45730	42.1966922	154.9038723	−1.8762	6.48E−13

AT4G11280	243.3698328	894.6086236	–1.8781	6.32E–73
AT5G11920	13.70982023	50.50702538	–1.8813	0.00012894
AT5G60200	19.0459778	70.24000274	–1.8828	3.46E–06
AT2G40420	22.20662498	81.9858226	–1.8844	3.92E–07
AT3G59930	73.1874535	270.2478233	–1.8846	2.42E–22
AT5G35110	9.194609974	33.96891103	–1.8854	0.0025354
AT3G62770	37.96881351	140.2920724	–1.8855	7.86E–12
AT4G03430	99.29357825	368.067011	–1.8902	1.89E–30
AT5G28050	240.9890855	894.4676737	–1.8921	9.81E–74
AT2G29670	9.687178366	36.03617532	–1.8953	0.0016564
AT1G03740	57.91783336	215.559286	–1.896	4.50E–18
AT1G70700	11.86268876	44.21126594	–1.898	0.00036656
AT2G34600	8.455757387	31.5257805	–1.8985	0.0036772
AT1G72360	27.1323089	101.389917	–1.9018	8.30E–09
AT1G53440	32.6737033	122.579376	–1.9075	1.41E–10
AT5G13700	12.92992028	48.53372765	–1.9083	0.00015268
AT2G43140	21.50881976	80.76425734	–1.9088	3.66E–07
AT3G53650	10.09765203	37.9155065	–1.9088	0.0010827
AT4G20830	439.3299578	1651.368305	–1.9103	5.34E–138
AT1G09950	13.13515711	49.42640996	–1.9118	0.00012592
AT5G66850	31.68856652	119.384513	–1.9136	2.33E–10
AT5G20010	214.2262029	810.1796705	–1.9191	3.09E–68
AT1G55920	75.85553229	286.9268875	–1.9194	2.36E–24
AT1G58200	21.26253557	80.48235766	–1.9204	3.37E–07
AT5G17490	8.291567923	31.38483066	–1.9204	0.0034196
AT5G58650	13.95610442	52.95015591	–1.9237	5.92E–05
AT1G22830	9.03042051	34.29779398	–1.9253	0.0019629
AT5G22290	17.85560419	67.84385549	–1.9258	3.48E–06

AT1G72330	13.17620447	50.13115915	−1.9278	9.78E−05
AT4G33030	16.37789902	62.3468118	−1.9286	9.57E−06
AT4G32190	18.47131468	70.42793586	−1.9309	2.04E−06
AT3G23250	76.01972175	289.9338173	−1.9313	7.84E−25
AT3G62150	38.29719244	146.1649823	−1.9323	9.60E−13
AT3G14260	11.28802564	43.17763379	−1.9355	0.00034956
AT5G64310	134.8405972	516.1113245	−1.9364	3.93E−44
AT3G06420	34.15140848	130.8484332	−1.9379	1.64E−11
AT5G21990	63.74655933	244.3600363	−1.9386	4.26E−21
AT5G16650	15.14647804	58.07133337	−1.9388	1.98E−05
AT3G54150	15.80323589	60.60843046	−1.9393	1.21E−05
AT3G06780	22.61709864	86.91906694	−1.9423	7.51E−08
AT5G07440	816.226872	3137.8253	−1.9427	4.40E−269
AT3G13080	65.3474066	251.6424446	−1.9452	8.01E−22
AT1G77680	25.81879319	99.46360255	−1.9457	6.24E−09
AT3G13520	134.9637393	521.0915521	−1.949	5.58E−45
AT1G28480	19.292262	74.56246445	−1.9504	7.41E−07
AT2G16060	16.21370955	62.72267804	−1.9518	7.17E−06
AT2G19810	13.70982023	53.13808903	−1.9545	4.50E−05
AT5G27610	9.810320463	38.05645634	−1.9558	0.00081694
AT5G46710	11.49326247	44.63411546	−1.9574	0.0002305
AT5G46910	33.24836642	129.1570351	−1.9578	1.55E−11
AT2G20562	8.045283727	31.2908641	−1.9595	0.0029221
AT2G25460	105.1223042	409.4592802	−1.9617	9.55E−36
AT3G29240	77.98999532	304.3107009	−1.9642	1.08E−26
AT3G14770	55.9475598	218.5192326	−1.9656	2.68E−19
AT1G05060	11.78059403	46.1375804	−1.9695	0.00015729
AT1G32170	113.0854932	443.4751745	−1.9714	5.57E−39

AT5G55970	19.70273566	77.28749466	–1.9718	3.42E–07
AT4G29670	55.20870721	216.6399014	–1.9723	3.14E–19
AT4G32870	31.89380335	125.5863059	–1.9773	2.16E–11
AT3G57750	9.276704706	36.55299139	–1.9783	0.00096468
AT4G13110	8.045283727	31.76069689	–1.981	0.002429
AT3G03990	108.3650461	429.7090736	–1.9875	3.15E–38
AT3G14075	20.76996717	82.54962195	–1.9908	9.67E–08
AT1G73080	69.28795373	275.415984	–1.9909	1.11E–24
AT2G16900	64.19808035	255.3071404	–1.9916	6.46E–23
AT5G42940	58.86192278	234.3056145	–1.993	4.47E–21
AT5G43190	25.53146162	101.8127665	–1.9956	1.89E–09
AT4G21680	16.74732531	66.90418991	–1.9982	2.05E–06
AT5G13820	22.16557762	88.610465	–1.9992	2.57E–08
AT4G37610	79.26246366	316.9022197	–1.9993	1.61E–28
AT5G38710	14.12029389	56.61485171	–2.0034	1.53E–05
AT1G26250	74.33677975	298.0619247	–2.0035	6.48E–27
AT5G54080	54.71613882	219.599848	–2.0048	5.92E–20
AT3G46930	27.37859309	109.9408739	–2.0056	3.11E–10
AT5G16970	34.80816633	140.1981058	–2.01	5.95E–13
AT3G07565	19.1691199	77.24051138	–2.0106	2.22E–07
AT4G15260	13.46353603	54.31267102	–2.0122	2.26E–05
AT1G64660	283.6783461	1146.767884	–2.0152	9.14E–104
AT1G21400	107.4209567	435.8169	–2.0204	1.06E–39
AT5G51130	10.01555729	40.6405367	–2.0207	0.00034109
AT4G04610	128.9297765	523.8165823	–2.0225	9.55E–48
AT4G22530	10.67231515	43.45953347	–2.0258	0.00018637
AT4G34350	129.8328185	529.3606093	–2.0276	2.03E–48
AT2G30870	573.6779866	2339.297483	–2.0278	1.50E–213

AT5G47220	22.98652494	93.82560902	–2.0292	5.84E–09
AT1G78895	19.99006722	81.84487276	–2.0336	6.62E–08
AT5G16010	45.15210255	184.9261878	–2.0341	3.11E–17
AT3G08860	40.18537127	165.0992439	–2.0386	1.76E–15
AT5G19230	73.1874535	301.4917041	–2.0425	5.38E–28
AT2G38870	25.12098796	103.5511479	–2.0434	6.39E–10
AT5G66700	8.866231046	36.55299139	–2.0436	0.00068534
AT5G18630	47.45075504	196.8599408	–2.0527	1.51E–18
AT2G15890	46.87609192	194.7456932	–2.0547	2.24E–18
AT4G21990	232.6154229	966.7749408	–2.0552	4.85E–90
AT3G48390	11.61640457	48.29881125	–2.0558	5.58E–05
AT1G03905	24.66946694	102.6584655	–2.0571	6.26E–10
AT5G02230	52.86900735	220.9623631	–2.0633	6.55E–21
AT1G32200	13.91505706	58.16529993	–2.0635	6.68E–06
AT1G25230	24.71051431	103.5041646	–2.0665	4.54E–10
AT2G17280	17.93769892	75.22023036	–2.0681	1.77E–07
AT1G29400	94.24475223	395.5052462	–2.0692	2.55E–37
AT3G23920	95.51722058	401.0492732	–2.0699	7.39E–38
AT1G70290	94.12161014	396.1630121	–2.0735	1.72E–37
AT5G35735	152.6962014	642.8252291	–2.0738	1.10E–60
AT5G52750	9.728225732	40.96941966	–2.0743	0.00023237
AT5G22220	6.895957481	29.12963325	–2.0787	0.0026834
AT5G07460	18.06084102	76.62972875	–2.085	1.09E–07
AT5G10695	39.98013444	169.985505	–2.0881	1.81E–16
AT2G05520	17.81455682	75.78402972	–2.0888	1.26E–07
AT1G53920	11.16488354	47.5000955	–2.089	5.24E–05
AT2G19800	141.2850336	603.5941908	–2.095	8.00E–58
AT3G11170	22.45290918	96.55063922	–2.1044	1.15E–09

AT4G17615	32.96103486	142.1714035	−2.1088	4.95E−14
AT1G19380	54.59299672	235.8090794	−2.1108	5.09E−23
AT3G01970	20.72891981	89.59711387	−2.1118	4.74E−09
AT2G20670	83.98291075	363.697566	−2.1146	2.06E−35
AT3G02550	19.66168829	85.22766888	−2.1159	1.16E−08
AT5G57660	12.43735188	53.98378806	−2.1178	1.05E−05
AT1G58180	48.27170236	209.9212925	−2.1206	1.15E−20
AT3G17790	26.80392997	116.9413825	−2.1253	9.34E−12
AT1G23870	123.0189558	538.5223488	−2.1301	8.01E−53
AT5G47450	28.56896671	125.1164731	−2.1308	1.39E−12
AT1G09932	13.99715179	61.40714621	−2.1333	1.83E−06
AT2G35390	19.00493044	83.72420394	−2.1393	1.21E−08
AT2G25900	65.79892763	289.8868341	−2.1394	9.53E−29
AT5G40010	7.51166797	33.123212	−2.1406	0.00086603
AT1G54100	330.3902486	1457.468311	−2.1412	8.91E−144
AT3G12580	306.2954448	1355.655545	−2.146	3.54E−134
AT5G58787	15.72114116	69.72318667	−2.1489	2.48E−07
AT3G55840	12.02687823	53.37300543	−2.1498	9.33E−06
AT2G15480	51.92491793	231.2047181	−2.1547	3.15E−23
AT4G23060	7.265383774	32.41846281	−2.1577	0.00093249
AT2G38290	18.34817258	81.89185604	−2.1581	1.45E−08
AT4G36500	85.21433172	381.2693126	−2.1616	2.91E−38
AT4G12400	37.84567141	169.4686889	−2.1628	3.10E−17
AT4G21410	57.30212287	256.7636221	−2.1638	6.64E−26
AT1G76070	29.38991402	131.9290486	−2.1664	1.52E−13
AT5G43450	6.854910115	30.77404803	−2.1665	0.0012891
AT4G26260	18.84074097	84.80481937	−2.1703	6.46E−09
AT1G11960	35.75225575	161.0116986	−2.1711	1.76E−16

AT5G05850	7.30643114	33.02924544	−2.1765	0.00074641
AT5G15120	6.198152259	28.0960011	−2.1805	0.002218
AT1G69890	82.50520557	374.78562	−2.1835	3.81E−38
AT3G19210	7.183289042	32.6533792	−2.1845	0.00078246
AT5G16120	46.34247616	210.9549246	−2.1865	1.19E−21
AT1G15030	17.73246209	80.81124062	−2.1882	1.30E−08
AT2G43510	6.321294357	28.84773357	−2.1902	0.0018028
AT5G65110	114.3579616	522.2191508	−2.1911	2.77E−53
AT3G03150	33.00208223	150.7693437	−2.1917	1.16E−15
AT4G18510	14.24343599	65.16580857	−2.1938	4.46E−07
AT4G23550	14.28448335	65.58865808	−2.199	3.87E−07
AT3G51450	8.578899485	39.41897144	−2.2	0.00015595
AT3G43430	36.5321557	167.8712574	−2.2001	1.78E−17
AT1G20630	38.99499766	179.241211	−2.2005	1.26E−18
AT5G15870	33.82302955	155.5146549	−2.201	3.12E−16
AT3G22890	254.16529	1169.695725	−2.2023	4.93E−120
AT4G19870	5.746631234	26.54555288	−2.2077	0.0028165
AT3G02840	24.62841957	114.4512687	−2.2163	3.53E−12
AT3G60140	133.0755604	618.8167733	−2.2173	3.69E−64
AT1G05340	71.87393779	334.4739662	−2.2184	7.91E−35
AT1G13990	22.04243552	102.7994154	−2.2215	5.06E−11
AT2G31945	9.112515242	42.51986788	−2.2222	6.68E−05
AT5G04040	58.04097546	271.4224053	−2.2254	1.94E−28
AT4G18250	8.784136315	41.1103695	−2.2265	9.03E−05
AT5G60800	10.34393622	48.48674437	−2.2288	1.60E−05
AT4G38940	17.56827263	82.59660523	−2.2331	5.07E−09
AT2G43500	5.17196811	24.38432202	−2.2372	0.0041391
AT4G35110	9.974509927	47.12422927	−2.2402	2.03E−05

AT5G45350	54.59299672	258.0321706	–2.2408	2.67E–27
AT2G47550	28.77420354	136.3924602	–2.2449	1.13E–14
AT4G18340	71.05299047	336.8701135	–2.2452	1.21E–35
AT4G39780	12.84782554	60.93731342	–2.2458	7.48E–07
AT1G14870	192.7994779	915.6571328	–2.2477	1.17E–96
AT4G39230	15.4338096	73.48184902	–2.2513	3.59E–08
AT3G57520	643.6637456	3066.927531	–2.2524	0
AT5G51260	5.58244177	26.63951944	–2.2546	0.0023221
AT2G04050	10.59022042	50.74194178	–2.2604	7.45E–06
AT3G10020	95.27093638	457.1003256	–2.2624	9.17E–49
AT2G40880	8.332615289	40.02975407	–2.2642	9.36E–05
AT2G45180	7.7169048	37.06980747	–2.2642	0.00019066
AT4G33666	9.276704706	44.58713218	–2.2649	3.15E–05
AT3G04320	13.54563077	65.11882529	–2.2652	2.32E–07
AT3G16330	8.743088949	42.42590132	–2.2787	4.85E–05
AT5G19120	94.08056277	457.9460246	–2.2832	1.88E–49
AT4G30490	22.12453025	108.5783588	–2.295	4.06E–12
AT1G17300	10.67231515	52.38635656	–2.2953	3.90E–06
AT1G19540	5.910820698	29.03566669	–2.2964	0.0011084
AT1G33600	7.7169048	37.96248978	–2.2985	0.00012757
AT5G14730	27.74801939	136.6273766	–2.2998	3.64E–15
AT1G03090	147.5652806	727.5830652	–2.3018	2.51E–79
AT4G34030	98.0211099	486.089009	–2.3101	2.70E–53
AT1G28190	11.8216414	58.72909928	–2.3126	7.20E–07
AT4G36790	19.33330937	96.08080643	–2.3132	6.83E–11
AT5G20250	882.3131312	4399.702219	–2.318	0
AT3G13100	8.866231046	44.3052325	–2.3211	2.37E–05
AT1G04310	18.22503048	91.14756209	–2.3223	2.08E–10

AT3G05390	7.675857434	38.62025569	–2.331	9.08E–05
AT3G43190	12.88887291	64.88390889	–2.3317	1.33E–07
AT1G76650	89.81163671	452.4019976	–2.3326	2.91E–50
AT3G01650	30.9907613	156.2663874	–2.3341	1.29E–17
AT5G02970	29.43096139	148.7960459	–2.3379	7.80E–17
AT4G38470	193.825662	981.95054	–2.3409	1.94E–109
AT3G47040	4.392068157	22.36404101	–2.3482	0.0046725
AT1G10070	119.6530718	610.6416827	–2.3515	1.64E–68
AT4G19720	9.44089417	48.25182797	–2.3536	7.12E–06
AT3G15450	624.5767204	3200.078145	–2.3572	0
AT5G24490	30.90866656	158.4276182	–2.3577	4.37E–18
AT2G16660	43.75649211	224.6740422	–2.3603	1.64E–25
AT1G27760	135.1689761	696.1512513	–2.3646	1.16E–78
AT4G15610	296.3209348	1527.80228	–2.3662	1.31E–172
AT4G05320	1192.138649	6147.527197	–2.3665	0
AT5G54940	268.0803471	1383.516629	–2.3676	1.89E–156
AT4G15760	44.70058153	232.1443837	–2.3767	1.41E–26
AT5G63130	10.13869939	52.85618936	–2.3822	1.83E–06
AT3G09940	62.51513835	327.1915579	–2.3879	1.43E–37
AT3G19970	19.4154041	101.7657832	–2.39	5.47E–12
AT2G42270	57.58945444	302.2904198	–2.3921	8.07E–35
AT4G12120	13.87400969	73.05899951	–2.3967	8.66E–09
AT1G80380	309.3329498	1629.66203	–2.3973	2.79E–187
AT1G07040	34.56188213	182.4830573	–2.4005	3.08E–21
AT2G03760	22.61709864	119.4314963	–2.4007	4.63E–14
AT5G42380	73.26954823	388.2698212	–2.4058	5.89E–45
AT2G36950	67.522917	363.2277333	–2.4274	1.50E–42
AT5G19110	18.14293575	97.86617105	–2.4314	8.49E–12

AT5G05730	32.7968454	176.9860136	–2.432	5.89E–21
AT1G13480	9.522988902	51.39970769	–2.4323	1.85E–06
AT5G38200	13.58667813	73.48184902	–2.4352	5.25E–09
AT4G15130	13.34039394	72.21330048	–2.4365	7.19E–09
AT3G14050	40.96527122	221.9959953	–2.4381	2.83E–26
AT5G53050	7.10119431	38.66723897	–2.445	4.85E–05
AT5G54490	16.41894638	89.78504699	–2.4511	5.67E–11
AT1G70670	36.81948726	201.5112855	–2.4523	4.59E–24
AT4G32480	71.70974832	394.5185974	–2.4599	5.26E–47
AT4G25110	3.899499766	21.4713587	–2.4611	0.004166
AT2G41880	12.51944662	69.06542076	–2.4638	1.28E–08
AT4G05120	7.51166797	41.48623573	–2.4654	2.05E–05
AT5G63790	142.3933125	788.6613285	–2.4695	3.12E–94
AT1G11260	174.5744474	966.9628739	–2.4696	1.68E–115
AT3G02555	6.157104893	34.2508107	–2.4758	0.00013459
AT1G60730	45.27524465	252.1122774	–2.4773	2.05E–30
AT4G18950	121.2128717	677.7338057	–2.4832	1.45E–81
AT2G02230	27.87116148	155.8435379	–2.4833	5.44E–19
AT4G39660	173.8766422	973.4935497	–2.4851	3.33E–117
AT5G06730	6.649673285	37.25774059	–2.4862	5.69E–05
AT1G72900	31.23704549	175.1066824	–2.4869	2.53E–21
AT4G20070	29.59515085	166.8846085	–2.4954	1.95E–20
AT3G26910	26.96811943	152.2728086	–2.4973	1.06E–18
AT5G11070	41.25260279	233.3189656	–2.4997	1.74E–28
AT4G35770	130.1201501	740.8793333	–2.5094	2.37E–90
AT4G36670	15.5569517	88.89236468	–2.5145	3.30E–11
AT1G28760	7.224336408	41.3452859	–2.5168	1.60E–05
AT1G56660	657.2914711	3779.898797	–2.5237	0

AT3G53620	53.36157574	307.1766809	–2.5252	7.02E–38
AT5G40780	36.49110833	210.0622423	–2.5252	5.32E–26
AT5G20830	53.11529155	306.8477979	–2.5303	6.21E–38
AT1G68290	3.899499766	22.55197413	–2.5319	0.0025495
AT3G60450	56.52222292	328.4601065	–2.5388	9.59E–41
AT1G65390	4.351020791	25.37097089	–2.5438	0.0011297
AT5G17850	18.59445678	108.5783588	–2.5458	8.77E–14
AT4G37030	4.966731281	29.03566669	–2.5475	0.00041029
AT1G67810	16.41894638	96.55063922	–2.5559	2.28E–12
AT1G08310	3.44797874	20.29677671	–2.5574	0.004364
AT4G36040	135.7025918	801.0649142	–2.5615	3.37E–100
AT5G27760	55.41394404	328.7420062	–2.5686	2.33E–41
AT1G06570	64.23912772	381.9270785	–2.5718	4.16E–48
AT1G68390	3.981594498	23.67957283	–2.5722	0.0016533
AT1G11210	21.63196186	128.6872024	–2.5726	1.83E–16
AT2G32140	9.071467876	54.03077134	–2.5744	3.22E–07
AT5G04250	10.63126778	63.33346067	–2.5747	2.30E–08
AT1G68500	7.429573238	44.35221578	–2.5777	4.84E–06
AT5G01810	138.0833391	829.9596311	–2.5875	4.36E–105
AT4G24960	48.31274973	290.4506334	–2.5878	6.32E–37
AT5G48180	165.5850743	995.4817245	–2.5878	4.71E–126
AT5G50450	4.392068157	26.45158632	–2.5904	0.00071606
AT5G49700	9.23565734	55.86311924	–2.5966	1.62E–07
AT1G02660	32.9199875	199.5379877	–2.5996	1.31E–25
AT5G65280	17.03465687	103.2692482	–2.5999	1.82E–13
AT2G28210	3.612168204	21.98817477	–2.6058	0.0023975
AT1G01470	167.5143005	1021.510461	–2.6083	1.39E–130
AT3G59480	5.869773332	35.8482422	–2.6105	4.56E–05

AT3G47420	33.82302955	206.9143626	–2.613	1.07E–26
AT1G71695	14.36657808	87.99968237	–2.6148	1.26E–11
AT4G36880	3.242741911	19.8739272	–2.6156	0.0042011
AT1G08630	37.96881351	233.2719824	–2.6191	3.72E–30
AT1G68410	79.05722683	486.089009	–2.6203	1.47E–62
AT1G76680	180.2800313	1112.141207	–2.625	2.98E–143
AT5G20790	6.075010161	37.49265698	–2.6256	2.65E–05
AT2G34790	16.17266219	100.6851678	–2.6382	2.29E–13
AT5G62520	40.30851337	250.9846787	–2.6384	1.04E–32
AT5G02320	7.10119431	44.25824922	–2.6398	3.46E–06
AT2G22880	29.0615351	181.4964084	–2.6428	8.75E–24
AT5G23360	10.91859934	68.40765485	–2.6474	2.78E–09
AT5G22920	60.87324371	382.021045	–2.6498	7.22E–50
AT1G67850	7.798999532	49.14451028	–2.6557	7.50E–07
AT1G76410	19.12807254	120.7470281	–2.6582	4.37E–16
AT1G74360	12.68363608	80.24744126	–2.6615	7.24E–11
AT4G22212	174.2871158	1103.120418	–2.6621	1.75E–144
AT1G23550	4.843589183	30.72706475	–2.6654	0.00015818
AT3G26740	11.5753572	73.57581558	–2.6682	4.99E–10
AT5G66050	39.69280288	253.051943	–2.6725	1.76E–33
AT5G54710	3.119599813	20.01487704	–2.6816	0.0034028
AT2G23120	356.0448523	2288.038725	–2.684	7.69E–303
AT4G03960	8.619946851	55.39328645	–2.684	9.75E–08
AT5G23510	20.23635142	130.2846339	–2.6866	1.57E–17
AT5G25250	11.41116774	73.48184902	–2.6869	4.27E–10
AT3G15630	55.16765984	356.2742079	–2.6911	2.09E–47
AT2G29500	7.265383774	47.03026271	–2.6945	1.10E–06
AT1G14640	3.530073472	22.97482364	–2.7023	0.0013542

AT3G03110	19.78483039	129.0160853	–2.7051	1.67E–17
AT2G32020	22.78128811	149.3598453	–2.7129	2.98E–20
AT5G51830	42.4429764	282.1815762	–2.733	2.79E–38
AT5G02580	6.033962796	40.12372063	–2.7333	6.99E–06
AT5G50720	7.881094264	52.43333984	–2.734	1.67E–07
AT1G67480	218.5361763	1457.750211	–2.7378	2.44E–197
AT3G49160	9.112515242	60.89033014	–2.7403	1.20E–08
AT4G02520	166.0776427	1123.088311	–2.7575	2.42E–153
AT1G67980	14.07924652	95.28209068	–2.7586	2.46E–13
AT5G57500	2.832268251	19.21616129	–2.7623	0.0035204
AT2G35980	16.62418321	112.994787	–2.7649	8.96E–16
AT1G80820	35.09549789	238.6280762	–2.7654	7.21E–33
AT3G16150	61.07848054	415.8020229	–2.7672	4.26E–57
AT5G52760	3.65321557	24.94812138	–2.7717	0.00061059
AT2G32120	5.623489136	38.47930585	–2.7745	9.39E–06
AT4G28350	11.20593091	77.14654482	–2.7833	5.34E–11
AT5G28610	26.55764577	183.0938399	–2.7854	1.68E–25
AT2G20500	4.145783962	28.61281717	–2.7869	0.00018839
AT3G19930	88.7444052	612.6619637	–2.7874	9.17E–85
AT1G61360	24.34108801	168.1061738	–2.7879	1.83E–23
AT2G45170	50.11883383	346.2197861	–2.7883	5.24E–48
AT5G64810	6.075010161	42.28495148	–2.7992	2.56E–06
AT3G17770	51.10397061	356.6030909	–2.8028	1.03E–49
AT5G56550	10.67231515	74.46849789	–2.8028	1.02E–10
AT3G51890	20.44158825	142.8291695	–2.8047	3.96E–20
AT3G06850	167.0627794	1168.427176	–2.8061	9.90E–163
AT5G63160	61.11952791	427.5478428	–2.8064	1.17E–59
AT4G29190	10.79545725	75.97196283	–2.815	5.65E–11

AT4G28240	14.98228857	106.4171279	–2.8284	2.94E–15
AT1G80670	25.61355636	183.7046226	–2.8424	3.63E–26
AT5G38940	11.24697827	80.90520717	–2.8467	8.50E–12
AT1G21790	7.388525872	53.23205559	–2.8489	5.99E–08
AT1G76700	31.40123496	226.2714737	–2.8492	3.00E–32
AT5G65207	34.23350321	248.1656819	–2.8578	1.87E–35
AT2G18193	33.98721901	246.8971334	–2.8608	2.56E–35
AT3G60930	4.843589183	35.33142613	–2.8668	1.65E–05
AT5G61560	4.022641864	29.36454964	–2.8679	0.00011031
AT3G05950	3.201694545	23.44465643	–2.8724	0.00071574
AT4G01010	3.283789276	24.05543907	–2.8729	0.00059166
AT1G47510	3.612168204	26.82745255	–2.8928	0.00023033
AT5G07010	31.27809286	232.8021496	–2.8959	9.00E–34
AT2G41730	6.895957481	51.49367425	–2.9006	7.49E–08
AT4G13300	4.392068157	32.8882956	–2.9046	3.08E–05
AT1G07150	15.51590433	116.2366333	–2.9052	4.05E–17
AT2G34930	12.10897296	91.71136144	–2.921	1.13E–13
AT3G61390	3.406931374	25.98175352	–2.931	0.00026761
AT5G18670	65.63473816	500.888742	–2.932	2.39E–73
AT4G02890	155.4463749	1186.421772	–2.9321	1.70E–173
AT4G37390	5.541394404	42.33193476	–2.9334	1.24E–06
AT2G45760	2.996457715	22.97482364	–2.9387	0.00069068
AT1G14540	10.50812568	81.14012357	–2.9489	2.80E–12
AT4G01026	4.104736596	32.04259657	–2.9646	3.20E–05
AT1G74010	7.881094264	62.01792884	–2.9762	1.36E–09
AT5G16370	88.21078944	698.3124821	–2.9848	3.52E–104
AT1G30755	20.31844615	160.9647153	–2.9859	3.15E–24
AT5G23350	16.00847272	127.4656371	–2.9932	2.58E–19

AT3G55940	6.731768017	53.70188839	–2.9959	1.94E–08
AT1G04100	5.500347038	43.97634954	–2.9991	5.07E–07
AT3G19250	2.462841957	19.73297736	–3.0022	0.0017079
AT1G52240	11.24697827	90.16091322	–3.003	7.60E–14
AT3G29670	2.421794591	19.45107768	–3.0057	0.0018609
AT3G61060	36.69634516	295.1489614	–3.0077	1.67E–44
AT4G14630	17.97874629	144.6145341	–3.0078	5.73E–22
AT1G78830	62.26885415	501.0296918	–3.0083	2.02E–75
AT1G12610	13.79191496	111.5852886	–3.0163	4.11E–17
AT3G06500	65.83997499	533.3072048	–3.0179	1.55E–80
AT5G47740	15.14647804	122.7203259	–3.0183	8.91E–19
AT4G28290	3.735310302	30.68008147	–3.038	3.85E–05
AT2G47950	2.914362983	24.00845579	–3.0423	0.00036943
AT1G61820	80.20655308	670.7332971	–3.0639	7.18E–103
AT5G39610	8.291567923	69.72318667	–3.0719	4.55E–11
AT4G30430	2.627031421	22.12912461	–3.0744	0.00064163
AT2G04040	4.351020791	36.78790779	–3.0798	4.03E–06
AT1G25400	112.5518775	953.9015222	–3.0833	3.67E–147
AT2G38820	9.03042051	76.53576219	–3.0833	3.80E–12
AT1G80920	129.545487	1103.825167	–3.091	1.06E–170
AT1G30135	2.011320932	17.19588027	–3.0958	0.0032615
AT3G17690	5.746631234	49.19149356	–3.0976	4.96E–08
AT3G06490	5.089873379	43.78841643	–3.1048	3.15E–07
AT3G50970	236.4328279	2041.752374	–3.1103	0
AT5G52710	4.761494451	41.3452859	–3.1182	6.94E–07
AT5G46590	2.011320932	17.61872979	–3.1309	0.0026448
AT1G09460	26.31136158	231.1577348	–3.1351	1.93E–36
AT5G61390	4.761494451	41.86210197	–3.1362	5.29E–07

AT3G45060	3.571120838	31.43181394	−3.1378	2.08E−05
AT2G18700	83.65453182	738.5771526	−3.1422	3.31E−116
AT5G64250	81.84844772	726.5024498	−3.1499	1.34E−114
AT1G05680	19.74378302	176.0933313	−3.1569	5.00E−28
AT5G28145	3.530073472	31.76069689	−3.1695	1.65E−05
AT5G62350	79.59084259	716.9178608	−3.1711	7.50E−114
AT2G27690	3.899499766	35.37840941	−3.1815	4.35E−06
AT1G43670	41.90936064	380.7524965	−3.1835	8.89E−61
AT3G13430	21.38567766	194.8866431	−3.1879	2.73E−31
AT1G76600	35.21863999	321.6005477	−3.1909	1.80E−51
AT3G11020	3.817405034	35.00254317	−3.1968	4.68E−06
AT5G08790	104.3013569	958.9287331	−3.2007	1.23E−153
AT2G32150	129.8328185	1194.033063	−3.2011	2.82E−191
AT2G30395	3.160647179	29.12963325	−3.2042	3.76E−05
AT2G35420	1.682942004	15.64543205	−3.2167	0.004492
AT5G45630	4.06368923	37.82153994	−3.2183	1.56E−06
AT2G43120	85.29642646	799.7963657	−3.2291	2.78E−129
AT4G36010	21.63196186	202.9677671	−3.23	5.38E−33
AT3G12920	24.09480382	226.3654403	−3.2319	8.88E−37
AT1G66050	1.765036736	16.63208092	−3.2362	0.0030588
AT1G30760	16.95256214	160.5888491	−3.2438	2.86E−26
AT4G24380	30.90866656	295.1959446	−3.2556	3.25E−48
AT3G23240	1.806084102	17.28984683	−3.259	0.0023309
AT3G02800	11.94478349	114.4512687	−3.2603	6.33E−19
AT3G48450	18.63550414	180.4627763	−3.2756	9.10E−30
AT3G09350	32.63265593	318.6875844	−3.2878	1.65E−52
AT5G13080	18.84074097	184.0335055	−3.288	1.85E−30
AT5G44260	2.503889323	24.61923842	−3.2975	0.00014826

AT4G03510	18.55340941	184.1274721	–3.3109	1.13E–30
AT4G34380	4.884636549	48.48674437	–3.3113	1.99E–08
AT5G64510	2.052368298	20.39074327	–3.3126	0.00067851
AT2G18550	2.791220885	27.76711814	–3.3144	4.42E–05
AT5G05600	24.42318274	247.8837823	–3.3433	1.53E–41
AT4G01870	80.94540566	823.6638716	–3.347	9.12E–138
AT5G41080	217.0995185	2216.436207	–3.3518	0
AT3G20670	23.72537752	244.7359025	–3.3667	2.76E–41
AT2G13960	1.477705174	15.41051565	–3.3825	0.0037258
AT1G15010	21.79615132	232.0974004	–3.4126	1.15E–39
AT3G15500	14.48972018	154.4340395	–3.4139	1.68E–26
AT1G75860	26.47555104	283.4501248	–3.4204	1.75E–48
AT1G21120	30.82657183	334.8498325	–3.4413	1.52E–57
AT4G24230	82.75148977	900.4345502	–3.4438	1.48E–154
AT5G65600	1.682942004	18.32347898	–3.4446	0.0011241
AT5G25450	3.037505081	33.123212	–3.4469	3.85E–06
AT5G22300	6.280246991	68.87748764	–3.4551	3.40E–12
AT4G36410	10.34393622	114.2163523	–3.4649	5.81E–20
AT5G28630	21.75510396	240.5074074	–3.4667	1.18E–41
AT2G01340	7.142241676	79.21380912	–3.4713	5.25E–14
AT1G80160	21.63196186	240.2255077	–3.4732	1.13E–41
AT5G40000	9.276704706	103.4571813	–3.4793	3.42E–18
AT3G17110	36.81948726	413.7347586	–3.4902	5.89E–72
AT5G64530	2.873315617	32.74734576	–3.5106	3.62E–06
AT2G17840	218.8235079	2497.631134	–3.5127	0
AT1G14890	13.01201501	148.7020794	–3.5145	3.43E–26
AT2G32210	6.608625919	75.78402972	–3.5195	1.40E–13
AT4G18280	26.43450368	305.8141658	–3.5322	9.01E–54

AT5G55090	2.175510396	25.27700433	–3.5384	6.26E–05
AT5G40880	8.907278412	104.2089138	–3.5484	1.26E–18
AT5G14470	7.552715336	91.94627784	–3.6057	1.03E–16
AT1G05560	1.354563077	16.49113108	–3.6058	0.0017646
AT3G15356	4.186831327	51.11780802	–3.6099	1.63E–09
AT3G19390	181.7987838	2245.236957	–3.6265	0
AT3G09405	3.201694545	39.79483768	–3.6357	1.42E–07
AT5G14070	1.190373613	14.8467163	–3.6407	0.0031955
AT1G14260	2.011320932	25.27700433	–3.6516	4.80E–05
AT3G03660	2.13446303	26.92141911	–3.6568	2.44E–05
AT1G77380	12.23211505	156.5013038	–3.6774	1.32E–28
AT1G22400	9.112515242	117.2702655	–3.6858	1.47E–21
AT5G42830	4.392068157	56.52088515	–3.6858	1.21E–10
AT5G52310	90.34525247	1175.991484	–3.7023	1.29E–214
AT4G24040	2.750173519	35.98919204	–3.71	5.19E–07
AT1G08050	14.28448335	189.0137331	–3.726	7.47E–35
AT5G10625	18.01979365	239.5677418	–3.7328	3.80E–44
AT4G35480	12.27316242	164.770361	–3.7469	1.49E–30
AT5G41610	3.406931374	45.99663056	–3.755	6.95E–09
AT3G02480	2.627031421	35.47237597	–3.7552	5.59E–07
AT3G19240	124.4145662	1698.915384	–3.7714	0
AT5G03545	65.01902767	900.904383	–3.7924	1.63E–167
AT4G18170	2.832268251	39.37198816	–3.7971	9.67E–08
AT3G46080	4.638352353	64.97787545	–3.8083	1.86E–12
AT2G17880	4.392068157	62.86362787	–3.8393	3.85E–12
AT5G12340	7.265383774	105.1015961	–3.8546	4.87E–20
AT1G60750	4.06368923	58.96401568	–3.859	1.84E–11
AT1G63180	23.84851962	348.1930838	–3.8679	8.03E–66

AT5G67080	2.668078787	39.79483768	–3.8987	5.81E–08
AT5G06760	3.119599813	46.79534631	–3.9069	2.78E–09
AT2G32190	11.329073	170.9721538	–3.9157	9.38E–33
AT5G48570	13.42248867	203.7194996	–3.9239	5.11E–39
AT4G12735	1.559799906	23.67957283	–3.9242	5.30E–05
AT5G08350	32.38637174	492.8076179	–3.9276	3.87E–94
AT2G30400	3.037505081	46.41948007	–3.9338	2.97E–09
AT2G46240	13.91505706	213.4450384	–3.9391	5.72E–41
AT2G31081	0	14.56481662	–3.9474	0.0024821
AT1G69680	11.24697827	173.6032175	–3.9482	1.90E–33
AT3G28210	33.33046116	515.5005419	–3.9511	6.83E–99
AT1G10160	8.455757387	131.9760319	–3.9642	1.42E–25
AT3G47340	166.2007848	2616.968664	–3.9769	0
AT5G13220	3.078552447	48.48674437	–3.9773	1.03E–09
AT5G54165	1.272468345	20.1088436	–3.9821	0.00022488
AT2G26380	1.51875254	24.33733874	–4.0022	3.47E–05
AT1G72800	5.089873379	81.84487276	–4.0072	3.84E–16
AT1G74000	2.832268251	46.18456368	–4.0274	2.37E–09
AT4G22710	24.79260904	408.0967651	–4.0409	1.56E–79
AT1G54050	2.668078787	44.0703161	–4.0459	5.60E–09
AT1G74310	43.67439738	723.166637	–4.0495	5.18E–141
AT4G22690	29.10258246	483.1290624	–4.0532	2.54E–94
AT5G49620	2.791220885	46.3724968	–4.0543	1.99E–09
AT1G22985	6.321294357	105.3834958	–4.0593	7.97E–21
AT4G28085	10.50812568	175.8584149	–4.0648	1.47E–34
AT5G46330	1.067231515	17.90062946	–4.0681	0.00051597
AT1G15040	79.42665312	1357.252976	–4.0949	1.21E–266
AT5G57510	4.884636549	83.95912034	–4.1034	8.34E–17

AT5G19890	14.57181491	253.6157424	−4.1214	3.89E−50
AT5G24600	2.503889323	43.74143315	−4.1268	5.05E−09
AT4G35180	2.052368298	35.98919204	−4.1322	1.58E−07
AT1G59860	2.011320932	35.51935925	−4.1424	1.89E−07
AT5G65690	61.94047523	1105.328632	−4.1574	3.22E−219
AT4G01360	19.12807254	344.2464884	−4.1697	1.68E−68
AT5G49690	2.709126153	49.61434308	−4.1949	2.92E−10
AT2G40340	9.851367829	182.9059068	−4.2146	9.29E−37
AT2G41100	138.6990496	2588.073947	−4.2218	0
AT1G29395	1.477705174	27.76711814	−4.2319	5.03E−06
AT3G23550	1.559799906	29.55248276	−4.2438	2.21E−06
AT1G10170	85.62480538	1623.366271	−4.2448	0
AT5G48430	10.63126778	202.8268173	−4.2539	6.05E−41
AT5G28646	1.272468345	24.29035546	−4.2547	2.33E−05
AT5G06980	1.847131468	35.42539269	−4.2614	1.50E−07
AT4G25580	5.17196811	101.4369003	−4.2937	9.19E−21
AT5G57220	6.649673285	131.4592158	−4.3052	7.84E−27
AT3G12510	4.268926059	84.56990297	−4.3082	2.00E−17
AT4G16680	12.72468345	253.051943	−4.3137	1.96E−51
AT3G05630	2.503889323	50.08417587	−4.3221	1.56E−10
AT3G14210	0	13.20230152	−4.3293	0.0031264
AT4G21840	0	12.779452	−4.3754	0.0036406
AT2G47520	2.298652494	48.29881125	−4.3931	2.92E−10
AT1G01480	1.682942004	36.13014188	−4.4241	7.67E−08
AT1G15330	5.58244177	124.0358577	−4.4737	7.09E−26
AT5G02020	6.116057527	137.4260923	−4.4899	1.10E−28
AT5G39720	1.108278881	24.94812138	−4.4925	1.22E−05
AT4G20730	2.339699859	53.84283822	−4.5244	1.49E−11

AT1G73010	15.92637799	370.6041081	−4.5404	2.44E−77
AT5G52640	37.06577146	863.4587093	−4.542	5.85E−180
AT1G43160	22.94547757	535.0455861	−4.5434	1.29E−111
AT3G12320	0	16.35018124	−4.5503	0.00062894
AT1G71000	0	17.43079667	−4.5602	0.00037845
AT5G39120	1.108278881	26.40460304	−4.5744	5.54E−06
AT2G34390	1.354563077	32.51242936	−4.5851	3.11E−07
AT5G20150	27.95325622	672.1427955	−4.5877	7.76E−141
AT5G26340	30.8676192	747.4100091	−4.5977	1.01E−156
AT5G52670	1.108278881	26.96840239	−4.6049	4.10E−06
AT1G56060	0	17.33683011	−4.6349	0.00037166
AT5G38410	2.585984055	64.74295905	−4.6459	5.84E−14
AT3G02040	16.54208848	428.1116421	−4.6938	1.01E−90
AT1G63530	7.265383774	188.3559672	−4.6963	4.91E−40
AT2G39400	6.116057527	158.897451	−4.6994	7.64E−34
AT5G43360	0	13.39023464	−4.7431	0.0021885
AT2G17660	5.418252306	145.4602331	−4.7467	3.89E−31
AT2G30750	0	15.64543205	−4.7669	0.00074326
AT3G46090	1.354563077	36.88187435	−4.767	2.91E−08
AT1G64160	4.802541817	131.0833496	−4.7705	3.81E−28
AT1G56250	3.817405034	106.8869607	−4.8073	4.36E−23
AT5G39150	0	15.31654909	−4.8431	0.0008287
AT4G36820	1.108278881	33.02924544	−4.8974	1.57E−07
AT2G44578	2.011320932	60.93731342	−4.9211	1.81E−13
AT1G66090	2.709126153	83.39532098	−4.9441	2.71E−18
AT5G52050	23.06861967	721.4282556	−4.9669	3.99E−156
AT3G44870	0	12.87341856	−4.971	0.0024981
AT2G05580	0	31.43181394	−4.9958	3.06E−07

AT5G43650	0	18.37046226	–4.9985	0.00017568
AT1G59660	14.81809911	473.7793897	–4.9988	7.98E–103
AT3G53600	0	14.6117999	–5.0162	0.0010586
AT5G24640	1.149326247	37.53964026	–5.0296	1.47E–08
AT1G77120	18.59445678	609.6080506	–5.0349	1.51E–132
AT2G24130	2.462841957	81.32805669	–5.0454	5.73E–18
AT1G69490	12.39630452	427.923709	–5.1094	1.62E–93
AT1G26380	0	22.73990724	–5.1137	1.94E–05
AT1G21529	0	17.33683011	–5.1158	0.00027412
AT4G37370	4.022641864	143.6278852	–5.158	1.23E–31
AT5G39580	23.06861967	834.657959	–5.1772	7.56E–183
AT3G60120	0	11.98073625	–5.1892	0.0035263
AT1G52890	0	16.86699731	–5.2233	0.00032863
AT1G16030	7.265383774	284.4837569	–5.2912	8.93E–63
AT2G36770	0	19.68599408	–5.2991	7.89E–05
AT3G47380	1.51875254	60.37351406	–5.313	1.24E–13
AT4G06746	4.022641864	160.4948825	–5.3182	1.42E–35
AT4G34210	0	20.01487704	–5.323	6.66E–05
AT3G28600	0	23.02180692	–5.3241	1.51E–05
AT5G33355	15.02333594	617.8301245	–5.3619	1.57E–136
AT2G36800	0	15.31654909	–5.3737	0.00066628
AT2G22860	19.1691199	795.0040712	–5.3741	1.19E–175
AT1G06225	0	20.76660951	–5.3762	4.51E–05
Novel00001	1.477705174	62.53474492	–5.4032	3.78E–14
AT5G38430	0	21.14247574	–5.4237	3.68E–05
AT1G02920	12.60154135	558.5372258	–5.47	6.93E–124
AT5G01380	3.940547132	175.3415988	–5.4756	4.81E–39
AT5G26000	0	22.27007445	–5.477	2.06E–05

AT5G59080	2.216557762	102.9873485	–5.538	4.16E–23
AT5G20230	22.78128811	1122.289596	–5.6225	9.36E–250
AT5G01760	0	12.40358577	–5.6543	0.0026407
AT3G19615	0	39.32500488	–5.656	3.84E–09
AT1G26240	1.067231515	55.44026972	–5.699	1.11E–12
AT1G71520	0	44.02333282	–5.7448	3.51E–10
AT5G43370	0	19.8739272	–5.7494	6.44E–05
AT1G07400	2.503889323	136.0165939	–5.7635	1.58E–30
AT1G11925	0	27.34426863	–5.7732	1.54E–06
AT4G13420	0	11.22900378	–5.7738	0.0046361
AT3G60420	0	22.5989574	–5.7828	1.65E–05
AT3G22740	1.436657808	79.68364191	–5.7935	4.75E–18
AT2G11810	0	39.0431052	–5.8061	4.28E–09
AT4G33720	2.832268251	158.6625346	–5.8079	1.45E–35
AT1G63040	0	35.37840941	–5.8445	2.70E–08
AT1G19250	0	19.4040944	–5.8849	8.05E–05
AT1G70130	0	38.90215537	–5.8883	4.56E–09
AT5G52400	0	12.49755233	–5.9282	0.0024878
AT1G32970	0	18.0415793	–5.9725	0.00015963
AT1G14550	0	21.7062751	–6.0466	2.57E–05
AT2G24850	0	28.23695094	–6.1042	9.88E–07
AT1G10155	0	11.69883658	–6.1549	0.0036893
AT2G43620	0	40.8754531	–6.2593	1.81E–09
AT5G62360	1.436657808	113.2766867	–6.301	2.16E–25
AT4G33467	0	48.43976109	–6.3973	4.30E–11
AT2G02250	0	14.8467163	–6.4986	0.00083387
AT3G22910	6.854910115	650.2955706	–6.5678	2.09E–143
AT1G02930	5.705583868	573.4309254	–6.6511	4.16E–126

AT3G16120	0	29.88136572	–6.7004	5.46E–07
AT1G17710	0	22.50499085	–6.7768	2.15E–05
AT3G03670	0	31.90164673	–6.7948	2.16E–07
AT3G27690	0	95.70494019	–6.7948	3.77E–21
AT2G31345	0	14.70576646	–6.8999	0.0010028
AT4G21920	0	86.35526759	–6.9513	5.54E–19
AT3G16530	1.108278881	140.8558717	–6.9898	1.13E–30
AT3G44860	0	21.04850918	–7.0022	4.91E–05
AT4G32950	1.149326247	152.3197919	–7.0502	4.86E–33
AT2G15780	0	28.47186733	–7.1161	1.47E–06
AT1G75600	0	84.38196985	–7.1981	2.60E–18
AT5G55150	0	37.35170714	–7.2447	2.31E–08
AT2G10940	0	19.4040944	–7.2999	0.00012894
AT5G14740	0	20.24979343	–7.3614	8.97E–05
AT4G36600	0	13.71911759	–7.3847	0.0019669
AT3G19920	0	34.57969366	–7.3965	1.04E–07
AT2G42560	0	92.41611063	–7.4362	1.07E–19
AT3G09922	0	58.58814945	–7.4791	1.29E–12
AT1G34047	0	149.8296781	–7.5858	2.33E–31
AT1G48710	0	15.83336517	–7.5915	0.00081584
AT4G33930	0	116.3775831	–7.6619	2.76E–24
AT3G63380	6.239199625	1289.878953	–7.6917	4.42E–266
AT5G61890	0	17.14889699	–7.7066	0.00048079
AT1G80660	0	42.56685116	–8.0182	6.28E–09
AT4G37710	0	21.98817477	–8.0652	7.06E–05
AT1G69930	0	67.84385549	–8.1058	8.28E–14
AT5G64750	1.600847272	527.8571444	–8.3652	2.31E–102
AT2G38240	2.011320932	680.2709028	–8.4018	2.37E–131

AT1G69920	0	133.9493296	−8.5022	8.41E−26
AT1G05675	0	79.40174223	−8.5957	2.56E−15
AT3G55970	0	36.0831586	−8.7798	4.11E−07
AT3G01500	0	38.71422225	−8.8814	1.66E−07
AT5G12030	0	214.3377207	−8.8909	2.38E−39
AT5G38420	0	48.06389485	−9.1934	7.22E−09
AT4G25200	0	29.59946604	−9.4941	1.73E−05
AT2G02010	0	535.4214524	−9.5012	9.20E−91
AT3G46230	0	679.8950366	−9.8458	6.77E−110
AT3G43250	0	38.1504229	−9.8602	1.36E−06
AT1G53540	0	256.8106054	−10.026	1.37E−40
AT2G36255	0	102.8933819	−10.292	6.87E−16
AT5G12020	0	108.9072417	−10.374	1.38E−16
