# Supplementary material for: Expression of Root Genes in Arabidopsis Seedlings Grown by Standard and Improved Growing Methods
Source: Int J Mol Sci. 2017 May 3;18(5):951. doi: 10.3390/ijms18050951 (PMC5454864; doi:10.3390/ijms18050951)
Supplement: Supplementary file 1 [file ijms-18-00951-s001.zip › Table S3.pdf]

Supplementary Table 3. List of all 141 PEGs from IPG/TPG comparison groups.

Gene_id	readcount_TPG	readcount_IPG	$\log_2$ Fold change (TPG vs IPG)	q value
AT3G22142	16.96262238	0	4.738	0.0020806
AT5G04120	22.83263929	1.662408091	3.7798	0.00041635
AT1G08090	40.18371867	3.227027471	3.6383	2.97E–07
AT5G17220	20.07027839	1.955774225	3.3592	0.0028957
AT4G31940	99.66080173	12.32137762	3.0159	8.21E–16
AT4G15480	191.6819491	29.87445129	2.6817	2.38E–27
AT5G62210	233.721629	38.38206917	2.6063	1.93E–32
AT3G28160	50.11095314	9.583293703	2.3865	6.12E–06
AT3G60140	139.9308442	27.08747302	2.369	3.76E–17
AT5G15960	117.9182808	23.51818506	2.3259	4.41E–14
AT1G44575	30.60177931	6.356266231	2.2674	0.0025789
AT5G24770	91.41688093	19.06879869	2.2612	1.90E–10
AT3G22840	262.5969327	57.10860737	2.2011	7.64E–30

AT4G37800	72.33932598	16.28182042	2.1515	1.13E–07
AT5G07990	513.8854506	117.1019817	2.1337	6.72E–57
AT5G17700	32.71671187	7.480836411	2.1288	0.0028192
AT2G34810	35.17693954	8.60540659	2.0313	0.0025671
AT2G22590	178.0859541	45.22727895	1.9773	1.96E–17
AT5G15970	332.4328692	85.36954492	1.9613	1.10E–32
AT1G10370	167.8565864	43.90713135	1.9347	5.93E–16
AT2G23910	539.9552315	142.0381031	1.9266	1.81E–52
AT4G14690	68.62740353	18.77543256	1.8699	5.62E–06
AT1G65060	256.295297	76.42187784	1.7457	2.43E–21
AT4G04840	104.7107427	32.71032391	1.6786	5.44E–08
AT5G17040	59.95186384	18.82432692	1.6712	0.0002134
AT4G19810	55.20405605	17.4063906	1.6652	0.00051027
AT5G44400	76.91448622	24.44717781	1.6536	1.23E–05
AT4G03210	102.8979434	32.71032391	1.6534	1.14E–07
AT4G35770	136.8231882	44.39607491	1.6238	4.58E–10

AT5G49360	326.3902047	106.5408009	1.6152	1.90E–24
AT4G02290	184.1286185	60.23784613	1.612	1.57E–13
AT5G48880	403.4773385	134.4105836	1.5858	1.68E–29
AT4G04750	52.61434271	17.60196803	1.5797	0.0015494
AT1G01580	152.7930871	51.6813339	1.5639	1.19E–10
AT1G77760	226.8157268	77.05750447	1.5575	6.10E–16
AT1G30530	74.58374421	25.76732541	1.5333	7.23E–05
AT3G55120	712.5596257	248.9211645	1.5173	1.70E–49
AT2G18150	112.2209114	39.31106192	1.5133	2.46E–07
AT3G21560	389.6223721	137.9309772	1.4981	3.41E–26
AT3G53980	64.39753841	22.93145279	1.4897	0.00054362
AT1G42550	108.120532	38.96880143	1.4722	9.08E–07
AT2G38230	109.6311981	39.70221677	1.4654	8.22E–07
AT5G56080	53.08912348	19.31327047	1.4588	0.0037936
AT1G06000	172.8202036	63.85602845	1.4364	1.26E–10
AT5G17050	295.7021016	117.3953479	1.3328	1.85E–16

AT1G22770	72.64145921	28.84766982	1.3323	0.00084469
AT1G78290	106.9119991	43.71155393	1.2903	2.06E–05
AT5G05270	784.6831422	322.7027471	1.2819	3.99E–42
AT5G08640	1575.840568	648.9747822	1.2799	3.77E–85
AT3G53260	905.709079	373.6017713	1.2775	1.63E–48
AT4G11650	202.9472021	84.19608039	1.2693	2.54E–10
AT1G21400	112.9546635	47.62310238	1.246	2.06E–05
AT3G23810	923.0169965	389.1990708	1.2458	1.50E–47
AT1G06430	82.35288423	35.20393605	1.2261	0.00086896
AT1G55020	313.830095	135.0462102	1.2165	3.92E–15
AT2G33770	183.8264853	79.1110674	1.2164	1.12E–08
AT1G10960	82.13707479	35.35061912	1.2163	0.0009985
AT2G25160	79.07258067	34.17715458	1.2101	0.0015022
AT2G25530	76.48286733	33.24816183	1.2019	0.0022297
AT5G52310	94.99931772	41.41351922	1.1978	0.00032128
AT4G30470	199.494251	87.52089657	1.1886	4.22E–09

AT1G10070	125.8169065	55.34841057	1.1847	1.41E–05
AT4G16260	131.6005996	58.1842832	1.1775	8.80E–06
AT1G24280	111.7461307	49.43219354	1.1767	7.23E–05
AT3G47780	89.5609197	39.80000548	1.1701	0.00079283
AT3G04720	126.4211729	56.47298075	1.1626	1.98E–05
AT1G78570	1198.174039	536.7622361	1.1585	4.66E–55
AT5G04950	95.99204116	43.66265957	1.1365	0.00064475
AT3G47340	174.7624886	80.72458114	1.1143	4.08E–07
AT4G15530	120.9827749	56.42408639	1.1004	9.99E–05
AT5G13930	5369.080022	2523.046539	1.0895	5.86E–228
AT1G30510	336.6195724	160.6179582	1.0675	4.14E–13
AT3G44300	526.4887222	253.2238678	1.056	2.21E–20
AT1G79040	163.4540737	78.6710182	1.055	4.99E–06
AT5G10470	113.7315775	55.00615008	1.048	0.00045244
AT3G49120	431.3599188	208.8766872	1.0462	2.24E–16
AT4G09750	89.86305292	43.51597651	1.0462	0.003614

AT1G55320	149.9444024	72.80369553	1.0423	2.10E–05
AT5G42270	206.09802	100.7223726	1.0329	1.97E–07
AT5G01600	126.5074967	62.19362036	1.0244	0.00022604
AT2G37040	2093.610588	1041.84093	1.0069	5.00E–77
AT5G13630	93.31600404	46.59632091	1.0019	0.0047098
AT1G32640	270.7113679	543.3140797	–1.005	3.77E–19
AT1G13260	120.2490228	241.5381168	–1.0062	2.71E–08
AT2G22330	94.69718449	192.0081345	–1.0198	1.13E–06
AT4G37610	83.34560768	171.6191882	–1.042	0.00000384
AT2G06050	43.16188901	88.93883288	–1.0431	4.46E–03
AT3G23430	216.9316542	447.0899878	–1.0433	1.12E–16
AT1G20510	90.89893826	189.6123111	–1.0607	4.72E–07
AT5G22920	64.0090814	133.6282739	–1.0619	7.04E–05
AT1G78280	63.79327196	133.3838021	–1.0641	6.94E–05
AT1G73080	72.85726865	153.8216428	–1.0781	8.36E–06
AT2G42760	73.4615351	155.7285227	–1.084	0.00000616

AT1G19180	306.449412	650.4905072	–1.0859	2.99E–26
AT4G17500	95.60358416	204.1828291	–1.0947	4.84E–08
AT2G32150	136.5210549	295.1752249	–1.1124	3.95E–12
AT1G66760	47.69388736	103.5093509	–1.1179	4.52E–04
AT5G18670	69.01586053	150.1545661	–1.1214	0.00000475
AT3G25190	125.0399925	273.4172367	–1.1287	1.76E–11
AT4G15760	47.00329713	104.7806041	–1.1565	2.31E–04
AT5G01040	111.4008355	248.4322209	–1.1571	7.97E–11
AT5G48412	70.26755531	161.0091131	–1.1962	2.84E–07
AT2G25460	110.5375978	254.6906985	–1.2042	7.18E–12
AT5G42650	57.57795994	136.1218861	–1.2413	1.74E–06
AT5G41080	228.283231	546.2966354	–1.2589	5.98E–28
AT4G17615	34.65899688	83.56045376	–1.2696	5.09E–04
AT1G49500	36.04017732	87.71647399	–1.2832	0.00026222
AT4G21850	85.93532102	210.1479405	–1.2901	7.11E–11
AT2G14878	78.38199045	192.9860217	–1.2999	4.36E–10

AT1G70850	1114.094679	2766.247064	–1.3121	1.99E–155
AT2G01520	402.8299101	1003.263283	–1.3165	8.76E–56
AT1G80840	141.182539	352.6749871	–1.3208	2.91E–19
AT3G15630	58.00957883	150.4479323	–1.3749	1.64E–08
AT2G18210	24.25698162	66.15406316	–1.4474	6.81E–04
AT3G59930	76.95764811	213.9617002	–1.4752	1.04E–13
AT1G26250	78.166181	221.491431	–1.5026	1.29E–14
AT5G19120	98.92704962	281.2403336	–1.5074	9.36E–19
AT5G05600	25.68132396	73.39042779	–1.5149	1.17E–04
AT5G19110	19.07755494	55.54398799	–1.5418	1.41E–03
AT1G32450	108.2068558	323.778423	–1.5812	2.64E–23
AT1G72450	43.46402223	131.2324505	–1.5942	2.76E–09
AT3G09940	65.73555696	201.1024847	–1.6132	1.03E–14
AT5G63160	64.26805274	197.9732459	–1.6231	1.29E–14
AT1G79310	11.86951948	40.68010388	–1.7771	3.48E–03
AT2G46510	17.73953638	60.97126147	–1.7812	0.0000688

AT4G34410	66.55563286	229.7056827	–1.7872	1.44E–19
AT1G11185	14.41607093	50.06782016	–1.7962	5.08E–04
AT1G12610	14.50239471	51.14349598	–1.8183	0.000347
AT4G21830	34.40002554	122.1381004	–1.828	1.53E–10
AT2G25735	15.36563249	54.56610088	–1.8283	1.63E–04
AT3G14260	11.86951948	44.78722975	–1.9158	6.25E–04
AT1G74930	23.04844873	89.77003693	–1.9616	1.91E–08
AT4G12550	21.66726828	90.89460711	–2.0687	3.44E–09
AT1G17420	9.884072584	42.09804019	–2.0906	3.84E–04
AT5G12340	7.639654355	34.07936587	–2.1573	1.82E–03
AT5G33355	15.79725138	75.88403993	–2.2641	1.44E–08
AT1G28480	20.28608784	105.5140194	–2.3789	7.56E–13
AT1G74950	30.34280798	160.5690639	–2.4038	5.14E–20
AT2G34600	8.891349136	47.3786306	–2.4138	0.000012
AT5G13220	3.237141676	23.51818506	–2.861	0.0027735
