# Supplementary material for: Expression of Root Genes in Arabidopsis Seedlings Grown by Standard and Improved Growing Methods
Source: Int J Mol Sci. 2017 May 3;18(5):951. doi: 10.3390/ijms18050951 (PMC5454864; doi:10.3390/ijms18050951)
Supplement: Supplementary file 1 [file ijms-18-00951-s001.zip › Table S4.pdf]

Supplementary Table 4. List of the primers for the qRT-PCR analysis to valid the transcriptome results.

Gene Name	Gene ID	Primer Sequences (F/R)	Product Length
ATFRO2	AT1G01580	CTCATCAATCCTCGGACCA/ TGCGGCTATGTTGTGGAAC	189
/	AT1G21400	AATGGGTGAGGCTCCGTTA/ GGACCTCATAAGTTGCGGTT	178
ASN1	AT3G47340	AGAACTCTGCGAGACTAACGG/ TGGGTCAAGGAGTTGTGATTC	183
LAX3	AT1G77690	TGGGTCAAGGAGTTGTGATTC/ GGGTATTCGTAGTTGGGTT	201
GH3.17	AT1G28130	TACCAAGTTACGACCCAA/ CGTAAAGCCTTTCATTCA	188
ATCDPK2	AT1G35670	TGGACCTGAAATAGATGTG/ TCCAGCAGTCTTATCTCG	196

UBC5	AT1G63800	TCAGTGGACCCAAAGAC/<br>TTTCTTCCGCAACTTCT	173
ARR3		TGCCTGGAATGACTGGA/<br>AAGAAGGTGCGGAGGAT	178
ATNPR1	AT1G64280	TTGCGGAGAAGACGACA/<br>CTCATCGTCGTCGGTGA	196
ERS2	AT1G04310	GATTGTTAAGGTGGTGGC/<br>GATTGTTAAGGTGGTGGC	182
ACTIN2	AT3G18780	AAGCTCTCCTTTGTTGCTGTT/<br>GACTTCTGGGCATCTGAATCT	178
