# Supplementary material for: Expression of Root Genes in Arabidopsis Seedlings Grown by Standard and Improved Growing Methods
Source: Int J Mol Sci. 2017 May 3;18(5):951. doi: 10.3390/ijms18050951 (PMC5454864; doi:10.3390/ijms18050951)
Supplement: Supplementary file 1 [file ijms-18-00951-s001.zip › Table S5.pdf]

Supplementary Table 5. List of the primers for the qRT-PCR analysis on photoreceptor genes.

Gene Name	Gene ID	Primer Sequences (F/R)	Product Length
UVR8	AT5G63860	ATTGTTTCCGTTACCTGTGGTGCTG/ AATCTGCTTGATCCGAATACCGTGC	189
CRY1	AT4G08920	AAACCAAGTCACTGCTATGATTCCA/ TCCTCCAATACCATTTTCTTCACTA	178
CRY2	AT1G04400	GTTGTCTACTTTTGTCTCGTTGTGA/ TAGTGACTTGACTCTTCTTTCTTCC	183
PHOT1	AT3G45780	AAGAGCCCCTCCATCTCCTTTGAAT/ GCGGTTCTTTCTGTAATCTTAGCGG	201
PHOT2	AT5G58140	ATGCGTGTGGTATCTATGTCTATCC/ AGTTGACGGGTTGAATACTTCGAGA	188
PHYA	AT1G09570	TTGTATGGAGACAGTATTAGGCTTC/ TTAGTAAAAACTCAGGTATCCCAGC	196

PHYB	AT2G18790	ATGGTTTCCGGAGTCGGGGGTAGT/ ATTGCTTTGCTCATTGATTCAGTGTTG	173
ACTIN2	AT3G18780	AAGCTCTCCTTTGTTGCTGTT/ GACTTCTGGGCATCTGAATCT	178
