# Supplementary figures and images for: Expression of Root Genes in Arabidopsis Seedlings Grown by Standard and Improved Growing Methods
Source: Int J Mol Sci. 2017 May 3;18(5):951. doi: 10.3390/ijms18050951 (PMC5454864; doi:10.3390/ijms18050951)

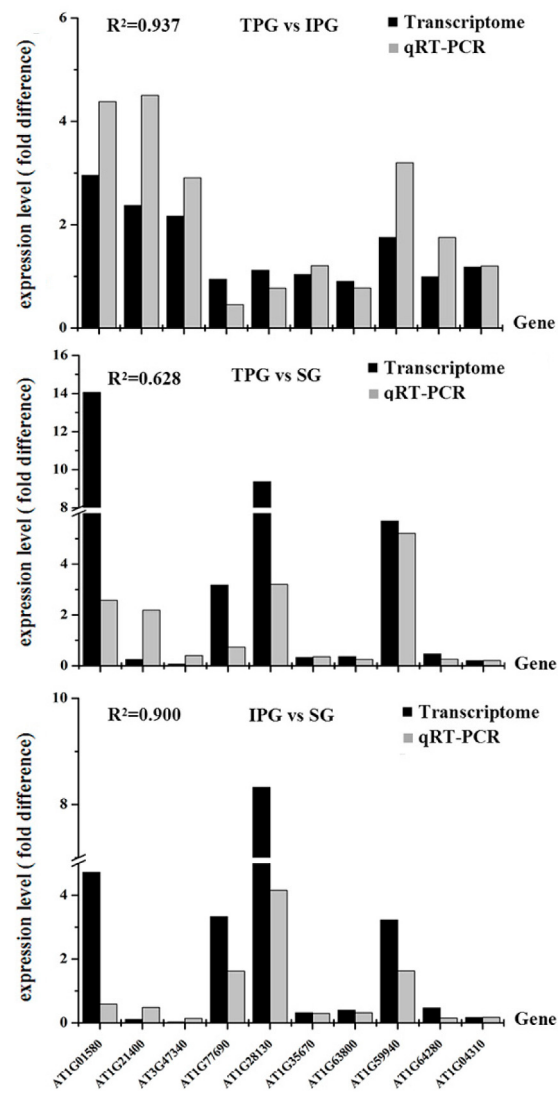

**Figure S1.** Validation of the transcriptome results by qRT-PCR.

Supplement: Supplementary file 1 [file ijms-18-00951-s001.zip › Figure S1.pdf]
